# Androgen-targeted hsa_circ_0085121 encodes a novel protein and improves the development of prostate cancer through facilitating the activity of PI3K/Akt/mTOR pathway and enhancing AR-V7 alternative splicing

**DOI:** 10.1038/s41419-024-07246-9

**Published:** 2024-11-20

**Authors:** Jianfeng Li, Hui Qiu, Qingzhuo Dong, Hongyuan Yu, Chiyuan Piao, Zhengxiu Li, Yanbin Sun, Xiaolu Cui

**Affiliations:** 1 Department of Urology, First Hospital of China Medical University, #155 Nanjing North Road, 110001 Shenyang, China; 2 Department of Gynecology and Obstetrics, Shengjing Hospital of China Medical University, #36 Sanhao Street, 110004 Shenyang, China; 3 Department of Dermatology, First Hospital of China Medical University, #155 Nanjing North Road, 110001 Shenyang, China; 4 Department of Thoracic Surgery, First Hospital of China Medical University, #155 Nanjing North Road, 110001 Shenyang, China

**Keywords:** Prostate cancer, Transcriptomics

## Abstract

Prostate cancer (PCa) is the most prevalent type of cancer and the second leading cause of mortality in males, with a marked increase in incidence observed across the globe. In the present study, whole-transcriptome analysis was conducted to identify differentially expressed circular RNAs (DE-circRNAs). The coding abilities of the DE-circRNAs were analyses, and it was found that hsa_circ_0085121 (circRNF19A) not only exhibited overexpression in PCa cells and tumor samples, but also encoded a 490 amino acid polypeptide designated circRNF19A-490aa. The knockdown of circRNF19A was observed to notably inhibit the proliferation, invasion, migration and docetaxel resistance of PCa cells. In contrast, mutation of the IRES significantly impaired the tumor-promoting function of circRNF19A, indicating that circRNF19A-490aa is the primary form that regulates the malignant behaviors of PCa cells. Mechanistically, circRNF19A-490aa was demonstrated to interact with HSP90AA1, thereby enhancing AR activity and facilitating the activation of the Akt/mTOR and PLK1 pathways. Furthermore, circRNF19A-490aa was observed to interact with HNRNPF, facilitating the recruitment of HNRNPF to the splicing site of AR-V7 and enhancing its alternative splicing. Finally, the androgen receptor (AR) was observed to bind to the promoter region of the RNF19A gene, subsequently regulating the expression of circRNF19A and circRNF19A-490aa. These data indicate that circRNF19A plays a pivotal role in AR activation and AR-V7 generation by encoding a novel protein, circRNF19A-490aa, and targeting circRNF19A may prove an effective strategy for impeding the progression of CRPC.

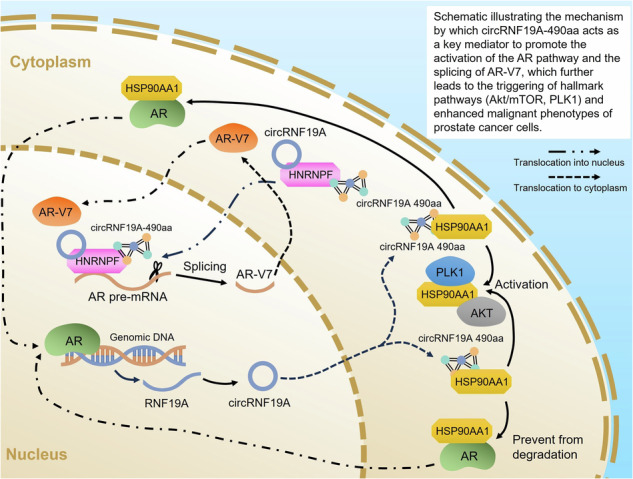

## Introduction

Prostate cancer (PCa) is one of the most common diseases worldwide, with 1,414,259 new cases and 375,304 deaths in 174 countries occurring from 2000 to 2019 [1, 2]. PCa is also the most common type of cancer in men [2]. The development and progression of prostate cancer are complex processes that involve a range of hereditary and environmental variables [3–6]. One critical element in the development of PCa is alterations in the androgen signaling pathway [6, 7]. The androgen receptor (AR) is a ligand-induced transcription factor that is predominantly expressed in primary prostate cancer and metastatic tumors. AR affects a wide variety of cellular activities, including proliferation, apoptosis, migration, invasion, and differentiation, and the expression of AR in prostate cancer is regulated by steroids and peptide hormones [7, 8]. Therefore, androgen deprivation therapy (ADT) and AR pathway suppression therapy have been widely used clinically. Unfortunately, treatment resistance occurs after hormone therapy in the majority of PCa patients [7, 9]. There are several possible mechanisms for this, including constitutively active AR variant expression, AR mutation, intratumor androgen synthesis and amplification of the AR gene [10–12]. Notably, specific AR mutations are frequently discovered after the emergence of therapeutic resistance and may change the ligand binding affinity and alter the prostate cancer response to AR inhibitors (ARIs) [9, 13], resulting in constitutive activation of the AR pathway and progression to castration-resistant prostate cancer (CRPC), which is the final stage and a lethal form of PCa [13, 14].

As a second-generation ARI, the clinical use of enzalutamide has significantly improved the prognosis of CRPC patients [15, 16]. Data from a clinical trial showed that enzalutamide prolonged the median survival of CRPC patients to 18.4 months, while the median survival in the placebo group was 13.6 months [17]. However, resistance to enzalutamide still occurs after a median of 11.2 years of treatment [18]. Androgen receptor variant 7 (AR-V7) is the major variant generated from AR pre-mRNA by alternative splicing (AS). This particular form of AR is constitutively activated at the transcriptional level and does not require ligand binding to activate the transcription of AR target genes [19]. Therefore, AR-V7 has been recognized as the major cause of enzalutamide resistance [20]. Previously, we reported that the histone demethylase lysine demethylase 3 A (KDM3A) cooperates with heterogeneous nuclear ribonucleoprotein F (HNRNPF) to enhance AR-V7 splicing [21]. Although studies have focused on the generation of AR-V7, little is known about whether circular RNAs are involved in AR-V7 alternative splicing and drug resistance.

Heat shock proteins function as chaperones and protect their client proteins from degradation [22, 23]. In human cancers, heat shock proteins are often upregulated and are always linked with tumor progression, as many of these proteins contribute to tumorigenesis, angiogenesis, apoptotic resistance and cancer metastasis [24, 25]. By maintaining the stability of client proteins such as the AR, estrogen receptor (ER) and MYC, heat shock proteins modulate the activity of different pathways, including the PI3K/Akt, JAK/STAT3, PKL1 and MAPK pathways, eventually leading to uncontrolled cell growth, persistent angiogenesis, apoptosis evasion, tumor invasion and metastasis [26–28]. Heat shock protein 90 (HSP90) belongs to the heat shock protein family and has been identified as a promising therapeutic target for prostate cancer treatment [27, 29, 30]. In prostate cancer cells, HSP90 interacts with AR-FL and AR-V7 and sustains their stability and ligand-binding ability, and HSP90 expression correlates with PCa progression and the levels of AR-FL and AR-V7 [31]. Inhibition of HSP90 leads to the proteasomal degradation of AR-FL and AR-V7 and hampers the tumor growth and metastasis of CRPC cells [31]. Therefore, targeting HSP90 is recognized as a dual-targeting strategy for both AR and AR-V7 and has shown some clinical activity in patients with metastatic CRPC [32]. Although HSP90 inhibitors have been intensively used in preclinical and clinical studies against PCa, whether noncoding RNAs regulate the activity of HSP90 in PCa cells and what the underlying mechanisms may be remain to be elucidated.

Transcriptomic analysis has revealed new biomarkers, including noncoding RNAs (ncRNAs), for the diagnosis and treatment of prostate cancer [33–35]. Based on their size cutoff of 200 nucleotides, ncRNAs are divided into two highly diverse groups: small ncRNAs (sncRNAs) and long ncRNAs (lncRNAs) [35]. Among them, circular RNAs (circRNAs) have been extensively studied in recent years as viable diagnostic and prognostic biomarkers for human cancers [36, 37]. circRNAs are a family of single-stranded noncoding RNAs generated in circular conformations by noncanonical splicing or reverse splicing. Abnormal expression of circRNAs has been frequently observed in prostate cancer and other cancer types, suggesting that circRNAs may play a critical role in the genesis of human cancers [37, 38]. Mechanistically, circRNAs may function as miRNA sponges to regulate the transcriptional stability of target genes. Moreover, circRNAs may interact with RNA-binding proteins (RBPs) and influence the biological functions of target proteins [39, 40]. In addition, emerging evidence has shown that specific circRNAs may attach to ribosomes and be translated into multiple peptides to facilitate cancer growth [41, 42]. circRNAs are associated with cancer initiation and progression and are multifunctional molecules with great research potential [43, 44].

In the present study, we investigated novel onco-circRNAs with protein-coding capabilities and collected clinical tissue samples from a cohort of five PCa patients for transcriptome analysis. Our data revealed a large number of differentially expressed (DE) mRNAs, circRNAs, miRNAs and lncRNAs. The protein-coding potential of the DE-circRNAs was predicted by analyzing the open reading frames (ORFs) and internal ribosome entry sites (IRESs) in the genes. Among them, circRNF19A was found to be significantly overexpressed in PCa tissues and encodes a novel protein of 490 amino acids. Functional analysis revealed that the novel protein circRNF19A-490aa had strong oncogenic effects on PCa cells. Immunoprecipitation and LC‒MS analysis were performed to investigate the proteins that potentially interact with circRNF19A-490aa in 22Rv1 cells, and the chaperone protein HSP90AA1 and the RNA binding protein HNRNPF were identified. Our study revealed the biological functions and mechanism of the novel protein circRNF19A-490aa in enhancing the proliferation and drug resistance of PCa cells and stimulating CRPC phenotypes.

## Materials and methods

### Clinical patient specimens and tissues

Fresh clinical samples comprising both prostate cancer tissues and adjacent normal prostate tissues were recently acquired from a cohort of five patients who underwent radical surgical resection following a confirmed pathological diagnosis of prostate cancer. circRNF19A expression was detected in PCa tumor samples from another cohort of thirty PCa patients. All patients were hospitalized and treated at the Urology Department of the First Hospital of China Medical University in Shenyang, China. The study adhered to a protocol (2012–33) approved by the institutional review board through the Medical Ethics Committee of the First Hospital of China Medical University (authorization number: AF-SOP-07-1.2-01). Written informed consent was obtained from each patient for both surgery and research purposes. Clinicopathological sections of normal prostate tissue and prostate cancer tissues were obtained from the Department of Pathology at the First Hospital of China Medical University.

### Application of omics techniques and bioinformatic analysis

DNA/RNA/small RNA/cDNA library sequencing was performed on an Illumina HiSeqTM 2500/4000 by Gene Denovo Biotechnology Co., Ltd. (Guangzhou, China). 4D label-free proteomics analysis was performed by Genechem Co., Ltd. (Shanghai, China). Detailed information on the transcriptomic and proteomic analyses is provided in the supplementary information.

Gene expression patterns and clinicopathological features of prostate cancer patients were collected from the TCGA_PRAD dataset. R software (v4.1.3, R core team, March 10, 2022) was used to analyze the RNA sequencing (RNA-seq) data. Gene expression was normalized using the DESeq2 package (v1.36.0; Michael Love, March 15, 2022). The read counts were normalized to the transcripts per million (TPM) values. This study complied with the publication guidelines provided by the TCGA.

KM survival analysis was performed utilizing the GEPIA online analytic tool (http://gepia.cancer-pku.cn/). CircBase (http://www.circbase.org/) was used for the validation of annotations in the circRNF19A prediction, while JASPAR (https://jaspar.elixir.no/) was used to predict the probable existence of androgen receptor (AR) binding sites within the upstream promoter region of RNF19A. The tool catRAPID (http://s.tartaglialab.com/page/catrapid_group) was used to predict the binding motifs on the RNF19A pre-mRNAs.

### Antibodies and reagents

The antibodies and reagents used in this study are listed in Supplementary file (Table S11).

### Cell culture

RWPE-1, PC3, DU145, LN95, 22Rv1 and LNCaP cells were purchased from the cell bank of the Chinese Academy of Sciences (Shanghai, China). Regular testing was conducted to ensure that the cells remained free of mycoplasma contamination. All cell lines were cultured in 5% CO_2_ at 37°C in a humidified atmosphere. When the cells reached more than 80% confluence, they were washed with 1× PBS and trypsinized at 37°C for a specified number of minutes for cell passage cultivation.

### In vivo tumor xenograft model

Male BALB/c (nu/nu) mice aged 6 weeks and weighing 20–24 g were acquired from SPF (Beijing) Biotechnology Co., Ltd. (Beijing, China). The mice were maintained in a sterile SPF room at a regulated temperature (24 ± 2 °C) with ad libitum access to food and drink. All animal studies were approved by the Medical Laboratory Animal Welfare and Ethics Committee of China Medical University, and the experiments were carried out in accordance with the approved guidelines from the National Institutes of Health for the Care and Use of Laboratory Animals. To establish a cell-derived xenograft animal model, 1 × 10^6^ of engineered 22Rv1 cells were collected, resuspended in Matrigel: RIPM1640 (1:1), and subcutaneously injected (0.2 mL/mouse) into the flanks of athymic mice (n = 6/group). The length, width, and thickness of the tumors were measured with calipers every 5 days. Forty-five days after injection, the mice were humanely sacrificed, and the tumors were collected, weighed, and stored at -80°C for subsequent examination. Tumor tissues were also embedded in paraffin for histological examination. Tumor volumes were calculated using the equation (length × width2)/2. To establish the metastasis animal model, BALB/c nude mice were randomly assigned to two groups (*n* = 5/group) and given an injection of 1 × 10^6^ engineered 22Rv1 cells (control cells or circRNF19A knockdown cells) in 0.1 ml of PBS via the tail vein. Fifty-five days after the injection, the animals were euthanized, and intact lung tissues were isolated for observation and detection. Hematoxylin and eosin staining was applied to the tissue sections. The number of metastatic cancer nests was counted at a magnification of 10X using an inverted microscope (Leica DM3000).

### Statistical analysis

The data are presented as the means ± SEMs from at least three different experiments. Student’s t test and one-way ANOVA were used for statistical analyses with GraphPad Prism 9.0.0 (GraphPad Software, Inc., La Jolla, CA). Analysis of variance (ANOVA) was used to determine the difference between more than two groups of datasets, and two-tailed Student’s t tests were used to determine the difference between two groups of datasets with comparable variance. Significant differences are indicated by the symbols *(*p* < 0.05), **(*p* < 0.01) or ***(*p* < 0.001) for all the statistical analyses. *p* < 0.05 indicated statistical significance.

Additional methods are presented in the Supplementary Methods.

## Results

### Screening of differentially expressed circRNAs in PCa tumors and analysis of the coding ability of DE-circRNAs

**Fig. 1:**
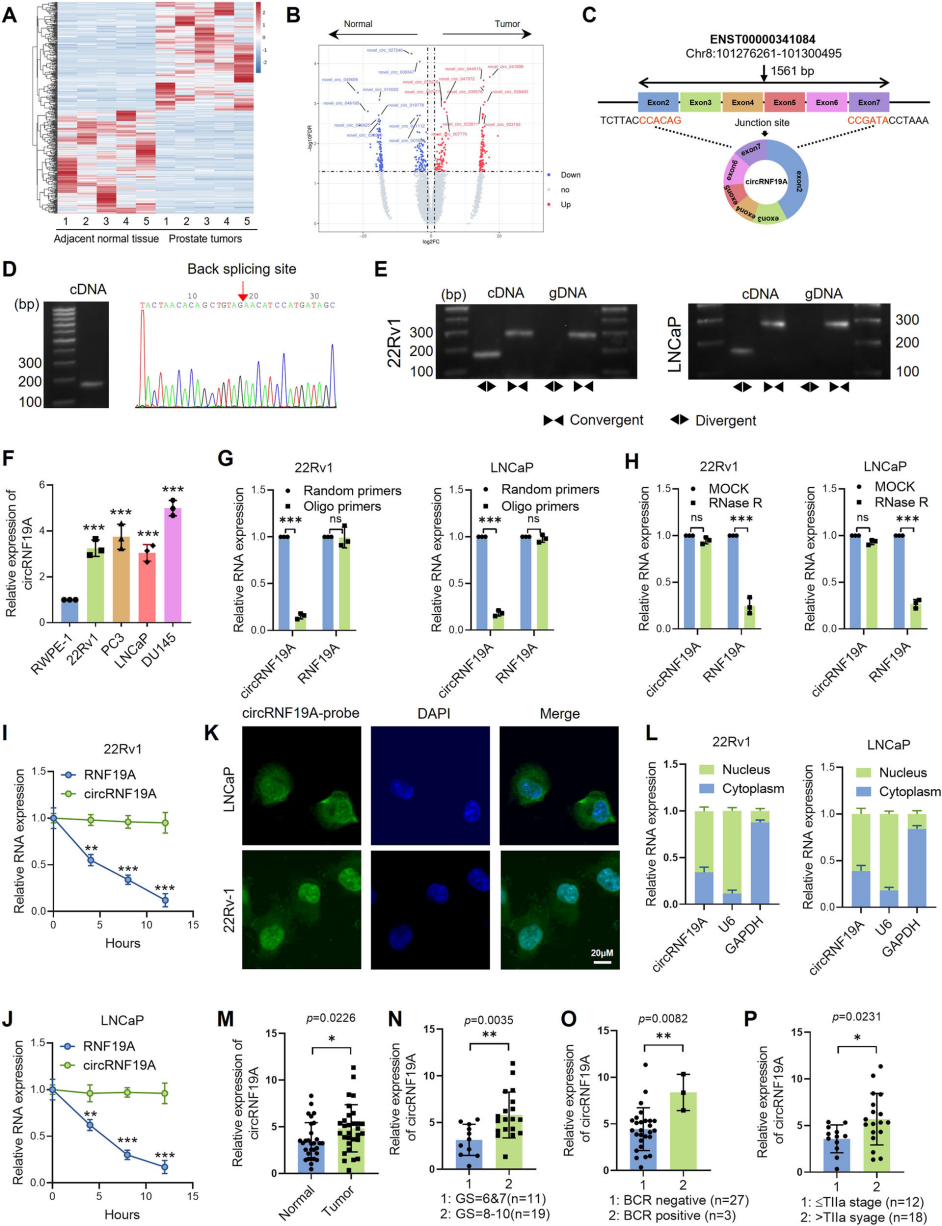
Identification of circRNF19A as an alternative splicing variant of RNF19A in prostate cancer cells. **A** A heatmap showing the differentially expressed circRNAs between five pairs of PCa tumor tissues and adjacent normal prostate tissues. **B** Volcano plots indicating the DE-circRNAs with the top 10 upregulated or downregulated circRNAs highlighted. **C** Schematic illustration of circRNF19A, which is generated by exons 2 to 7 of RNF19A through back-splicing. **D** RT‒PCR analysis of circRNF19A in 22Rv1 cells (left). The sequence of the backsplicing site in circRNF19A identified by Sanger sequencing (right). **E** PCR analysis of circRNF19A amplified by convergent divergent primers in 22Rv1 and LNCaP cells. **F** qRT‒PCR analysis of circRNF19A in one immortalized prostate epithelial cell line and four other PCa cell lines. **G** qRT‒PCR analysis was performed in 22Rv1 cells (left) and LNCaP cells (right) to detect the expression of circRNF19A or linear RNF19A amplified by random primers or oligo primers. **H** qRT‒PCR analysis was performed on 22Rv1 cells (left) and LNCaP cells (right) treated with RNase R for 8 h to detect the expression of circRNF19A or linear RNF19A mRNA. **I**, **J** The half-lives of circRNF19A and linear RNF19A mRNA in 22Rv1 cells (left) and LNCaP cells (right) after RNase R treatment. **K** A nuclear mass separation assay showed that circRNF19A was mostly enriched in the nuclei of 22Rv1 (left) and LNCaP cells (right). **L** FISH further confirmed that circRNF19A was mostly present in the nuclei of 22Rv1 and LNCaP cells (scale bar = 10 μM). **M** circRNF19A was detected in 30 pairs of PCa tumor and matched normal prostate tissue samples. **N** The expression of circRNF19A was detected in PCa tumor samples with low Gleason scores (6 and 7, *n* = 11) or advanced Gleason scores (8–10, *n* = 19). **O** The expression of circRNF19A was detected in PCa tumor samples from patients with biochemical recurrence (*n* = 27) or without biochemical recurrence (*n* = 3). **P** circRNF19A expression was detected in PCa tumor samples from patients with low T stage (≤TIIa stage, *n* = 12) or advanced T stage (>TIIa stage, *n* = 18) disease. *p < 0.05, **p < 0.01, and ***p < 0.001.

To identify the differentially expressed genes (DEGs), a transcriptomics study was performed using PCa tumor tissue and adjacent nontumorous tissue samples from five PCa patients (Fig. S1A). The clinicopathological characteristics of the PCa patients are listed in Table S1. Quality control of the samples and processed data was performed, and the results are shown in Figs. S1 to S2. Genes with a > 2.0-fold change and a *q* value < 0.05 were considered differentially expressed genes (DEGs). Among the DEGs, 423 DE-circRNAs (217 upregulated and 206 downregulated) were identified (Fig. S4A, Table S2). A heatmap of the DE-circRNAs is shown in Fig. 1A. Volcano plots were also constructed to plot the DE-circRNAs (Fig. 1B). Gene Ontology (GO) and Kyoto Encyclopedia of Genes and Genomes (KEGG) analyses were performed to evaluate the biological functions of the parent genes of the DE-circRNAs (Fig. S3A and S3B).

It has been well established that circRNAs may facilitate tumorigenesis in human cancers by encoding specific peptides. Two basic elements are required for circRNAs to be translated into peptides: open reading frames (ORFs) and internal ribosome entry sites (IRESs). The ORF is the foundational framework for translation and is often the first step in translation research. We annotated the ORFs in circRNAs using the cORF pipeline script to identify circRNAs with potential coding capabilities [41]. Since the cirRNAs were generated from linear RNAs, we focused only on the circRNA-specific ORFs that crossed the back-splicing sites of circRNAs. The types of ORFs are shown in Fig. S3C, and the distributions of the lengths of the ORFs are shown in Fig. S3D. An IRES is a nucleic acid sequence, and the presence of an IRES allows RNA to bind directly to the ribosome without relying on the 5′ cap and to be translated directly from the middle of the mRNA. An IRES is essential for circRNA translation. The python package IRESfinder was used to identify IRESs within RNAs [45]. We used IRESfinder to locate the IRES in circRNAs. The IRES scores were calculated and are shown in Fig. S3E and Table S3. Finally, we confirmed 376 circRNAs that possessed both ORFs and IRESs, indicating the potential coding abilities of the circRNAs (Table S4).

### Identification of hsa_circ_0085121 as an alternative splicing variant of RNF19A in prostate cancer cells

To screen for potential protein-coding circRNAs, we analyzed the IRESs and ORFs of all the DE-circRNAs as described above. We focused on large peptides generated from circRNAs. The workflow of the screening for circRNF19A was shown in Fig. S1B. Briefly, significantly upregulated circRNAs in PCa tumors with |log FC| > 2 and coding score > 0.85 were selected, and those circRNAs with the coding potential of micropeptides or rolling translation were further excluded. A set of 28 circRNAs were then screened as candidates. To narrow down the number of genes for further investigation, we aimed to find those circRNAs that might be targeted or be involved in the androgen receptor pathway. Hence, we searched the parental genes of the candidate circRNAs for clues, and the genes correlated with AR (Pearson’s correlation > 0.5, *p* < 0.05) in PCa were listed by analyzing the TCGA_PRAD dataset. We took the intersection of the two gene sets and found that only 18 parental genes crossed between them (Fig. S4C, D). We then searched the 18 circRNAs from the parental genes in circBase and found that 5 out of 18 circRNAs were not documented in public database, and therefore were excluded. Subsequently, we knocked down AR expression in 22Rv1 cells and examined the expression of remaining 13 circRNAs by qRT-PCR analysis. The results showed that only hsa_circ_0085121 (hereby designated as circRNF19A) showed a significantly downregulation after AR knockdown (Fig. S4E), and data from TCGA_PRAD also supported that expression of RNF19A is dramatically correlated with that of AR (Fig. S4F). Our transcriptomic data showed that circRNF19A is significantly upregulated in PCa tumors with the logFC = 3.93 (*p* = 0.02, Fig. S4G). Therefore, we believe that circRNF19A is likely to be highly expressed in PCa tissues and regulated by the androgen receptor, and we decided to choose circRNF19A for further study.

We found that circRNF19A is a novel circRNA that has not been characterized yet. Data from circBase as well as our transcriptome confirmed that circRNF19A is located at chr8:101276261-101300495, with the length of 1561 bp, and has the potential to be translated into a 490 aa protein. The length of the ORF is 1479 bp (starts at 94 and ends at 11) and the IRES is located at 1341–1541. circRNF19A was looped and comprised from exon 2 to exon 7 of RNF19A (Fig. 1C). Two sets of primers were designed to identify circRNF19A in PCa cells. Divergent primers were used to amplify the circular transcripts, and convergent primers were used to identify the linear transcripts. PCR analysis revealed that the divergent primers amplified the predicted PCR products, and Sanger sequencing further confirmed the location of the back-splicing junction (Fig. 1D). Furthermore, circular forms could be amplified from cDNA only using divergent primers but could be amplified from both gDNA and cDNA using convergent primers (Fig. 1E). qRT‒PCR analysis revealed that circRNF19A was abundantly expressed in PCa cell lines (Fig. 1F). Similarly, compared with random reverse transcribed cDNA, oligo reverse transcribed cDNA was unable to amplify circRNF19A, while linear RNF19A could be reverse transcribed from cDNA by both random and oligo primers (Fig. 1G). An RNase degradation assay was subsequently performed to assess the resistance of circRNF19A to RNase. RNase treatment significantly decreased linear RNF19A levels, as shown in Fig. 1H, but circRNF19a was strongly resistant to RNase treatment. To measure the expression levels of circRNF19A and linear RNF19A following RNase treatment, qRT‒PCR was also performed at various time points (Fig. 1I, J). Subsequently, the subcellular location of circRNF19A was determined using FISH analysis (Fig. 1K) and a nuclear mass separation assay (Fig. 1L). Although circRNF19A appeared to be expressed in both the nucleus and cytoplasm of PCa cells, more than 50% of the circRNF19A was located in the nucleus. Finally, we examined the relationship between the expression level of circRNF19A and the clinical progression of prostate cancer. Using qRT‒PCR, we assessed the expression level of circRNF19A in tumor tissues and in paracancerous tissues from 30 PCa patients (Table S5). According to our findings, circRNF19A was highly expressed in prostate cancer tissues (Fig. 1M), and the expression of circRNF19A was strongly correlated with an advanced Gleason score (Fig. 1N), biochemical recurrence (Fig. 1O) and advanced T stage (Fig. 1P). The above results further support the oncogenic role of circRNF19A in prostate cancer, suggesting that it is a potential target for further study.

### circRNF19A knockdown inhibits the proliferation, invasion, migration and docetaxel resistance of PCa cells

**Fig. 2:**
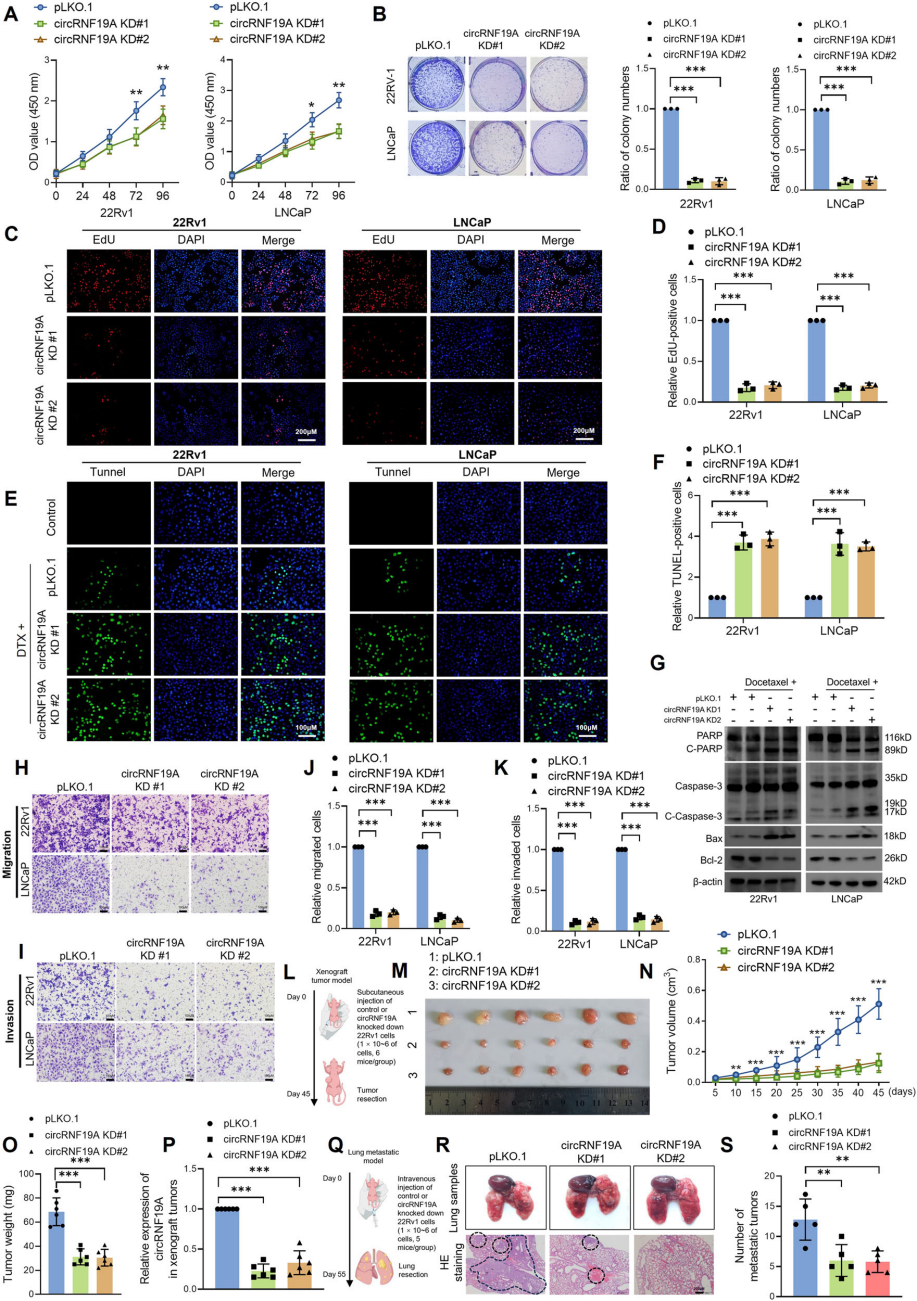
circRNF19A knockdown inhibits the proliferation, invasion, migration and docetaxel resistance of PCa cells. CCK-8 (**A**), colony formation (**B**) and EdU (**C**, **D**) assays were performed in control 22Rv1 and LNCaP cells or in circRNF19A-depleted 22Rv1 and LNCaP cells to measure cell proliferative capacity. Magnification = ×100. **E**, **F** Control 22Rv1 and LNCaP cells or circRNF19A-depleted 22Rv1 and LNCaP cells were treated with DMSO (control group) or 10 nM docetaxel (DTX) for 24 h, after which the cells were subjected to TUNEL staining to assess the degree of apoptosis. Magnification = ×200. **G** In line with the results of the TUNEL staining assay, western blotting was performed for each group of engineered cells to measure the expression of apoptotic markers. **H**, **J** The migration of control, circRNF19A-depleted 22Rv1 and LNCaP cells was measured via a Transwell assay. The images were captured at a magnification of 400×, and the cells were counted using ImageJ software and subjected to statistical analysis. **I**, **K** The invasive ability of control, circRNF19A-depleted 22Rv1-overexpressing and LNCaP cells was measured via a Transwell assay. The images were captured at a magnification of 400×, and the cells were counted using ImageJ software and subjected to statistical analysis. **L** Diagram illustrating the establishment of CDX model. Control or circRNF19A knockdown 22Rv1 cells (1 × 10^6^) were injected into the flanks of the mice (6 mice/group), the experimental mice were observed each five days for monitoring the growth of the xenograft tumors until Forty-five days after injection. **M** Images of the xenograft tumors removed from each group of mice. **N** Growth curve of the xenograft tumors. **O** The weights of the tumors from each group were calculated. **P** qRT‒PCR was performed to measure the expression of circRNF19A in xenograft tumors from each group. **Q** Diagram illustrating the establishment of lung-metastatic model. Control or circRNF19A knockdown 22Rv1 cells (1 × 10^6^) were injected into the tail vein of experimental mice (5 mice/group), and lungs with metastatic nodules were removed fifty-five days after injection. **R** Images of lungs with metastatic nodules removed from the mice (upper panel). The lungs were subjected to hematoxylin-eosin staining, and the metastatic nodules were counted under a microscope (lower panel). The range of the metastatic nests under the microscope is marked by dotted lines. **S** The number of metastatic nodules in each mouse was counted (*n* = 5/group), and the data were statistically analyzed. NS indicates not significant, *p < 0.05, **p < 0.01, and ***p < 0.001.

Given the overexpression pattern of circRNF19A in PCa cells, we assumed that circRNF19A might function as an oncogene in PCa. To investigate the biological functions of circRNF19A, we designed two pairs of shRNAs against circRNF19A and performed a series of experiments to assess the impacts of circRNF19A knockdown (KD) on cell proliferation, apoptosis, invasion and migration. 22Rv1 and LNCaP cells were transduced with two pairs of shRNAs targeting circRNF19A, and the knockdown efficiency was verified by qRT‒PCR analysis (Fig. S5A). The results showed that the expression of circRNF19A was notably decreased after shRNA transduction, while no changes in RNF19A expression were observed. Cell Counting Kit-8 (CCK-8), colony formation and EdU assays were performed to assess the proliferation of 22Rv1 and LNCaP cells. As shown in Fig. 2A–D, circRNF19A KD significantly inhibited the proliferation and survival of 22Rv1 and LNCaP cells. To measure the resistance of PCa cells to apoptosis, docetaxel (DTX) was added to the cells to induce DNA damage and cell apoptosis. Control and circRNF19A KD cells treated with DTX were then subjected to TUNEL staining and western blotting to measure the level of cell apoptosis and the expression of apoptotic markers. As shown in Fig. 2E–G, DTX treatment dramatically induced cell apoptosis and enhanced the expression of apoptotic markers in control cells; in contrast, circRNF19A KD cells exhibited an even greater percentage of apoptotic cells and increased expression of apoptotic markers after DTX stimulation. These results indicated that circRNF19A was critical for maintaining the antiapoptotic capacity of PCa cells. The migration and invasion of PCa cells were also assessed by Transwell assays, and the results suggested that circRNF19A KD significantly decreased cell migration (Fig. 2H, J) and invasion (Fig. 2I, K).

Next, we used in vivo models to investigate the influence of circRNF19A KD on cell proliferation and migration, which are two major attributes of cancer progression. First, a cell-derived xenograft (CDX) model was established by injecting control or circRNF19A KD 22Rv1 cells into the flanks of nude mice (6 mice/group, Fig. 2L). After injection, the growth of the tumors and the condition of the mice were measured and monitored. Forty-five days after injection, the experimental mice bearing xenograft tumors were sacrificed, and the tumors were removed for measurement. Our data showed that circRNF19A KD significantly decreased subcutaneous xenograft tumor growth (Fig. 2M–O) and reduced the expression of circRNF19A (Fig. 2P) in tumors. In addition, control or circRNF19A-KD 22Rv1 cells were injected into nude mice (5 mice/group) through the tail vein to construct a metastatic model (Fig. 2Q). Eight weeks after injection, the mice were sacrificed, and the lungs were removed, observed and subjected to HE staining. The results showed that circRNF19A KD significantly decreased the number of metastatic lung nodes induced by 22Rv1 injection (Figs. 2R, S). Taken together, these findings indicate that circRNF19A functions as an oncogene in PCa by maintaining cell proliferation, invasion, migration and apoptotic resistance.

### circRNF19A encodes a novel 490-aa protein designated circRNF19A-490aa

**Fig. 3:**
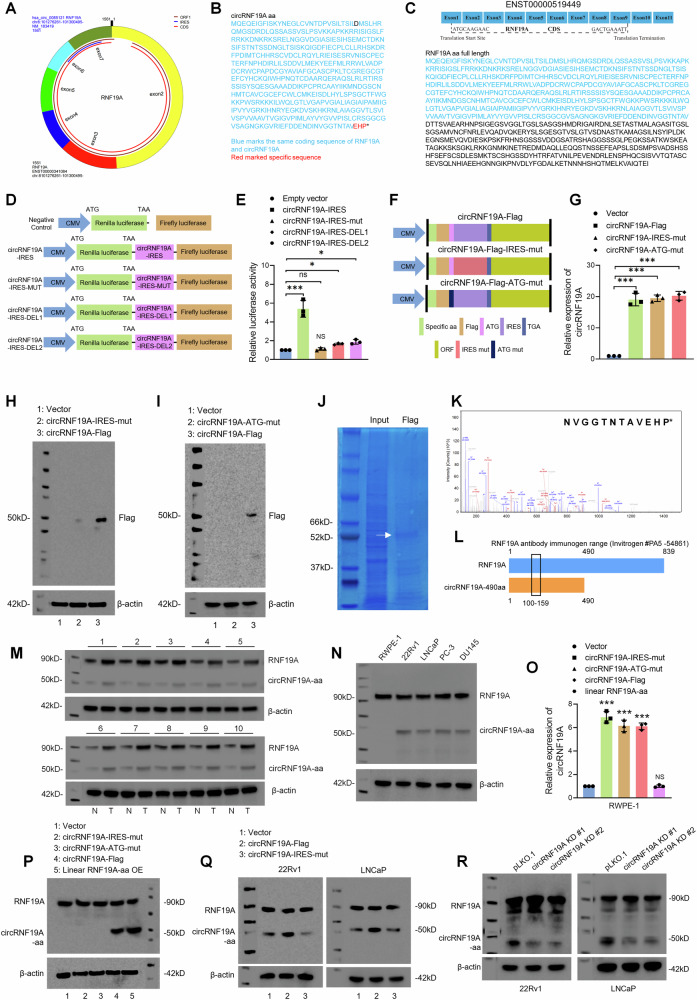
circRNF19A encodes a novel 490-aa protein designated circRNF19A-490aa. **A** Schematic illustration of the IRES and ORF sequences in the circRNF19A gene. **B** The predicted sequence of circRNF19A-aa. Blue: the same coding sequence of circRNF19A and linear RNF19A. Red: the specific sequence of circRNF19A-aa. **C** The full-length sequence of linear RNF19A. **D** Schematic illustration of the empty control construct and luciferase constructs containing the sequences of wild-type circRNF19A-IRES, mutant circRNF19A-IRES, circRNF19A-IRES deletion 1, or circRNF19A-IRES deletion 2. **E** A dual-luciferase reporter assay was performed in 293T cells transfected with empty control, wild-type circRNF19A-IRES, mutant circRNF19A-IRES, circRNF19A-IRES deletion 1, or circRNF19A-IRES deletion 2 luciferase constructs. **F** Schematic illustration of the circRNF19A-Flag, circRNF19A-IRES-mut and circRNF19A-ATG-mut constructs. **G** qRT‒PCR analysis was performed in 293T cells transfected with empty vector, circRNF19A-Flag, the circRNF19A-IRES mutation or the circRNF19A-ATG mutation. **H** 293T cells were transfected with empty vector, circRNF19A-IRES-mut or circRNF19A-Flag, and IP was performed using anti-Flag antibodies to visualize the immune band at approximately 50 kDa after circRNF-Flag transfection. **I** 293T cells were transfected with empty vector, circRNF19A-ATG-mut or circRNF19A-Flag, and IP was performed using anti-Flag antibodies to visualize the 50 kDa band after transfection of circRNF-Flag. **J** Coomassie brilliant blue staining was performed using the protein products of the input or anti-Flag antibody pulldown products from 293T cells transfected with circRNF19A-Flag. The visualized band at approximately 50 kDa was collected and subjected to LC‒MS analysis. **K** The specific sequence of circRNF19A-aa “NVGGTNTAVEHP” was identified via LC‒MS, which suggested that circRNF19A-aa was expressed in circRNF19A-Flag-transfected 293T cells. **L** Schematic structure of the antibody (Invitrogen #PA5-54861) against RNF19A. The immunogen range of the antibody was approximately 100-159 aa, which is an overlapping region of RNF19A and circRNF19A-aa. **M** The expression of RNF19A and circRNF19A-aa in 10 pairs of PCa tumor and matched normal prostate tissue samples was measured via western blotting using anti-RNF19A antibodies (Invitrogen #PA5-54861). **N** The expression of RNF19A and circRNF19A-aa was measured in RWPE-1 cells and four other PCa cell lines by western blotting. **O** qRT‒PCR was performed in RWPE-1 cells transfected with empty vector, control, circRNF19A-IRES-mut, circRNF19A-ATG-mut, circRNF19A-Flag, or linear RNF19A-aa to measure the expression of circRNF19A. **P** The expression of RNF19A and circRNF129A-490aa was examined by western blotting in RWPE-1 cells transfected with empty vector, control, circRNF19A-IRES-mut, circRNF19A-ATG-mut, circRNF19A-Flag, or linear RNF19A-aa. **Q** RNF19A and circRNF129A-490aa expression was examined by western blotting in 22Rv1 and LNCaP cells transfected with empty vector, circRNF19A-Flag, or circRNF19A-IRES-mut. **R** RNF19A and circRNF129A-490aa expression was examined in circRNF19A-depleted 22Rv1 and LNCaP cells by western blotting. NS indicates not significant, *p < 0.05, **p < 0.01, and ***p < 0.001.

By identifying the genetic translation potential of circRNF19A, we next sought to validate our hypothesis in prostate cancer cells. Before that, we predicted the RNA-binding proteins with circRNF19A using circATLAS, RBPsuit and catRAPID software and found that AGO2 was predicted to interact with the upstream flanking of circRNF19A rather than binding to circRNF19 (Figure S5B). We also performed an RIP assay and no significant interaction between circRNF19A and AGO2 protein was found (Fig. S5C), thereby we concluded that circRNF19A may exert oncogenic function through mechanisms other than competitive endogenous RNA (ceRNA). circRNF19A contains an ORF and a segment of the IRES located at 1341 -1541. Our prediction indicated that circRNF19A has the potential to encode a novel protein of 490 aa (Fig. 3A). The predicted sequence of circRNF19A-490aa (hereafter designated circRNF19A-aa) and the sequence of the full-length RNF19A protein are shown in Fig. 3B, C, which demonstrate that the sequence of circRNF19A-aa spans amino acids 1 to 490 of full-length RNF19A, with a unique C-terminus of Glu, His and Pro. To investigate the activity of the IRES in circRNF19A, we constructed luciferase vectors containing the wild-type IRES, mutant IRES, or truncation deletions of the IRES (Fig. 3D and Table S6). We performed a dual-luciferase reporter assay to verify the translation initiation activity of the IRES. The reporter plasmids containing the wild-type IRES exhibited significantly greater luciferase activity than the reporter plasmids containing the negative control, mutation or truncation deletions (Fig. 3E). Next, we created a number of circRNF19A-overexpressing plasmids harboring mutated ATG sequences (circRNF19A-FLAG-ATG-mut) or mutated IRESs (circRNF19A-FLAG-IRES-mut) in a FLAG-tagged vector containing the circRNF19A ORF (circRNF19A-FLAG) to verify the hypothesis that the 490-aa protein is encoded by circRNF19A (Fig. 3F). As shown in Fig. 3G, transfection of these vectors into 293T cells significantly increased the expression of circRNF19A without affecting the level of RNF19A mRNA expression (Fig. S5D), according to the qRT‒PCR results. Western blotting revealed the presence of a 50 kDa FLAG-tagged protein, and FLAG-tag protein expression was eliminated using the circRNF19A-FLAG-IRES-mut or circRNF19A-FLAG-ATG-mut vector (Fig. 3H, I). To further demonstrate that the 50 kDa protein was translated by circRNF19A, liquid chromatography‒tandem mass spectrometry (LC‒MS) was performed to assess the 50 kDa protein products, which were precipitated by anti-FLAG antibodies from circRNF19A-FLAG-transfected 293 T cells. We successfully detected the specific fragment “NVGGTNTAVEHP” of circRNF19A (Fig. 3J, K, Table S7). These findings demonstrated that only circRNF19A-FLAG could be translated into a polypeptide designated circRNF19A-aa.

Since the sequence of circRNF19A-aa spans the 1-490 aa sequence of RNF19A, we used a commercial antibody against RNF19A that targeted the immunogen range shared by circRNF19A-aa and RNF19A (Fig. 3L). Western blotting revealed wild-type RNF19A at ~90 kDa, and an immune band at ~50 kDa was also observed in 10 pairs of PCa tumor samples and adjacent normal prostate tissue samples (Fig. 3M). Next, the expression of RNF19A and circRNF19A-aa was measured in one immortalized normal prostate epithelial cell line, RWPE-1, and four PCa cell lines, 22Rv1, LNCaP, PC-3 and VCaP. Although RNF19A was abundantly expressed in all cell lines, circRNF19A-aa was not expressed in RWPE-1 cells (Fig. 3N). To further confirm that the 50 kDa band was circRNF19A-aa, we transfected circRNF19A-FLAG-IRES-mut, circRNG19A-FLAG-ATG-mut, circRNF19A-FLAG and linear RNF19A-490aa into RWPE-1 cells, and qRT‒PCR assays revealed that circRNF19A was significantly overexpressed in response to transfection with circRNF19A-FLAG-IRES-mut, circRNF19A-FLAG-ATG-mut or circRNF-19A-FLAG, while transfection with linear RNF19A-490aa failed to induce overexpression of circRNF19A (Fig. 3O). Subsequently, western blotting revealed that transfection of either circRNF19A-FLAG or linear RNF19A-490aa induced the expression of the protein at 50 kDa, while transfection of the empty vector, circRNF19A-FLAG-IRES-mut or circRNF19A-FLAG-ATG-mut failed to induce this upregulation. The above results indicated that the overexpression of circRNF19A or linear RNF19A-490aa could enhance the translation of circRNF19A-aa in RWPE-1 cells (Fig. 3P). We also examined the expression of circRNF19A-aa in the AR-positive 22Rv1 and LNCaP cell lines. Western blotting assays showed that transfection of circRNF19A-Flag significantly induced the overexpression of circRNF19A-aa, while transfection of circRNF19A-IRES-mut failed to increase circRNF19A-aa expression. In contrast, no changes in RNF19A expression were detected after transfection with either circRNF19A-Flag or circRNF19A-IRES-mut (Fig. 3Q and S5E and S5F). Finally, we knocked down circRNF19A in 22Rv1 cells and LNCaP cells, and western blotting revealed that the expression of circRNF19A-aa was significantly decreased in accordance with circRNF19A knockdown, while no changes in RNF19A expression were detected (Fig. 3R). Taken together, these findings demonstrate that circRNF19A can be translated into a novel 490 aa polypeptide designated circRNF19A-aa, which is highly expressed in PCa tumor samples.

### The overexpression of circRNF19A-490aa promoted the proliferation, invasion, migration and antiapoptotic capacity of PCa cells

**Fig. 4:**
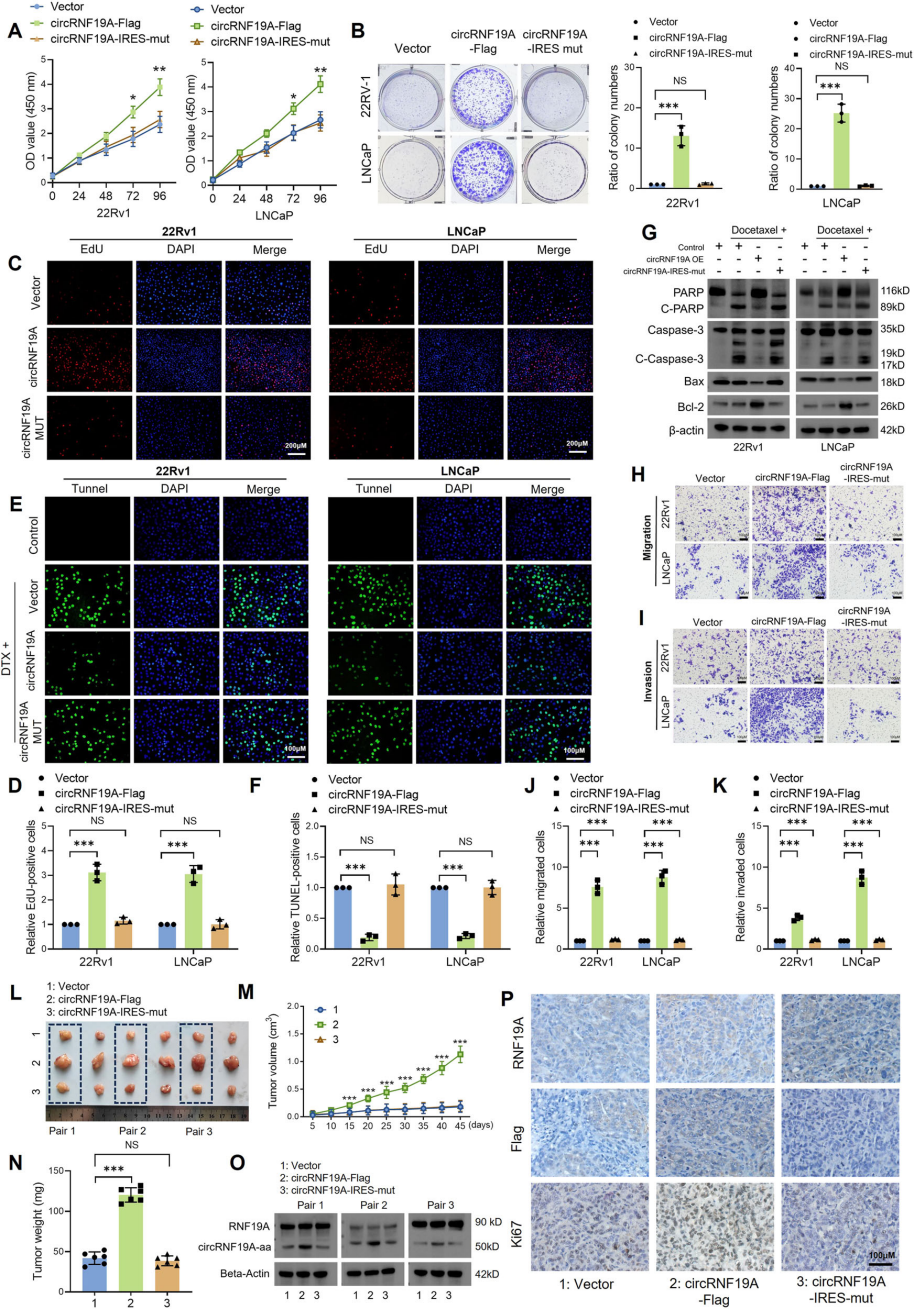
The overexpression of circRNF19A-490aa promoted the proliferation, invasion, migration and antiapoptotic capacity of PCa cells. CCK-8 (**A**), colony formation (**B**), and EdU (**C**, **E**) assays were performed in 22Rv1 and LNCaP cells transfected with empty vector, circRNF19A-Flag, or circRNF19A-IRES-mut. Magnification = ×100. **D**, **F** 22Rv1 and LNCaP cells transfected with empty vector, circRNF19A-Flag, or circRNF19A-IRES-mut were treated with DMSO (control group) or 10 nM docetaxel (DTX) for 24 h, after which the cells were subjected to TUNEL staining to assess the degree of apoptosis. Magnification = ×200. **G** In line with the results of the TUNEL staining assay, western blotting was performed for each group of engineered cells to measure the expression of apoptotic markers. **H**, **J** The migration of 22Rv1 and LNCaP cells transfected with empty vector, circRNF19A-Flag, or circRNF19A-IRES-mut was measured via a Transwell assay. The images were captured at a magnification of ×400, and the cells were counted using ImageJ software and subjected to statistical analysis. **I**, **K** The invasive ability of 22Rv1 and LNCaP cells transfected with empty vector, circRNF19A-Flag, or circRNF19A-IRES-mut was measured via a Transwell assay. The images were captured at a magnification of ×400, and the cells were counted using ImageJ software and subjected to statistical analysis. **L** Xenograft tumors were removed from each group of mice. **M** Growth curve of the xenograft tumors. **N** Xenograft tumors from each group were removed and weighed. **O** Western blotting was performed on xenograft tumors from each pair to measure the expression of RNF19A and circRNF19A-490aa. **P** IHC staining was performed in xenograft tumors to measure the expression levels of RNF19A, Flag (circRNF91A-aa) and Ki67. NS indicates not significant, *p < 0.05, **p < 0.01, and ***p < 0.001.

Next, we investigated the biological functions of circRNF19A-aa in prostate cancer cells. Two cell lines, 22Rv1 and LNCaP, were stably transfected with the empty vector, circRNF19A-Flag or circRNF19A-Flag-IRES-mut. CCK-8 (Fig. 4A), colony formation (Fig. 4B) and EdU (Fig. 4C, E) assays were used to assess the viability and proliferation of PCa cells. In contrast to transfection with circRNF19A-Flag-IRES-mut, which did not result in any changes in cell viability, overexpression of circRNF19A-Flag strongly increased cell viability. Next, the engineered PCa cells were treated with DTX to induce cell apoptosis, and TUNEL staining (Fig. 4D, F) and western blotting (Fig. 4G) were used to measure the number of apoptotic cells and the expression of apoptotic markers, respectively. The findings indicated that DTX treatment significantly increased the rate of cell apoptosis and the expression of apoptotic markers in control PCa cells; in contrast, circRNF19A-overexpressing PCa cells exhibited a significantly decreased level of apoptosis and a decreased expression level of apoptotic markers. Additionally, mutation of the IRES hindered the ability of PCa cells to resist apoptosis induced by circRNF19A overexpression alone. A Transwell assay was used to evaluate the migratory and invasive capabilities of each group of engineered cells. Transfection of circRNF19A-Flag-IRES-mut had no effect on cell invasion or migration, whereas overexpression of circRNF19A-Flag significantly stimulated the migration (Fig. 4H, J) and invasion (Fig. 4I, K) of cells. Subsequently, an in vivo xenograft model was used to assess the effects of circRNF19A-aa on xenograft tumor growth. Following the injection of three groups of engineered cells (six mice/group) into the flanks of BALB/c nude mice, the length and width of the xenograft tumors were measured every five days. Forty-five days after injection, the experimental mice bearing xenograft tumors were sacrificed, and the tumors were excised, weighed, and subjected to IHC staining to detect Ki67 expression. The findings demonstrated that 22Rv1 cells transfected with circRNF19A-Flag significantly promoted in vivo tumor growth (Fig. 4L–N), whereas cells transfected with circRNF19A-Flag-IRES-mut failed to induce any changes in tumor growth. Moreover, the expression of RNF19A and circRNF19A-aa in xenograft tumors was determined by western blotting and IHC staining. Western blotting revealed that the expression of circRNF19A-aa was significantly elevated in tumors derived from circRNF19A-Flag-transfected 22Rv1 cells, while no changes in the expression of circRNF19A-aa were detected in tumors from the other groups (Fig. 4O). IHC staining revealed that Flag was detected in tumors derived from control vector- or circRNF19A-Flag-transfected 22Rv1 cells, whereas no expression of Flag was detected in tumors derived from circRNF19A-IRES-mut-transfected 22Rv1 cells (Fig. 4P). According to the above data, circRNF19A-aa is essential for increasing the proliferative, invasive, migratory, and apoptotic resistance of prostate cancer cells.

### circRNF19A-490aa interacts with HSP90AA1 to regulate AR activity and multiple hallmark tumorigenic pathways

To investigate the underlying mechanisms by which circRNF19A-490aa regulates the malignant behaviors of PCa cells, we performed a co-IP assay using an anti-Flag antibody in 22Rv1 cells transfected with circRNF19A-Flag plasmids (Fig. S5G). LC‒MS analysis revealed a total of 955 proteins in the immunoprecipitated products that potentially interact with circRNF19A-aa, and Kyoto Encyclopedia of Genes and Genomes analysis was performed to evaluate the biological functions of the proteins (Fig. S5H). Among these, a total of 53 proteins with high confidence of interactions (unique peptides > 10, coverage > 35%) were selected (Table S8). Subsequently, we analyzed the data from TCGA_PRAD and screened the protein-coding genes correlated with the disease-specific survival of PCa patients, and a list of 1823 genes were obtained. We found that 10 genes were shared by the two gene sets (Fig. S5I). The biological functions of the 10 genes in tumorigenesis and PCa progression were investigated by literature study, and we chose heat shock protein 90 alpha family class A member 1 (HSP90AA1), enolase 1 (ENO1), dyskerin pseudouridine synthase 1 (DKC1), phosphoglycerate kinase 1 (PGK1) and heterogeneous nuclear ribonucleoprotein F (HNRNPF) for further study. We performed co-IP analysis in 22Rv1 cells using the specific antibody for each candidate protein and found that HSP90AA1 and HNRNPF showed the most significant binding ability with circRNF19A-aa (Fig. S5J). Hereby, HSP90AA1 (Fig. S5K, Table S9) and HNRNPF (Fig. S5L, Table S10) were finally chosen to study.

**Fig. 5:**
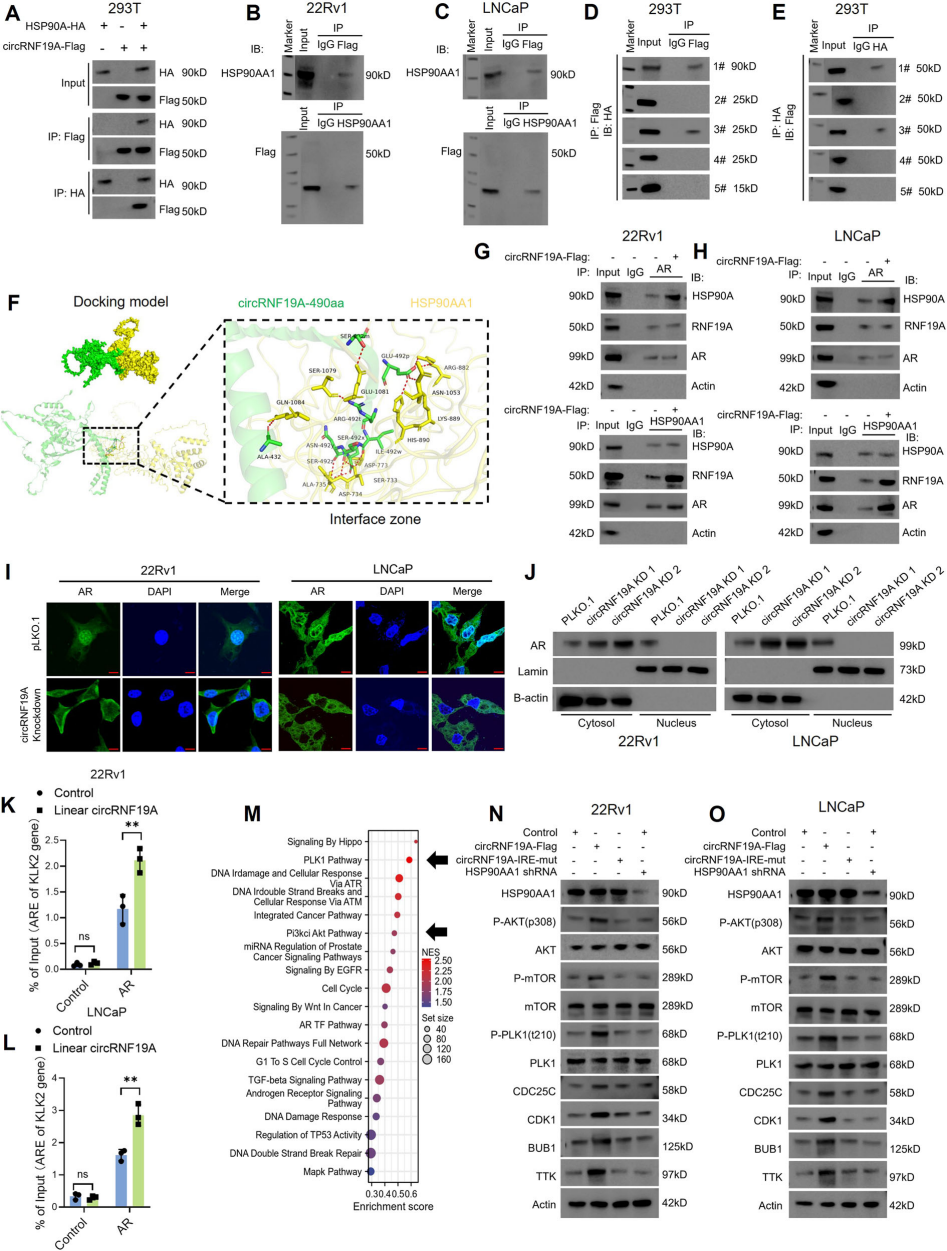
circRNF19A-490aa interacts with HSP90AA1 to regulate AR activity and multiple hallmark tumorigenic pathways. **A** circRNF19A-Flag and HSP90AA1-HA were cotransfected into 293T cells, and the cell lysates were immunoprecipitated with Flag or HSP90AA1 antibodies and immunoblotted with the indicated antibodies. **B**, **C** 22Rv1 and LNCaP cells were immunoprecipitated with Flag or HSP90AA1 antibodies, and the precipitated proteins were subjected to immunoblotting with the indicated antibodies. **D**, **E** circRNF19A-Flag and HSP90AA1-FL or the corresponding truncation construct were cotransfected into 293T cells, the cell lysates were immunoprecipitated with anti-FLAG or anti-HA antibodies, and the immunoprecipitated proteins were subjected to immunoblotting with the indicated antibodies. **F** The docking model and the interface zone between circRNF19A-490aa and HSP90AA1 were predicted and constructed. **G**, **H** circRNF19A-Flag and empty vectors were transfected into the PCa cell lines 22Rv1 and LNCaP. Lysates from control cells or circRNF19A-490aa-overexpressing cells were immunoprecipitated with AR or HSP90AA1 antibodies, and the precipitated proteins were subjected to immunoblotting with the indicated antibodies. **I** Immunofluorescence staining revealed the subcellular translocation of AR (green) in control or circRNF19A-490aa-overexpressing PCa cells. The images were captured by a confocal laser scanning microscope (scale bar = 10 μM). **J** Expression of AR in nuclear and cytosol protein from control PCa cells or circRNF19A-depleted PCa cells was measured by western blotting. Lamin was used as an internal reference for nuclear protein. **K**, **L** CircRNF19A-490 overexpression increased the enrichment of ARs in the ARE region of the PSA gene. 22Rv1 and LNCaP cells (with control or circRNF19A-490aa overexpression) were analyzed via ChIP assays using control or AR antibodies. Precipitated chromatin was analyzed by qRT‒PCR using primers for the ARE region of the PSA gene. The data were calculated as a percentage of the input. **M** Gene set enrichment analysis was performed using genes that were correlated with HSP90AA1 in PCa tumors, and the indicated enrichment pathways are shown in the bubble plot. The arrows highlight the gene sets of PID_PLK1_PATHWAY (upper) and PID_PI3KCI_AKT_PATHWAY (lower). **N**, **O** Western blotting was performed to measure the expression of Akt/phosphorylated-Akt, mTOR/phosphorylated-mTOR, PLK1/phosphorylated PLK1 and downstream factors of the PLK1 pathway, namely, CDC35C, CDK1, BUB1 and TTK, in 22Rv1 and LNCaP cells (control cells, circRNF19A-Flag-transfected cells, circRNF19A-IRES-mut-transfected cells, and circRNF19A-Flag-transfected cells with HSP90AA1 knockdown). NS indicates not significant, *p < 0.05, **p < 0.01, and ***p < 0.001.

HSP90 is an ATP-dependent chaperone that forms a chaperone complex with HSP70 to regulate the stability of its client proteins [22]. In PCa, HSP90 interacts with AR-FL or AR-V7, sustains the stability and activity of AR [46], and has been identified as a promising therapeutic target for PCa treatment [31]. We constructed HA-tagged HSP90AA1-overexpressing plasmids, and HEK293T cells were cotransfected with HSP90AA1-HA and circRNF19A-flag. As shown in Fig. 5A, a co-IP assay confirmed the interaction between HSP90AA1 and circRNF19A-490aa. Next, we performed co-IP assays in 22Rv1 and LNCaP cells. The cell lysates were subjected to immunoprecipitation with control IgG, HSP90AA1 or Flag antibodies, and the precipitated proteins were blotted with antibodies against HSP90AA1 or Flag. The results further confirmed that HSP90AA1 interacts with circRNF19A-490aa in PCa cells (Fig. 5B and C). Next, to identify the binding regions of HSP90AA1, we constructed HA-tagged truncations of HSP90AA1 (Fig. S6A), and these truncation mutants of HSP90AA1 were cotransfected with circRNF19A-Flag into 293T cells. Cell lysates were immunoprecipitated with control IgG, HA or Flag antibodies, and the precipitated proteins were immunoblotted for detection with HA or Flag antibodies. The results indicated that circRNF19A-490aa interacts with the 201-400 aa region of HSP90AA1 (Fig. 5D, E). To simulate the interaction between circRNF19A-aa and HSP90AA1, the HDOCK web server (http://hdock.phys.hust.edu.cn/) was utilized for in silico molecular docking analysis (Fig. 5F). The secondary structure of circRNA19A-aa was predicted and modeled by AlphaFold2 (https://alphafold.com/), and the secondary structure of HSP90AA1 was also modeled by AlphaFold2. The interaction between circRNF19A-aa and HSP90AA1 was visualized by PyMOL (https://pymol.org/).

HSP90AA1 acts as a chaperone for AR in AR activation. Having identified the interaction between circRNF19A-490aa and HSP90AA1, we next sought to determine whether circRNF19A-490aa affects the binding between HSP90AA1 and AR. circRNF19A-490aa was overexpressed in PCa cells by transfection of linear RNF19A-490aa plasmids, and lysates from control or circRNF19A-490aa-overexpressing cells were subjected to immunoprecipitation with control IgG or AR or HSP90AA1 antibodies. Co-IP assays revealed that overexpression of circRNF19A-490aa significantly enhanced the interaction between HSP90AA1 and AR in PCa cells (Fig. 5G, H). IF staining (Fig. 5I) revealed that knockdown of circRNF19A induced the subcellular translocation of AR from the nucleus to the cytoplasm. Western blotting assays demonstrated that the knockdown of circRNF19A markedly suppressed the expression of AR in nuclear proteins, while simultaneously promoting its expression in cytosol proteins (Fig. 5J). Chromatin immunoprecipitation (ChIP) assays also showed that overexpression of circRNF19A-490aa significantly enhanced the recruitment of ARs to the AREs of AR target genes (Fig. 5K). qRT‒PCR analysis further showed that the expression of AR target genes was upregulated in response to circRNF19A-490aa overexpression (Fig. S6B and S6C). These results indicated that the interaction between circRNF19A-490aa and HSP90AA1 was critical for maintaining the nuclear localization of AR and activating the AR pathway. To further explore whether circRNF19A-490aa modulates the malignancy of PCa cells through other HSP90AA1-involved pathways, we performed GSEA using genes related to the relative expression of HSP90AA1 (|logFC| >1.5), and gene expression profiles were obtained from TCGA datasets (Fig. 5L). The results demonstrated that HSP90AA1 was enriched in cancer pathways such as the Hippo pathway, PLK1 pathway, PI3K/AKT pathway and WNT pathway (Fig. 5M). In addition, HSP90AA1 was found to be related to the DNA damage response and the regulation of TP53 and the cell cycle. We determined the expression of phosphorylated AKT/mTOR and PLK1, as well as downstream genes of the PLK1 pathway, such as CDC25C, CDK1, BUB1 and TTK, using western blotting. Our data revealed that overexpression of circRNF19A significantly enhanced the activity of the AKT/mTOR and PLK1 pathways, whereas mutation of the IRES or knockdown of HSP90AA1 notably inhibited the activation of the AKT/mTOR and PLK1 pathways induced by circRNF19A overexpression (Fig. 5N, O). Collectively, these results show that circRNF19A-490aa facilitates the activation of the AKT/mTOR and PLK1 pathways by interacting with HSP90AA1 in PCa cells.

### circRNF19A-490aa depends on HSP90AA1 to facilitate the malignant phenotypes of PCa cells

**Fig. 6:**
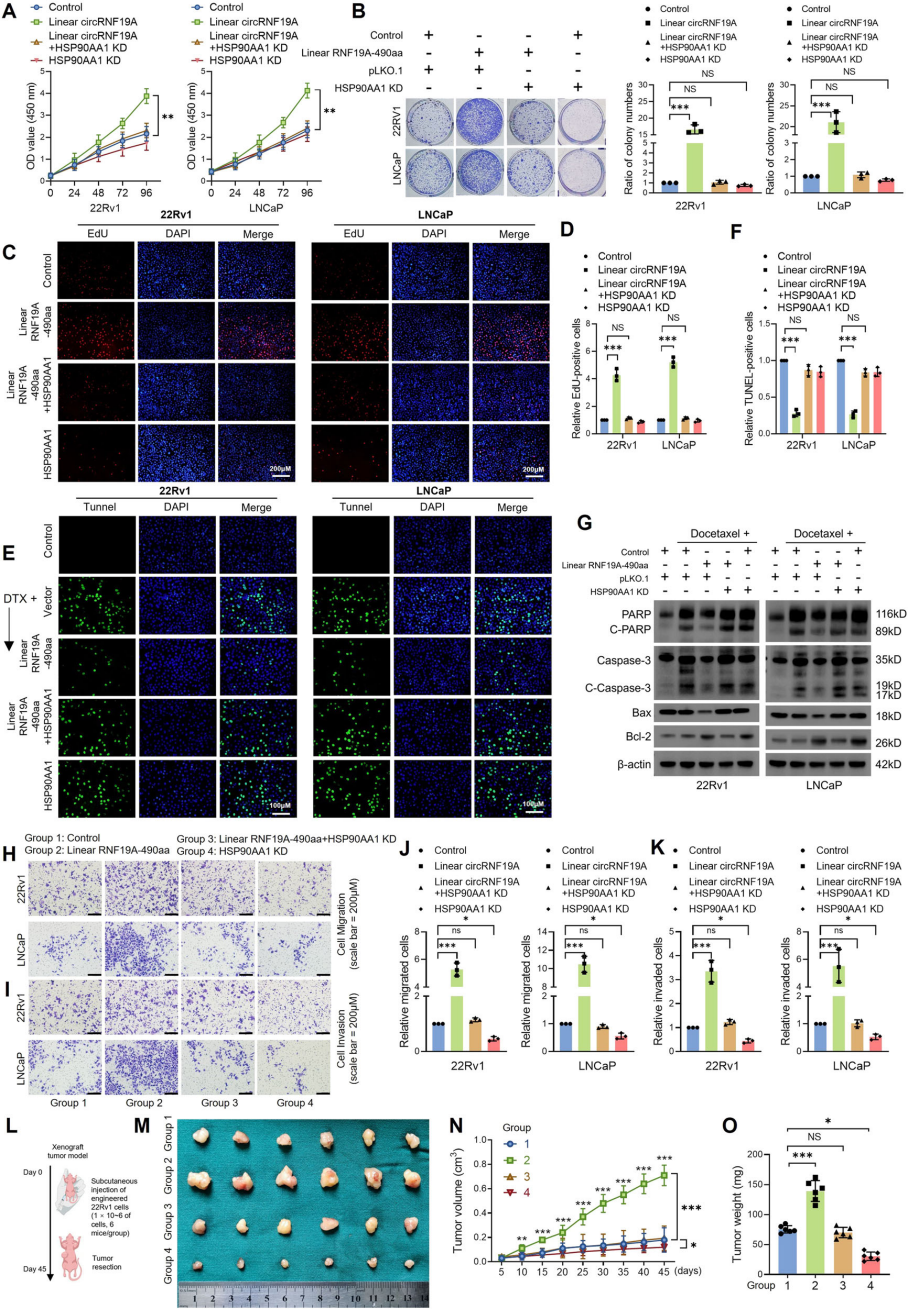
circRNF19A-490aa depends on HSP90AA1 to facilitate the malignant phenotypes of PCa cells. **A** CCK-8 assays were performed in control cells, cells stably overexpressing linear RNF19A-490aa, cells stably overexpressing linear RNF19A-490aa, cells with HSP90AA1 knockdown, and cells with HSP90AA1 knockdown. **B** A colony formation assay was performed in line with the CCK8 assay to assess cell proliferation and survival in control cells, cells stably overexpressing linear RNF19A-490aa, cells stably overexpressing linear RNF19A-490aa with HSP90AA1 knockdown, and cells with HSP90AA1 knockdown. **C**, **D** An EdU incorporation assay was performed in control cells, cells stably overexpressing linear RNF19A-490aa, cells stably overexpressing linear RNF19A-490aa, cells with HSP90AA1 knockdown, and cells with HSP90AA1 knockdown. Magnification = ×100. **E**, **F** A TUNEL staining assay was performed to examine cell apoptosis in control cells, cells stably overexpressing linear RNF19A-490aa, cells stably overexpressing linear RNF19A-490aa with HSP90AA1 knockdown, and cells in which HSP90AA1 was knocked down. The engineered 22Rv1 and LNCaP cells were pretreated with DMSO (control group) or 10 nM docetaxel (DTX) for 24 h. Magnification = ×200. **G** In line with the results of the TUNEL staining assay, western blotting was performed for each group of engineered cells to measure the expression of apoptotic markers. **H**, **J** The migratory ability of control cells, linear RNF19A-490aa stably overexpressing cells, linear RNF19A-490aa stably overexpressing cells with HSP90AA1 knockdown, or HSP90AA1 knockdown cells was measured via a Transwell assay. The images were captured at a magnification of 400×, and the cells were counted using ImageJ software and subjected to statistical analysis. **I**, **K** The invasive ability of control cells, linear RNF19A-490 aa stably overexpressing cells, linear RNF19A-490aa stably overexpressing cells with HSP90AA1 knockdown, or HSP90AA1 knockdown cells was measured via a Transwell assay. The images were captured at a magnification of 400×, and the cells were counted using ImageJ software and subjected to statistical analysis. **L** Diagram illustrating the establishment of CDX model. 1 × 10^6^ of Engineered 22Rv1 cells (Control cells, linear RNF19A-490aa overexpressed cells, linear RNF19A-490aa overexpressed with HSP90AA1 knocked down cells, HSP90AA1 knocked down cells) were injected into the flanks of the mice (6 mice/group), the experimental mice were observed each five days for monitoring the growth of the xenograft tumors until Forty-five days after injection. **M** Images of xenograft tumors removed from each group of mice. **N** The volume of subcutaneous xenograft tumors. The volumes of the tumors were measured five days after injection. **O** The weight of subcutaneous xenograft tumors from each group. *p < 0.05, **p < 0.01, and ***p < 0.001.

To verify whether circRNF19A-aa modulates the malignant phenotypes of PCa cells by interacting with HSP90AA1, we constructed four different types of engineered PCa cells: control cells, linear RNF19A-490aa-overexpressing cells, HSP90AA1-knockdown cells, and linear RNF19A-490aa-overexpressing cells with simultaneous HSP90AA1 knockdown. Western blotting was used to assess the expression of RNF19A, circRNF19A-aa, and HSP90AA1 in each group of cells (Fig. S6D). The results of the cell proliferation assay, including the CCK-8 assay (Fig. 6A), colony formation assay (Fig. 6B) and EdU staining (Fig. 6C, D), demonstrated that the overexpression of circRNF19A-aa notably enhanced the proliferation of PCa cells in vitro. Conversely, the knockdown of HSP90AA1 in cells that stably overexpress circRNF19A-aa resulted in a partial inhibition of the cell proliferation enhanced by circRNF19A-aa overexpression alone. TUNEL staining (Figs. 6E, F) and western blotting (Fig. 6G) revealed that overexpression of circRNF19A-aa promoted cell resistance to DTX stimulation, while depletion of HSP90AA1 in circRNF19A-aa overexpressed cells partially hampered the enhanced resistance to DTX. Moreover, the migratory (Fig. 6H, J) and invasive capacities (Fig. 6I, K) of PCa cells were enhanced in response to circRNF19A-aa overexpression, while knockdown of HSP90AA1 in cells stably overexpressing circRNF19A-aa partially inhibited the migration and invasion of PCa cells induced by circRNF19A-aa overexpression alone. Furthermore, HSP90AA1 knockdown alone resulted in a notable reduction in cell migration and invasion compared to the control group. The xenograft model experiments demonstrated that circRNF19A-aa overexpression significantly enhanced the growth of xenograft tumors. Conversely, the knockdown of HSP90AA1 in circRNF19A-aa-overexpressed 22Rv1 cells exhibited a partial inhibitory effect on tumor growth, while the knockdown of HSP90AA1 alone resulted in a marked reduction in tumor proliferation (Fig. 6L–O). Western blotting was used to measure the expression of RNF19A, circRNF19A-aa and HSP90AA1 in xenograft tumors, and circRNF19A-aa was found to be significantly upregulated in tumors derived from linear RNF19A-490aa-transfected cells, whereas the expression of HSP90AA1 was found to be dramatically decreased in tumors derived from HSP90AA1-knockdown cells. (Fig. 6O). Our data suggest that HSP90AA1 is essential for maintaining the circRNF19A-aa-enhanced malignant phenotype of PCa cells.

### circRNF19A-490aa interacts with HNRNPF and induces the CRPC phenotype in PCa cells

**Fig. 7:**
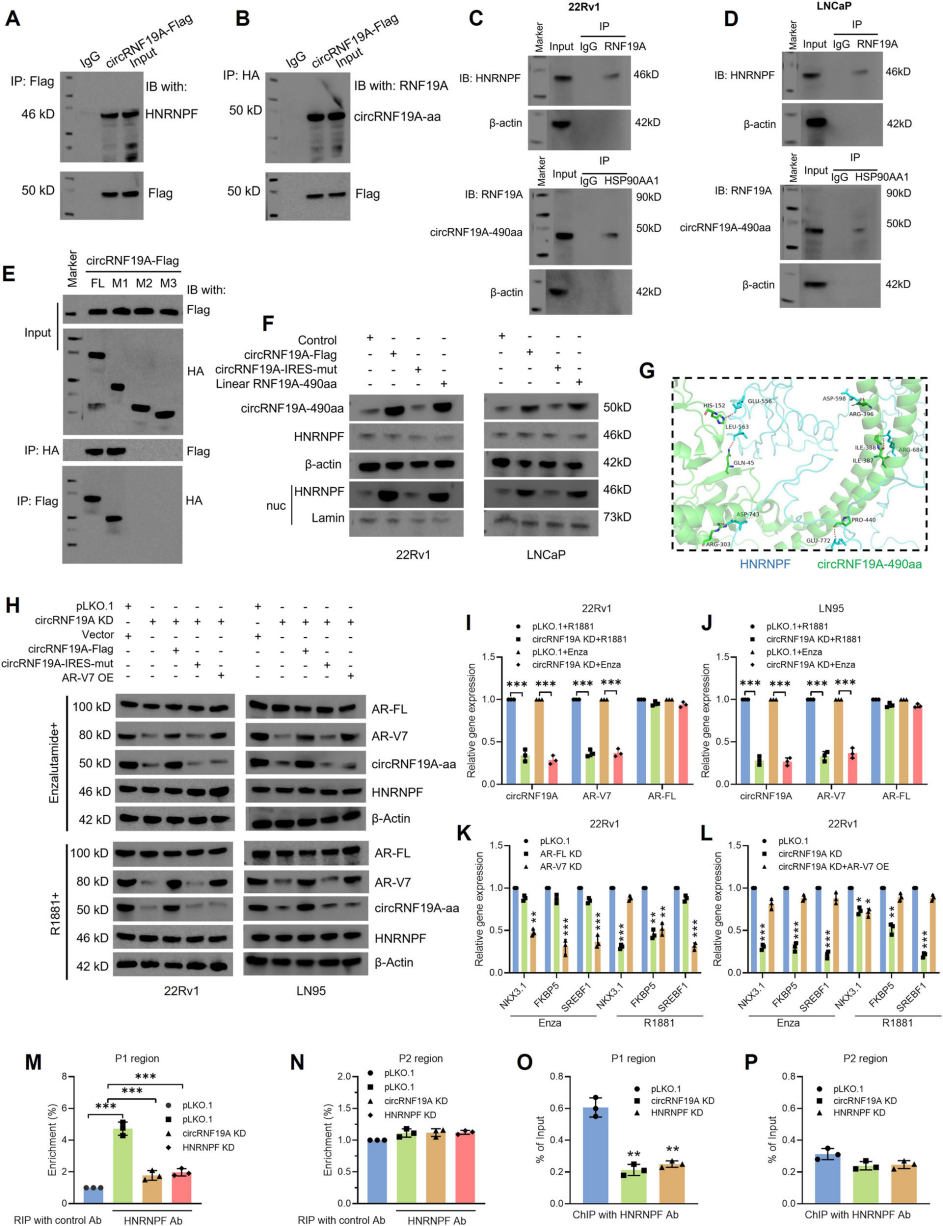
circRNF19A-aa interacts with HNRNPF and recruits it to the splicing site of AR-V7, thereby enhances its alternative splicing and induces the CRPC phenotype in PCa cells. **A**, **B** Lysates from 293 T cells transfected with circRNF19A-Flag or HNRNPF-HA were immunoprecipitated with Flag or HA antibodies, and the precipitated proteins were immunoblotted with the indicated antibodies. **C**, **D** Lysates from 22Rv1 or LNCaP cells transfected with circRNF19A-Flag or HNRNPF-HA were immunoprecipitated with Flag or HA antibodies, and the precipitated proteins were immunoblotted with the indicated antibodies. **E** Cell lysates from 293T cells transfected with HNRNPF FL or M1, M2, or M3 truncation of HNRNPF were immunoprecipitated with HA or Flag antibodies, and the precipitated products were immunoblotted with the indicated antibodies. **F** Western blotting was performed on total proteins or nuclear proteins from 22Rv1 and LNCaP cells transfected with empty vector, circRNF19A-Flag, circRNF19A-IRES-mut, or linear RNF19A-490aa to detect the expression of circRNF19A-490aa, HNRNPF, and nuclear HNRNPF. **G** The docking model and interface zone were predicted and constructed. **H** Western blotting was performed in control 22Rv1 and LN95 cells or circRNF19A-depleted 22Rv1 and LN95 cells transfected with empty vector, circRNF19A-Flag, circRNF19A-IRES-mut or AR-V7 overexpression plasmids under androgen-deprived and normal androgen conditions to measure the expression of the indicated proteins. **I**, **J** qRT‒PCR analysis was performed in control or circRNF19A-depleted 22Rv1 or LN95 cells under androgen-deprived and normal androgen conditions to measure the expression of AR/AR-V7 target genes. **K** qRT‒PCR analysis was performed in control 22Rv1 cells, AR-FL-depleted 22Rv1 cells, and AR-V7-depleted 22Rv1 cells under androgen-deprived and normal androgen conditions to measure the expression of AR/AR-V7 target genes. **L** qRT‒PCR analysis of control, circRNF19A-depleted and circRNF19A-depleted AR-V7-overexpressing 22Rv1 cells under androgen-deprived and normal androgen conditions was performed to examine the expression of AR/AR-V7 target genes. **M**, **N** 22Rv1 cells (control, circRNF19A KD, or HNRNPF KD) were subjected to RIP assays using HNRNPF antibodies. Precipitated RNAs were isolated and analyzed via qRT‒PCR with primers that amplified the P1 or P2 region. The data were calculated as the percentage of input and plotted as the fold change over the control. **O**, **P** 22Rv1 cells (control, circRNF19A KD, or HNRNPF KD) were subjected to ChIP assays using HNRNPF antibodies. Precipitated chromatin fragments were isolated and analyzed via qPCR with primers that amplified the P1 or P2 region. The data were calculated as the percentage of input. NS indicates not significant, *p < 0.05, **p < 0.01, and ***p < 0.001.

We then sought to investigate the effects of circRNF19A-aa-HNRNPF interaction in PCa cells. Previously, we have shown that HNRNPF binds to the AR-V7 splice site and induces the generation of AR-V7 [21]. Therefore, we next sought to verify the interactions between circRNF19A-aa and HNRNPF in PCa cells. We constructed HA-tagged HNRNPF overexpression plasmids and cotransfected them with circRNF19A-Flag into 293T cells. Immunoprecipitation with HA or Flag antibodies confirmed that HNRNPF interacts with circRNF19A-aa in 293T cells (Fig. 7A, B). Next, the interaction between endogenous HNRNPF and circRNF19A-aa was examined in 22Rv1 and LNCaP cells. The cell lysates were immunoprecipitated with IgG, RNF19A or HNRNPF antibodies, and the precipitated proteins were immunoblotted with HNRNPF or RNF19A antibodies. The results showed that HNRNF interacts with circRNF19A-aa but not with RNF19A in PCa cells (Fig. 7C, D). We detected HNRNPF expression in control and circRNF19A KD 22Rv1 cells; however, no changes in HNRNPF expression were observed in either group of cells, indicating that circRNF19A does not affect HNRNPF expression (Fig. S6E). To investigate the binding domain of HNRNPF, we constructed HNRNPF truncation mutants (Fig. S6F), and HA-tagged full-length (FL) HNRNPF, M1 (0-300 aa), M2 (0-200 aa) and M3 (0-100 aa) HNRNPF were cotransfected with circRNF19A-Flag into 293T cells. Co-IP was performed using anti-HA or anti-Flag antibodies, and anti-HA or anti-Flag antibodies were used for immunoblotting. The results suggested that circRNF19A-aa interacts with FL and M1 of HNRNPF but not with M2 or M3 of HNRNPF, suggesting that circRNF19A-aa interacts with the zinc finger RNPHF (zf-RNPHF) domain of HNRNPF (Fig. 7E). To investigate whether circRNF19A affects the subcellular localization of HNRNPF, we tested the expression of HNRNPF in total protein and nuclear protein samples from PCa cells transfected with control vectors, circRNF19A-Flag, circRNF19A-IRES-mut or linear RNF19A-490aa. Western blotting revealed that overexpression of circRNF19A-aa did not change the total protein expression of HNRNPF but did significantly increase the nuclear protein expression of HNRNPF (Fig. 7F), indicating that circRNF19A-aa was able to enhance the nuclear localization of HNRNPF. To simulate the interaction between circRNF19A-aa and HNRNPF, the HDOCK web server (http://hdock.phys.hust.edu.cn/) was used for in silico molecular docking analysis. The secondary structure of HNRNPF was modeled by AlphaFold2 (https://alphafold.com/). The interaction between circRNF19A-aa and HNRNPF was visualized by PyMOL (https://pymol.org/) (Fig. 7G and S6G).

We then analyzed the correlation between the expression of HNRNPF and PCa progression using data from the TCGA. We found that HNRNPF was significantly overexpressed in PCa tumors (Fig. S6H and S6I) and that high HNRNPF expression was correlated with advanced T stage (Fig. S6J), PSA level (Fig. S6K) and Gleason score (Figure S6L) in PCa patients. Moreover, high HNRNPF expression correlated with unfavorable overall survival (Figure S6M) and disease-specific survival (Fig. S6N) in PCa patients. HNRNPF expression was detected in ten pairs of PCa tumor tissues and matched normal tissues, and the results showed a dramatic increase in HNRNPF expression in PCa tumors (Fig. S6O). These results indicated an oncogenic role of HNRNPF in PCa. We then examined the impacts of HNRNPF KD on the proliferation and apoptotic resistance of PCa cells by performing CCK-8, colony formation, TUNEL and annexin V-FITC/PI assays. PCa cells were transduced with two pairs of shRNAs to knock down HNRNPF, and the knockdown efficiency was confirmed by qRT‒PCR and western blotting (Fig. S6P and S6Q). We found that cell proliferation (Fig. S7A–D) and apoptotic resistance (Fig. S7E–G) were notably attenuated after HNRNPF KD. In addition, HNRNPF KD significantly increased the sensitivity of enzalutamide-resistant LNCaP cells to enzalutamide treatment (Fig. S7H), indicating that HNRNPF was crucial for maintaining the CRPC phenotype of PCa cells. Moreover, overexpression of circRNF19A-aa strongly enhanced cell resistance to enzalutamide (Fig. S7I). Overall, we concluded that circRNF19A-aa mutually interacts with the zf-RNPHF domain of HNRNPF and collaborates with HNRNPF to enhance the castration-resistant phenotype of PCa cells.

### circRNF19A-aa recruits HNRNPF to the splicing site of AR-V7 and enhances its alternative splicing

As we confirmed that circRNF19A-aa interacts with HNRNPF in PCa cells, we next sought to investigate whether circRNF19A-aa acts as a coactivator to enhance the AS of AR-V7, which is driven by HNRNPF. PCa cells were treated with enzalutamide or R1881 to simulate androgen deprivation conditions or normal androgen conditions. circRNF19A was knocked down in 22Rv1 and LN95 cells under androgen deprivation conditions or normal androgen conditions, and western blotting revealed that AR-V7 expression was notably decreased after circRNF19A depletion, while no changes in the expression of AR or HNRNPF were detected (Fig. 7H). We restored the expression of circRNF19A by transfecting cells with circRNF19A-Flag or circRNF19A-IRES-mut. The results suggested that the AR-V7 level was subsequently restored after transfection with circRNF19A-Flag but not after transfection with circRNF19A-IRES-mut, indicating that the circRNF19A-aa isoform regulates AR-V7 expression. Finally, AR-V7 expression was restored by AR-V7 overexpression. Next, the expression of circRNF19A, AR-FL and AR-V7 in 22Rv1 and LN95 cells was assessed by qRT‒PCR. circRNF19A KD decreased the level of AR-V7 under both conditions but did not affect the AR expression level (Fig. 7I and J). Three well-characterized AR target genes, NKX3.1, FKBP5 and SREBF1, were used to measure the activity of AR and AR-V7. Among these genes, NKX3.1 depends on AR-FL under normal androgen conditions but becomes dependent on AR-V7 under androgen-deprived conditions, FKBP5 depends on both AR and AR-V7 under normal androgen conditions but depends mainly on AR-V7 under androgen-deprived conditions, and SREBF1 depends only on AR-V7 under both conditions. shRNAs targeting the AR LBD (Fig. S8A) or the AR-V7 3’ UTR (Fig. S8B) were used to knock down AR or AR-V7, respectively. Western blotting revealed that shRNA targeting AR or AR-V7 significantly decreased the expression of AR or AR-V7 under androgen deprivation conditions and normal androgen conditions. Consistent with the western blotting results, the qRT‒PCR results showed that under androgen-deprived conditions, the expression of NKX3.1, FKBP5 and SREBF1 was notably decreased in response to AR-V7 KD but not in response to AR KD in 22Rv1 cells (Fig. 7K). Under normal androgen conditions, NKX3.1 expression was downregulated only after AR KD, FKBP1 expression was downregulated significantly after AR KD and partially after AR-V7 KD, while SREBF1 expression decreased only in response to AR-V7 KD. These results were consistent with the dependence of the AR/AR-V7 target genes under either condition in PCa cells. Subsequently, we knocked down circRNF19A and reoverexpressing AR-V7 in circRNF19A-KD cells under both conditions (Fig. S8C,D). qRT‒PCR revealed that under androgen deprivation conditions, NKX3.1, FKBP1 and SREBF1 were downregulated in circRNF19A KD cells, and their expression was restored by reoverexpression of AR-V7 (Fig. 7L). Notably, under normal androgen conditions, NKX3.1 expression was downregulated in circRNF19A KD cells but no changes in NKX3.1 expression were observed in AR-V7 reoverexpressing cells (as circRNF19A KD also inhibited the activity of AR-FL), FKBP5 expression was downregulated after circRNF19A KD and recovered after AR-V7 reoverexpression, and SREBF1 expression was most strongly altered after circRNF19A KD and AR-V7 reoverexpression. These results suggest that the depletion of circRNF19A leads to the loss of AR-V7 under androgen deprivation conditions and normal androgen conditions.

To investigate whether circRNF19A promotes the splicing of AR-V7, we used an AR-V7 minigene reporter, which was described in our previous study [21]. As the splicing of AR-FL mRNA occurs via the 3’ splice site in front of exon 4, the splicing of AR-V7 involves the use of an alternative 3’ splice site in front of exon 3b. In the AR-V7 minigene reporter, exon 3b and the flanking ∼400-bp nucleotide sequence were inserted between exons 3 and 4 of the AR gene, as shown in Fig. S8E. The plasmids were transfected into AR-negative PC-3 cells, which were subsequently transduced with the negative control or circRNF19A shRNA. qRT‒PCR analysis was performed to detect the splicing products reflecting AR-V7 splicing or AR-FL splicing using specific primers. circRNF19A KD and HNRNPF KD significantly reduced AR-V7 splicing but had no effect on AR-FL splicing (Fig. S8F,G). To address whether circRNF19A-aa recruits HNRNPF to the splicing site of AR-V7, RIP and ChIP assays were performed. Two sets of primers (Fig. S8H) were designed to target the splicing site of AR-V7 (the P1 region, the 3’ splicing site of exon 3b) and AR-FL (the P2 region, the 3’ splicing site of exon 4). The results of the RIP assay showed that HNRNPF was enriched in the P1 region but not in the P2 region, while KD of either HNRNPF or circRNF19A strongly decreased the enrichment of HNRNPF in the P1 region (Fig. 7M, N). We next performed a ChIP assay to further determine whether circRNF19A-aa recruits HNRNPF to exon 3b of AR genomic DNA. The chromatin fragments were precipitated using HNRNPF antibodies, and the DNA fragments of the P1 and P2 regions were detected using the same set of primers used in the RIP assay (Fig. S8I). Consistently, ChIP assays showed that HNRNPF was enriched in the P1 region but not in the P2 region, and KD of either circRNF19A or HNRNPF strongly decreased the enrichment of HNRNPF in the P1 region (Fig. 7O, P). Taken together, these results indicate that circRNF19A-aa recruits HNRNPF to the splicing site of AR-V7 and enhances its alternative splicing.

### AR regulates the expression of circRNF19A and circRNF19A-490aa through binding to the promoter of RNF19A

**Fig. 8:**
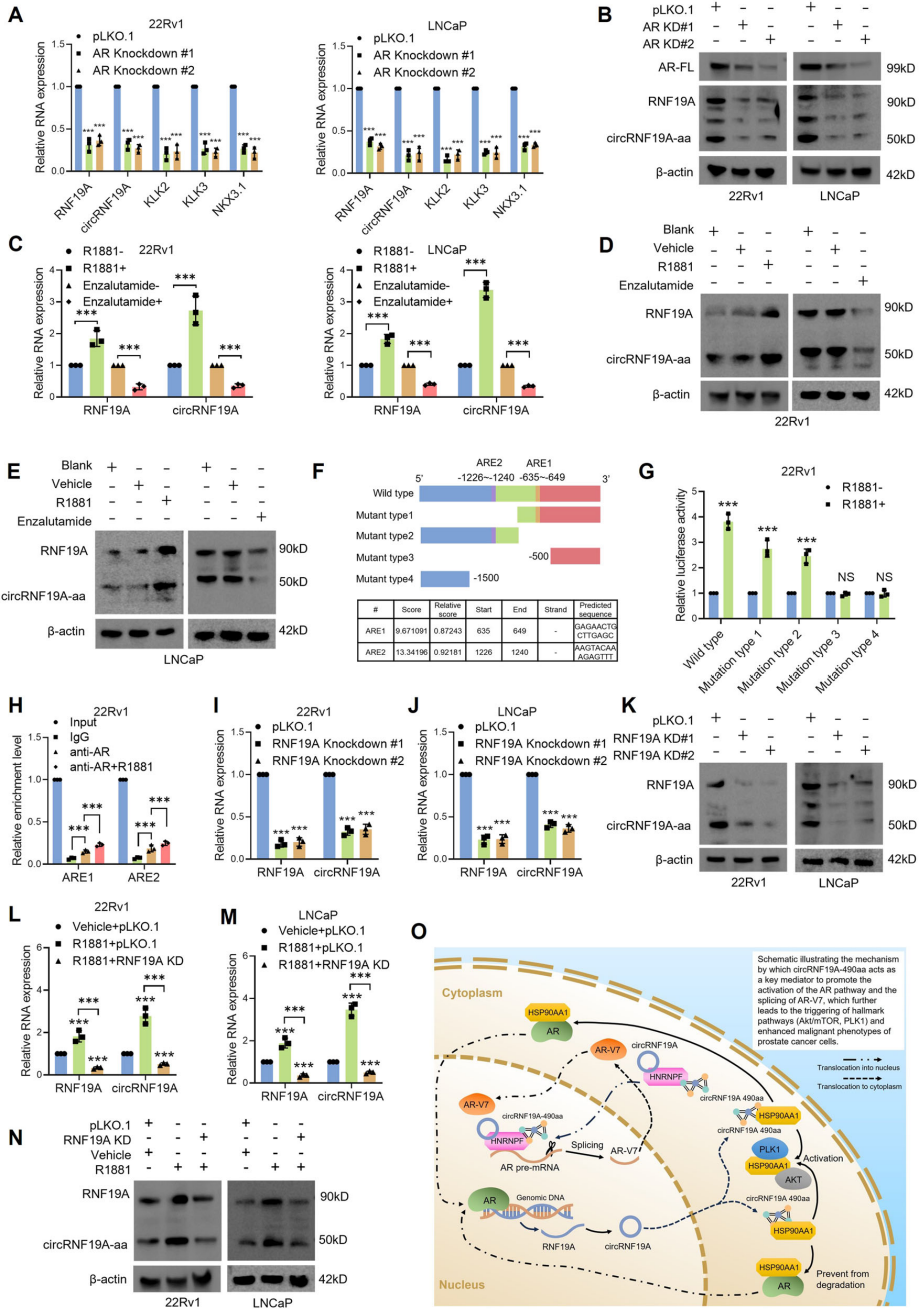
circRNF19A is regulated by AR in an RNF19A-dependent manner. **A** qRT‒PCR was performed in control, AR-knockdown 22Rv1 (left) or LNCaP cells (right) to detect the expression of RNF19A, circRNF19A and three AR target genes. **B** Western blotting was performed in control or AR-knockdown 22Rv1 or LNCaP cells to detect the expression of RNF19A and circRNF19A-490aa. **C** qRT‒PCR was performed in 22Rv1 (left) or LNCaP cells (right) under androgen-deprived (10 µM enzalutamide for 24 h) or normal androgen conditions (1 nM R1881 for 24 hours) to detect the expression of RNF19A and circRNF19A. **D**, **E** Western blotting was performed in 22Rv1 (upper) or LNCaP cells (lower) under androgen-deprived and normal androgen conditions to detect the expression of RNF19A and circRNF19A-490aa. **F** Schematic structure of the truncated RNF19A promoter region (2000 bp upstream) and the predicted androgen response elements (AREs) within the RNF19A promoter. **G** Dual-luciferase reporter assays were performed in 22Rv1 cells transfected with luciferase constructs containing the wild-type or mutated RNF19A promoter with or without R1881 stimulation. **H** Enrichment of the ARE1 or ARE2 fragment precipitated by IgG or AR antibodies in 22Rv1 cells with or without R1881 stimulation was evaluated by ChIP assay. **I**, **J** qRT‒PCR was performed in control, RNF19A knockdown 22Rv1 (left) or LNCaP cells (right) to detect the expression of RNF19A and circRNF19A. **K** Western blotting was performed in control or RNF19A knockdown 22Rv1 or LNCaP cells to detect the expression of RNF19A and circRNF19A-490aa. **L**, **M** qRT‒PCR was performed in control, RNF19A knockdown 22Rv1 (left) and LNCaP cells (right) with and without R1881 stimulation to detect the expression of RNF19A and circRNF19A. **N** Western blotting was performed in control or RNF19A-knockdown 22Rv1 and LNCaP cells with and without R1881 stimulation to detect the expression of RNF19A and circRNF19A-490aa. **O** Schematic diagram illustrating the AR-RNF19A-circRNF19A-AR/AR-V7 cascade. In prostate cancer cells, the androgen receptor regulates the transcriptional activity of RNF19A and the expression of circRNF19A and circRNF19A-490aa. circRNF19A-490aa regulates the malignancy of PCa through two mechanisms: by interacting with HSP90AA1, circRNF19A-490aa regulates the activity of the AR, Akt/mTOR and PLK1 pathways and enhances the proliferative and antiapoptotic capacities of PCa cells. In addition, circRNF19A-490aa interacts with HNRNPF and recruits HNRNPF to the splicing site of AR-V7 to promote the generation of AR-V7, which leads to the activation of AR-V7 expression and the CRPC phenotype of PCa cells. circRNF19A functions as a key mediator of crosstalk between the AR pathway and the generation of AR-V7. NS indicates not significant, *p < 0.05, **p < 0.01, and ***p < 0.001.

RNF19A reportedly acts as an oncodriver and is regulated by androgen receptors in PCa, and the expression of RNF19A is closely correlated with the development of Gleason score and a castration-resistant phenotype [47]. Therefore, we further investigated whether AR regulates RNF19A as a transcription factor and how the AR/RNF19A axis influences the expression of circRNF19A and circRNF19A-aa in PCa cells. The TCGA data confirmed a strong correlation between the expression of AR and RNF19A in PCa tumors (*R* = 0.507, *p* < 0.001, Pearson’s correlation, Fig. S4F). shRNAs targeting the AR LBD were used to knock down AR in 22Rv1 and LNCaP cells, and qRT‒PCR analysis was performed to evaluate the expression of RNF19A, circRNF19A, and the AR targets KLK2, KLK3, and NKX3.1. Western blot analysis was used to evaluate the expression of AR, RNF19A and circRNF19a-aa. As shown in Fig. 8A, AR KD significantly increased the levels of RNF19A, circRNF19A, KLK2, KLK3, and NKX3.1 according to qRT‒PCR analysis, and the results of western blotting confirmed that the expression of AR, RNF19A, and circRNF19A was notably inhibited in response to AR knockdown (Fig. 8B). Next, we added R1881 or enzalutamide to the cells to simulate androgen-activated or androgen-deprived conditions. The qRT‒PCR and western blotting results showed that R1881 treatment significantly activated AR signaling and enhanced the expression of RNF19A, circRNF19A, and circRNF19a-aa. In contrast, androgen deprivation dramatically inhibited AR signaling and decreased the expression of RNF19A, circRNF19A, and circRNF19A-aa (Fig. 8C–E).

To investigate the binding between AR and RNF19A, we analyzed the promoter region (2000 bp upstream) of RNF19A and discovered two putative androgen response elements (AREs), which are located from −635 to −645 bp and from −1226 to −1240 bp. Luciferase reporter constructs containing truncations of the RNF19A promoter were designed and constructed for further validation (Fig. 8F). Subsequently, a dual luciferase assay was performed to assess the interaction between AR and RNF19A. Five luciferase reporter constructs containing the wild-type RNF19A promoter or RNF19A mutants 1 to 4 were transfected into 22Rv1 cells, and the cells were stimulated with R1881. AR activation significantly enhanced the transcription of the wild-type RNF19A promoter, RNF19A mutation 1 and RNF19A mutation 2 but had no effect on the transcriptional level of mutation 3 or mutation 4 (Fig. 8G). Additionally, ChIP assays revealed that R1881 stimulation significantly increased the enrichment of ARE1 and ARE2 in the DNA products precipitated by AR antibodies in 22Rv1 cells (Fig. 8H). The above results indicate that AR transcriptionally regulates RNF19A through direct binding to the androgen response elements within the RNF19A promoter.

We then investigated whether AR regulates the expression of circRNF1A and circRNF19A-aa by activating the transcription of their parent gene RNF19A. RNF19A was knocked down in 22Rv1 and LNCaP cells, and qRT‒PCR and western blotting revealed a dramatic decrease in the expression of circRNF19A and circRNF19A-aa (Fig. 8I–K), indicating that the generation of circRNF19A paralleled the expression of its parent gene. Next, the cells were treated with R1881 to stimulate AR activity, and then R1881-treated cells were transduced with AR-shRNA to knock down AR. qRT‒PCR and western blotting revealed that R1881 treatment significantly increased the expression of circRNF19A and circRNF19A-aa, whereas AR KD significantly inhibited the overexpression of circRNF19A and circRNF19A-aa, which was otherwise increased by R1881 treatment alone (Fig. 8L–N). Taken together, our results indicate that AR regulates the transcription of RNF19A, which further alters the expression of circRNF19A and circRNF19A-aa.

## Discussion

The incidence of prostate cancer has been increasing over the last few decades. Although great progress has been made in early PCa diagnosis and therapeutic strategies, such as androgen deprivation therapy, dendritic cell-based immunotherapy and chimeric antigen receptor T (CAR-T) cell therapy, there are still challenges in the treatment of castration-resistant prostate cancer due to tumor drug resistance. This study elucidates the biological function of circRNF19A-490aa in prostate cancer through two key mechanisms. One mechanism by which circRNF19A-490aa promotes the malignant phenotype of prostate cancer cells is through binding to HSP90AA1, which in turn activates hallmark pathways such as the PI3K/AKT pathway. Secondly, it has been demonstrated to promote the expression of AR-V7 and the progression of castration-resistant prostate cancer (CRPC) by binding to HNRNPF, which in turn promotes the alternative splicing of the AR-V7 gene. These two mechanisms exert their effects on prostate cancer in different ways. The former elucidates how circRNF19A-490aa affects the malignant phenotype of prostate cancer cells, as evidenced by its promotion of proliferation, migration, invasion, and resistance to chemotherapy. Furthermore, the circRNF19A-490aa/HNRNPF/AR-V7 pathway not only elucidates the mechanism of prostate cancer progression to the castration-resistant stage, which is a hallmark event in prostate cancer, but also provides insight into the mechanism by which AR indirectly promotes the alternative splicing of AR-V7. (Fig. 8O).

Heterogeneous nuclear ribonucleoprotein F belongs to the HNRNP family of paralogous splicing factors and activates alternative splicing when bound to the exons of downstream targets [48, 49]. HNRNPF has been reported to activate MYC-dependent HRAS splicing and maintain the proliferation of PCa cells [50]. We have shown that the histone demethylase KDM3A recruits HNRNPF to exon 3b of AR gDNA to facilitate the alternative splicing of AR-V7 [21]. In the present study, we showed that HNRNPF is significantly overexpressed in prostate tumor tissues, and data from the TCGA indicated that HNRNPF expression is closely related to the clinical progression of PCa, suggesting that HNRNPF plays a tumorigenic role in prostate cancer. Loss-of-function experiments confirmed that HNRNPF KD significantly inhibited the survival and antiapoptotic capacity of PCa cells. Our data also showed that circRNF19A-aa interacts with HNRNPF and recruits HNRNPF to the splice site of AR-V7 to facilitate alternative splicing, which may counteract the antitumor effects of enzalutamide on PCa cells and lead to the CRPC phenotype. We also found that AR regulates the expression of circRNF19A and circRNF19A-aa by activating the transcription of RNF19A. Therefore, it is likely that AR initiates the generation of AR-V7 by controlling the expression of circRNF19A and circRNF19A-aa.

Alternative splicing is one of the key regulatory mechanisms controlling the expression of transcript variants, and dysregulation of AS has been implicated in the tumorigenesis of many human cancers [51, 52]. AS can be regulated by RNA-binding proteins, and by interacting with the AS sites of target pre-mRNAs, RBPs recruit other splicing factors and perform RNA slicing [53, 54]. AR-V7 is the major form of the AR variant and functions as the core driver in modulating the drug resistance of CRPC [21]. In our study, we demonstrated that the circRNF19A-aa/HNRNPF complex enhances the splicing of AR-V7, leading to a CRPC-like phenotype. Studies have elucidated the underlying mechanisms by which circRNAs regulate alternative RNA splicing in cancer cells. In clear cell renal cell carcinoma, circPPAP2B interacts with HNRNPC to facilitate nuclear translocation, and circPPAP2B modulates the interaction between HNRNPC and splicing factors to regulate pre-mRNA alternative splicing [55]. In gastric cancer, circURI1 inhibits metastasis by interacting with HNRNPM to modulate alternative splicing of target genes [56]. In our study, we reported that the polypeptides transcribed by circRNAs, rather than the circRNAs themselves, regulate the AS of mRNAs, which is novel and different.

It is well established that circRNAs can be translated into peptides and facilitate the development of human cancers. Research has explored the coding potential of circRNAs in a variety of tumors, including gliomas [57], hepatocellular carcinoma [58], colon cancer [59], and breast cancer [60]. To date, very few studies have investigated the protein-coding potential of circRNAs in prostate cancer. We reviewed recent studies investigating novel circRNAs associated with PCa progression, but most of the underlying mechanisms are related to RNA binding proteins [61, 62] and miRNA sponging [63, 64]. In the present study, we examined the IRESs and ORFs of DE-circRNAs to identify potential protein-coding circRNAs, and those containing both an ORF and an IRES were selected for additional validation. Finally, circRNF19A was shown to have not only oncogenic potential but also protein-coding ability. We validated the expression of circRNF19A-aa in PCa cells and tumor samples and elucidated its biological behavior and underlying mechanisms in the progression of CRPC. Our data may lead to the identification of a novel therapeutic target for the future treatment of CRPC.

circRNF19A is generated from RNF19A, a member of the RING-in-between-RING (RBR) E3 ubiquitin ligase family [65]. However, the functions and mechanisms of RNF19A in cancer remain largely unknown. Notably, the amplification of RNF19A mRNA is a common phenomenon in many human cancers [66], suggesting a specific role for RNF19A in cancer. In small cell lung cancer, RNF19A is overexpressed and promotes cancer growth by mediating P53 ubiquitin-mediated degradation [67]. In prostate cancer, Zhang et al. used a CRISPR system to screen for E3 ubiquitin ligases and identified RNF19A as a novel oncodriver in PCa [47]. They reported that RNF19A is highly expressed in PCa and is correlated with an advanced Gleason score and a castration-resistant phenotype. Transcriptomic and ubiquitination proteomic analysis revealed that RNF19A is transcriptionally regulated by AR. In our study, we identified two AREs in the promoter of RNF19A and verified the interaction between AR and RNF19A by ChIP and dual-luciferase assays. In another study, Zhu et al. reported that despite the frequent amplification of RNF19A in cancer, RNF19A increases cell sensitivity to PARP inhibitors by inhibiting homologous recombination (HR)-mediated DNA damage repair [66]. In the present study, we showed that RNF19A modulates PCa progression through its circRNA form and enhances the antiapoptotic capacity and enzalutamide resistance of PCa cells.

In conclusion, the present study revealed the oncogenic role of a novel circRNA, circRNF19A, in the development of prostate cancer. The novel protein circRNF19A-490aa, encoded by circRNF19A, promoted the malignant behavior and drug resistance of PCa cells by interacting with two critical oncoproteins, HSP90AA1 and HNRNPF. circRNF19A-490aa enhanced AR, Akt/mTOR and PLK1 pathway activity in PCa cells by interacting with HSP90AA1. Additionally, circRNF19A-490aa interacted with HNRNPF, recruited HNRNPF to the AR-V7 splice site and accelerated the alternative splicing of AR-V7. Furthermore, AR promoted the splicing of circRNF19A by transcriptionally binding to the promoter of RNF19A. Our data demonstrated that circRNF19A plays a key role in the constitutive activation of AR and AR-induced overexpression of AR-V7 in PCa cells.

## Supplementary information

Supplementary files

Supplementary Tables

Original western blots

## Data Availability

The RNA-Seq datasets have been uploaded to The National Omics Data Encyclopedia (https://www.biosino.org/node/) under accession number PRJNA1052995. The datasets analyzed in the present study are available in the TCGA repository (https://portal.gdc.cancer.gov/). The dataset supporting the conclusions of this article is available from the corresponding author upon reasonable request.
