# Supplementary material for: Androgen-targeted hsa_circ_0085121 encodes a novel protein and improves the development of prostate cancer through facilitating the activity of PI3K/Akt/mTOR pathway and enhancing AR-V7 alternative splicing
Source: Cell Death Dis. 2024 Nov 20;15(11):848. doi: 10.1038/s41419-024-07246-9 (PMC11579034; doi:10.1038/s41419-024-07246-9)
Supplement: Supplementary file 1 — Supplementary files [file 41419_2024_7246_MOESM1_ESM.pdf]

Supplementary materials for "Androgen-targeted hsa_circ_0085121 encodes a novel protein and improves the development of prostate cancer through facilitating the activity of PI3K/Akt/mTOR pathway and enhancing AR-V7 alternative splicing."

Jianfeng Li[*1], Hui Qiu[*2], Qingzhuo Dong[*1], Hongyuan Yu[1], Chiyuan Piao[1], Zhengxiu Li[3], Yanbin Sun[#4] and Xiaolu Cui[#1]

1. Department of Urology, First Hospital of China Medical University, #155 Nanjing North Road, Shenyang, 110001, China.
2. Department of Gynecology and Obstetrics, Shengjing Hospital of China Medical University, #36 Sanhao Street, Shenyang, 110004, China.
3. Department of Dermatology, First Hospital of China Medical University, #155 Nanjing North Road, Shenyang, 110001, China.
4. Department of Thoracic Surgery, First Hospital of China Medical University, #155 Nanjing North Road, Shenyang, 110001, China.

Supplementary Figures

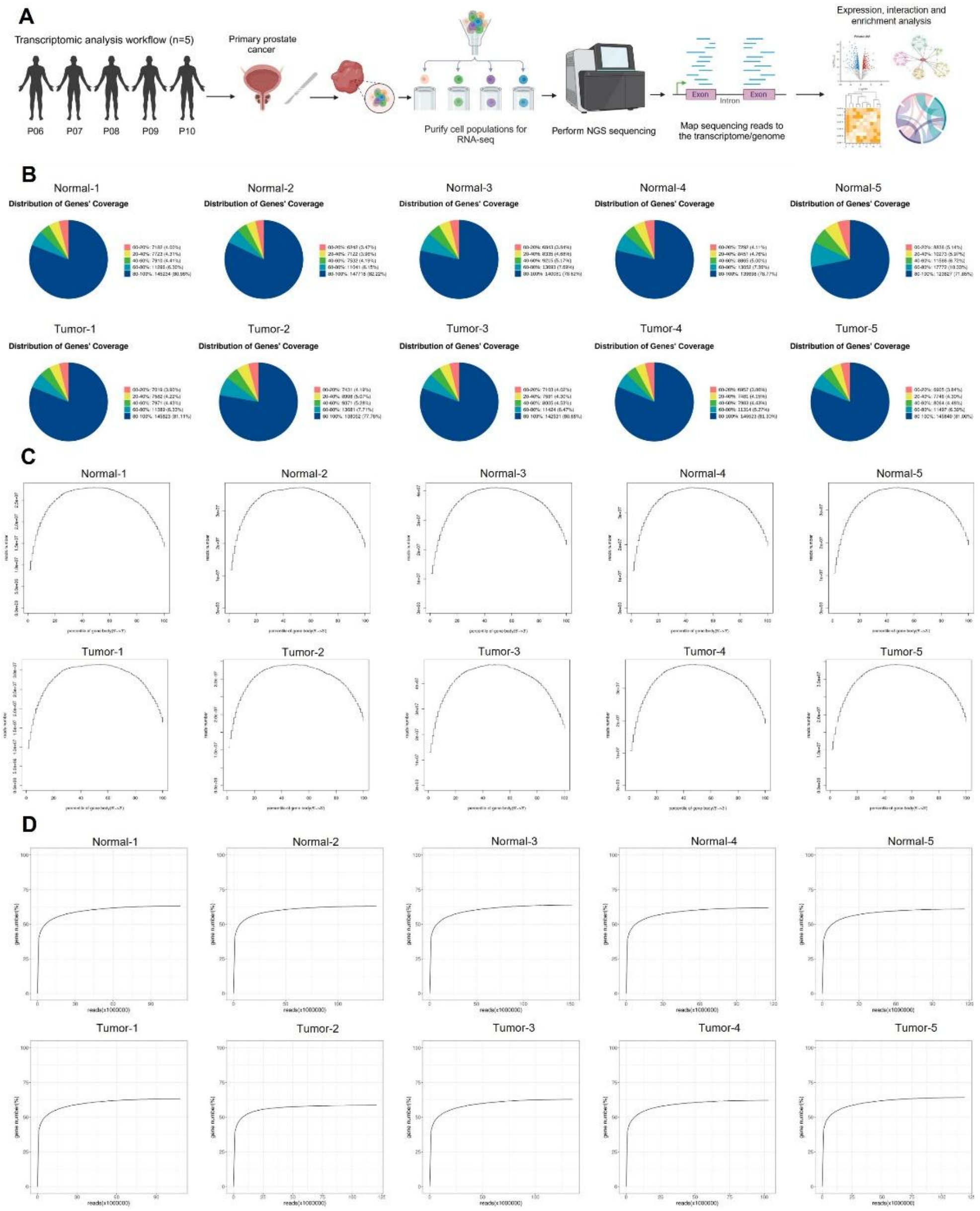

Figure S1. Quality control test of the transcriptomic sequencing data.

(**A**) The workflow of the transcriptomic analysis design. (**B**) Distribution of gene coverage in each sample. (**C**) Homogeneity of transcriptional coverage. (**D**) Sequencing saturation results for each sample.

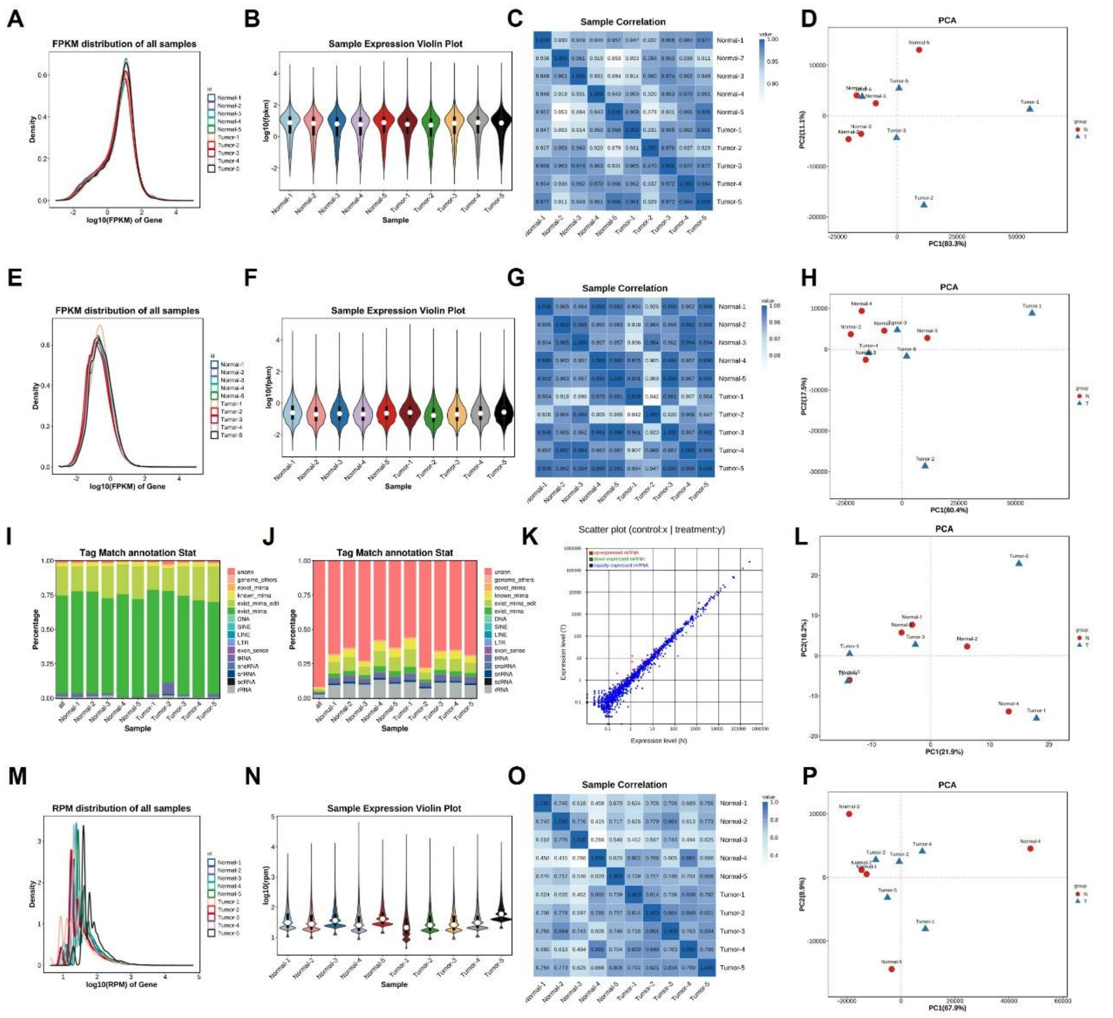

Figure S2. Distribution of gene expression abundance and correlation analysis of the samples.

(**A**) FPKM (mRNA expression abundance) distribution of all samples. (**B**) Violin plot demonstrating the mRNA gene expression abundance in each sample. (**C**) Heatmap showing the correlation analysis of mRNA gene expression abundance in each sample. (**D**) Principal component analysis (PCA) of the samples (based on mRNA gene expression). (**E**) FPKM (lncRNA expression abundance) distribution of all samples. (**F**) Violin plot demonstrating the lncRNA gene expression abundance in each sample. (**G**) Heatmap showing the correlation analysis of lncRNA gene expression abundance in each sample. (**H**) Principal component analysis (PCA) of the samples (based on lncRNA gene expression). (**I**) Statistical analysis of the abundance of annotated tags for each sample. (**J**) Statistical analysis of the types of annotation tags for each sample. (**K**) Scatter plot indicating the differentially expressed miRNAs in the samples. (**L**)

Principal component analysis (PCA) of the samples (based on miRNA gene expression). (**M**) FPKM (circRNA expression abundance) distribution of all samples. (**N**) Violin plot demonstrating the circRNA gene expression abundance in each sample. (**O**) Heatmap showing the correlation analysis of circRNA gene expression abundance in each sample. (**P**) Principal component analysis (PCA) of the samples (based on circRNA gene expression).

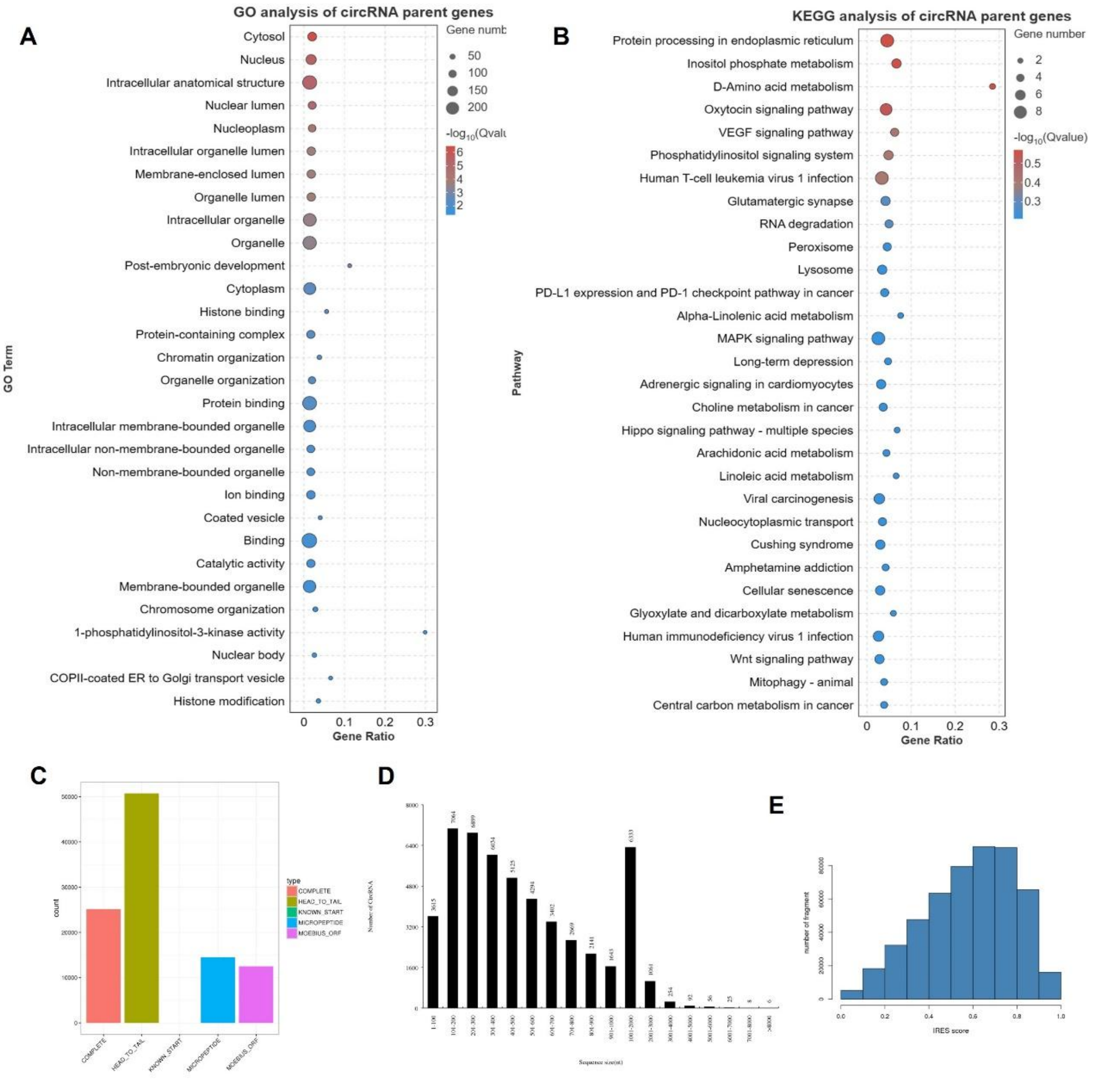

Figure S3. Analysis of the coding potential of circRNAs.

(**A**) Bar chart showing the counts of different attributes of ORFs. The ORFs are divided into five types based on their attributes. a) COMPLETE ORF: the coding region covers the entire cyclic RNA, resulting in full-circle translation. b)

HEAD_TO_TAIL: the coding region skips the junction site; c) MOEBIUS_ORF: there is no termination codon in the ORF, which may lead to infinite translation (or rolling translation); d) KNOWN_START: the start codon of circRNA is the same as that of the ORF of mRNA; e) MICROPEPTIDE: polypeptides smaller than 100 amino acids. (**B**) The distribution of the length of the ORFs. (**C**) Frequency histogram of the IRES scores.

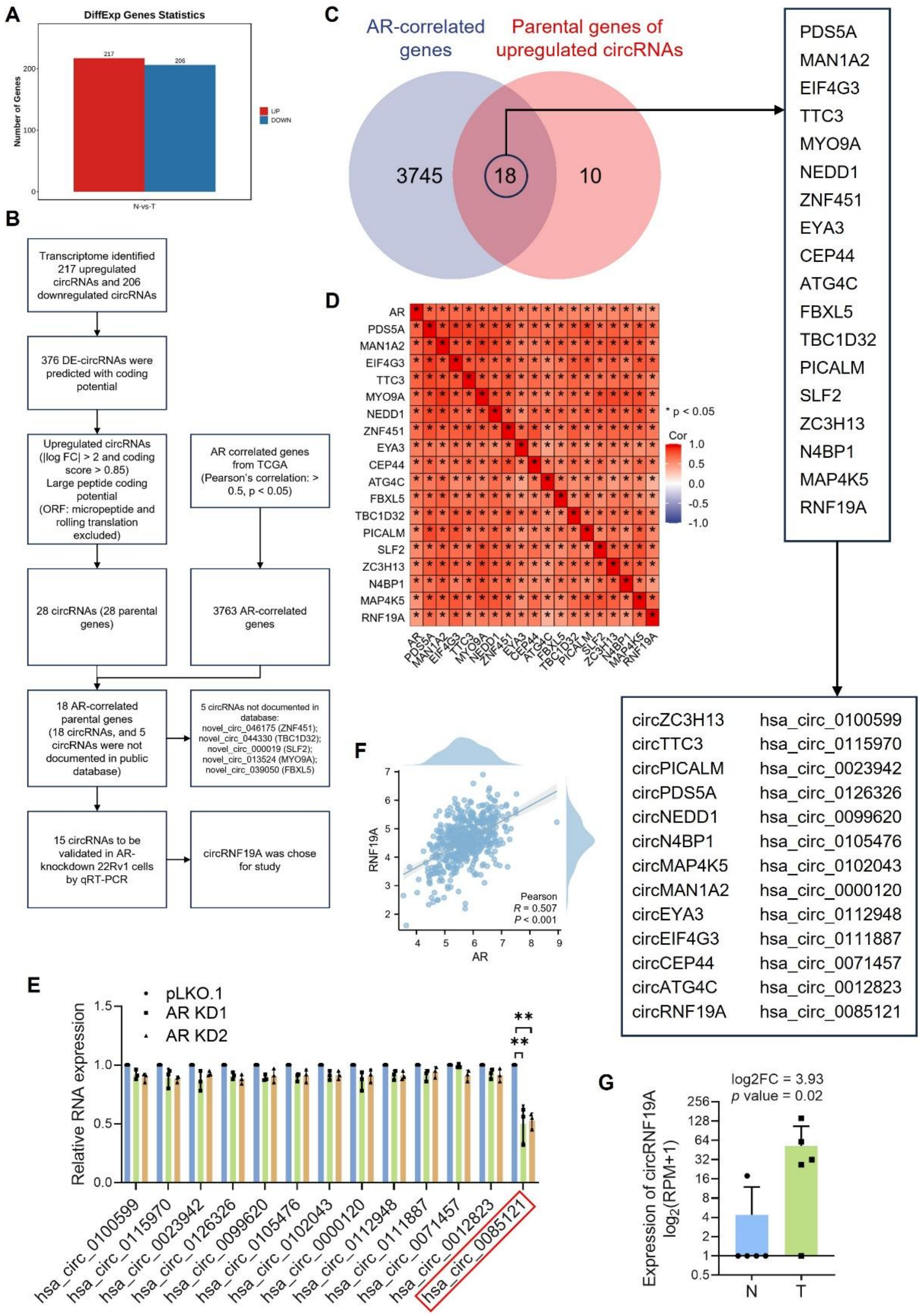

Figure S4.

(**A**) Number of circRNAs detected by transcriptome. (**B**) Brief workflow of screening for circRNA candidates in this study. (**C**) 18 genes were shared by AR-correlated genes (TCGA, Pearson's correlation > 0.5, p < 0.05) and parental genes of upregulated circRNAs in PCa tissues, and the circRNAs generated from the 18 (parental) genes

were searched in circBase, and 15 out of 18 circRNAs were found to be documented in circBase. (**D**) The heatmap of the 18 genes in (**C**) showing the correlations of the expression of 18 genes with AR in TCGA_PRAD. (**E**) qRT-PCR was performed to measure the expression of 15 candidate circRNAs in control and AR knocked down 22Rv1 cells. (**F**) Data from TCGA_PRAD shows the correlation between expression of RNF19A and AR. (**G**) The expression of circRNF19A in adjacent normal prostate tissues and PCa tissues were detected by transcriptome.

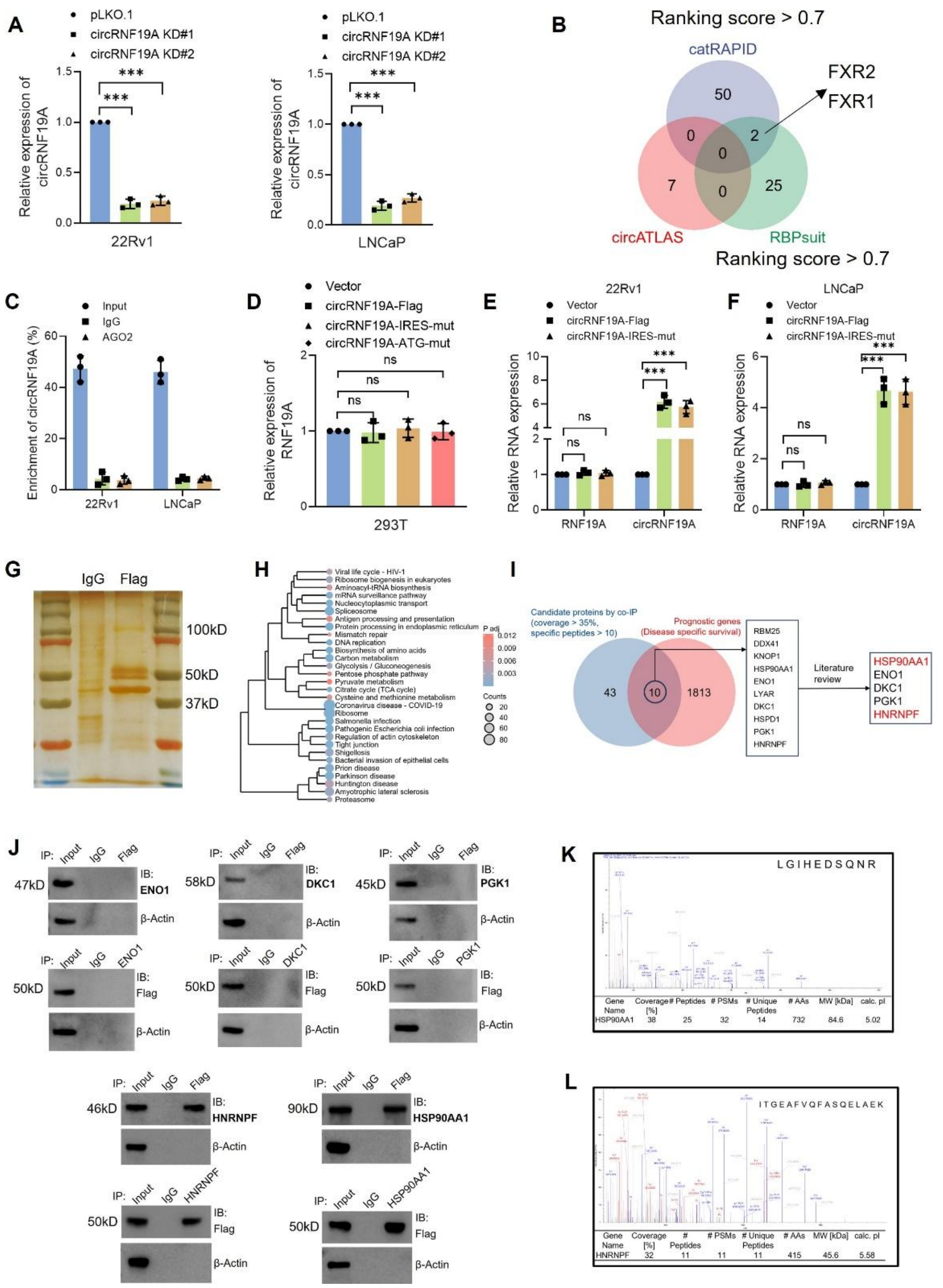

Figure S5.

(**A**) The expression of circRNF19A was detected in circRNF19A-knockdown 22Rv1 cells and LNCaP cells by qRT-PCR. (**B**) The RBPs with circRNF19A were predicted by circRAPID, circATLAS and RBPsuit. (**C**) The enrichment of circRNF19A in the RNA products precipitated by indicated anti-bodies was assessed by RIP assay. (**D**) Expression of linear RNF19A mRNA was detected via qRT–PCR in 293T cells (control cells, circRNF19A-Flag-transfected cells, circRNF19A-IRES-mut-transfected

cells, and circRNF19A-ATG-mut-transfected cells). The expression of RNF19A and circRNF19A was detected in 22Rv1 (**E**) and LNCaP (**F**) cells (control cells, circRNF19A-Flag-transfected cells or circRNF19A-IRES-mut-transfected cells) by qRT–PCR. (**G**) 22Rv1 cells were transfected with circRNF19A-Flag, and an anti-FLAG antibody was used to perform the co-IP assay. After that, total proteins immunoprecipitated by the Flag antibody were separated by SDS–PAGE, and the entire lane of the gel was removed and subjected to liquid chromatography–tandem mass spectrometry (LC–MS–MS) analysis. (**H**) A total of 955 precipitated proteins were identified by LC–MS analysis, and KEGG pathway enrichment analysis was performed to evaluate the biological functions of the proteins. (**I**) 10 genes were shared by prognostic-related genes (PCa specific survival, 1823 genes) and candidate proteins (genes) by LC-MS analysis (53 proteins), and after literature study, 5 proteins were selected for validation. (**J**) Co-IP was performed to measure the interactions between circRNF19A-490aa and candidate proteins in Flag-circRNF19A-transfected 22Rv1 cells. (**K**) LC−MS analysis was performed to identify the peptide sequence (LGIHEDSQNR) of HSP90AA1. (**L**) LC−MS analysis was performed to identify the peptide sequence (ITGEAFVQFASQELAEK) of HNRNPF. ns indicates not significant, * indicates $p < 0.05$, ** indicates $p < 0.01$, *** indicates $p < 0.001$.

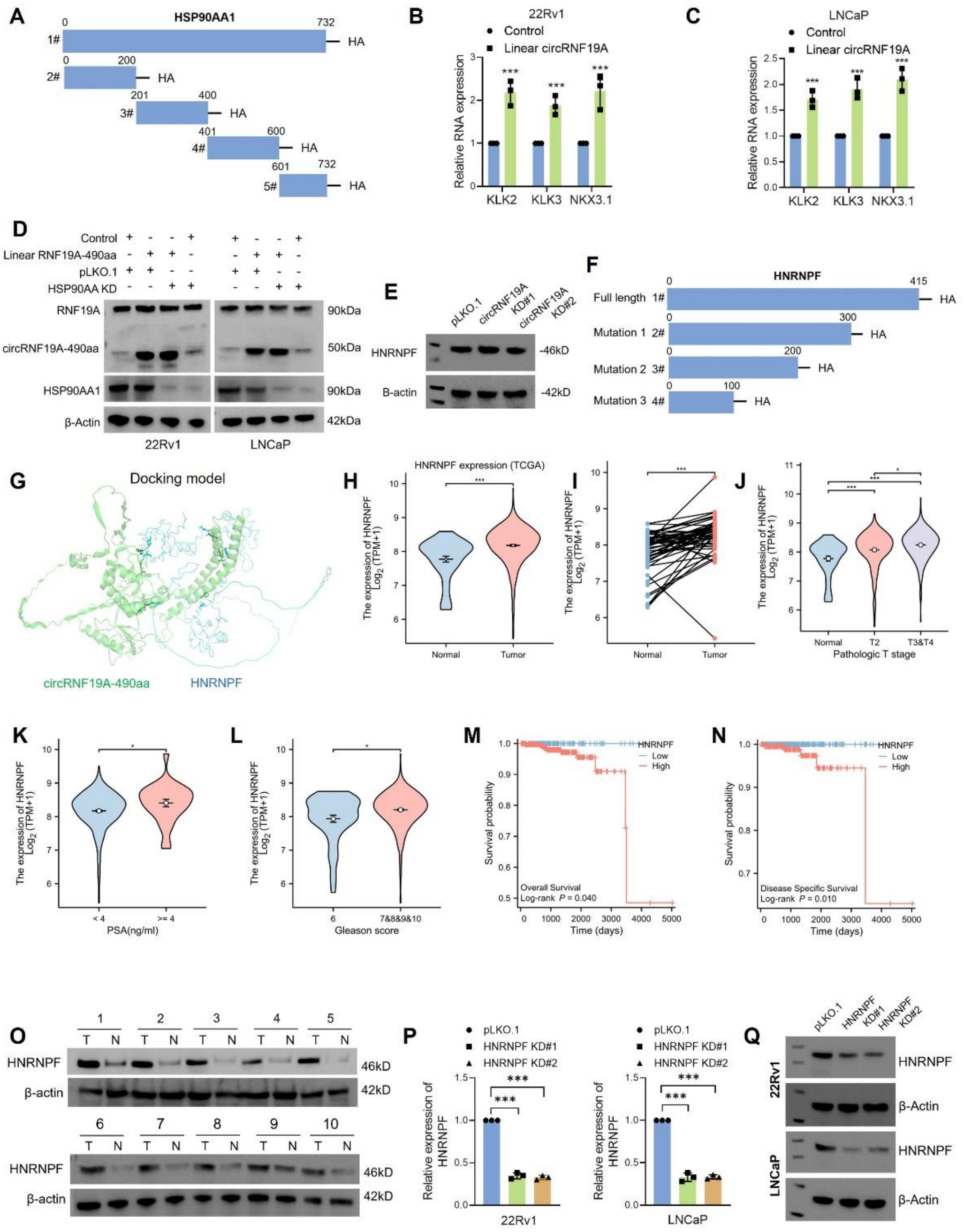

Figure S6.

(**A**) Schematic illustration indicating the truncated mutations of the HSP90AA1 protein. The full-length sequence of HSP90AA1 was divided into four segments and labeled with HA. (**B, C**) In 22Rv1 and LNCaP cells, overexpression of circRNF19A-490aa significantly increased the expression of AR target genes, as determined by qRT–PCR. (**D**) Western blotting assay were performed to measure the expression of RNF19A, circRNF19A-aa, and HSP90AA1 in each group of engineered PCa cells. (**E**) HNRNPF expression was detected in 22Rv1 cells (control or circRNF19A-

knockdown) by western blotting. (**F**) Schematic diagram illustrating the structures of full-length or truncated HNRNPF mutants. (**G**) The docking model between HNRNPF and circRNF19A-aa was illustrated by PyMOL. (**H**) HNRNPF expression in normal prostate tissues (n=52) and PCa tumor tissue samples (n=499) was detected, and the data were obtained from the TCGA. (**I**) HNRNPF expression in 52 pairs of normal prostate tissues and PCa tumor tissue samples was detected, and the data were obtained from the TCGA. (**J, K, L**) Relationships between HNRNPF expression and T stage, PSA level and Gleason score were analyzed, and the data were obtained from the TCGA. (**M, N**) Kaplan–Meier analysis was performed to assess the correlations between HNRNPF expression and overall survival and disease-specific survival in PCa patients. (**O**) HNRNPF expression was detected in ten pairs of PCa tumor samples and paired normal prostate tissue samples by western blot. (**P**) qRT-PCR and (**Q**) Western blotting were performed in 22Rv1 and LNCaP cells transfected with pLKO.1 or HNRNPF shRNAs to detect the expression of HNRNPF. ns indicates not significant, * indicates $p < 0.05$, ** indicates $p < 0.01$, *** indicates $p < 0.001$.

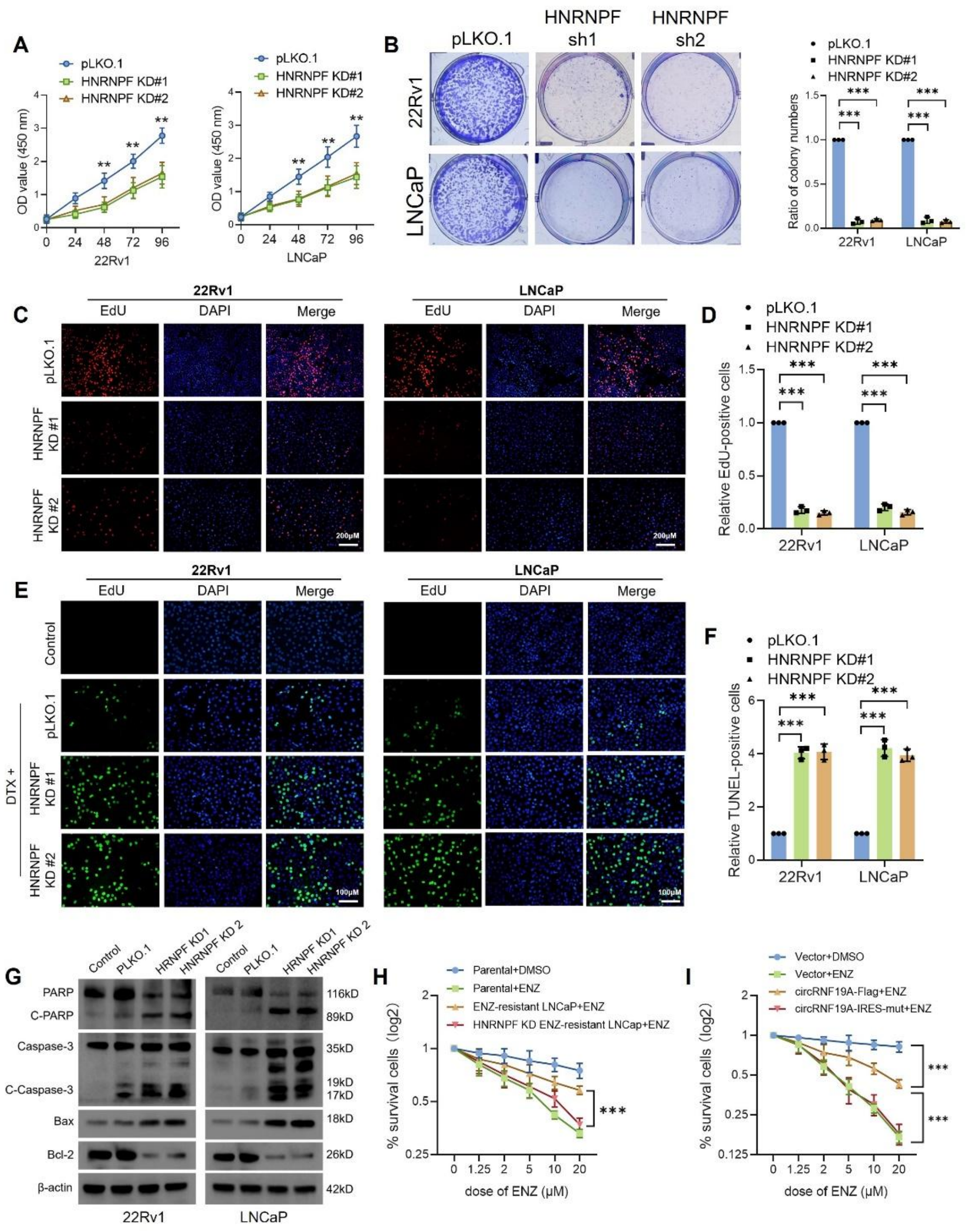

Figure S7.

(**A**) CCK-8, colony formation (**B**) and EdU (**C, D**) assays were performed in 22Rv1 and LNCaP cells transfected with pLKO.1 or HNRNPF shRNAs to measure the cell proliferative capacity. Magnification = 100×. (**E, F**) TUNEL staining was performed in 22Rv1 and LNCaP cells transfected with pLKO.1 or HNRNPF shRNAs to measure the level of cell apoptosis. Magnification = 200×. Cells were treated with DMSO (control group) or 10 nM docetaxel (DTX) for 24 hours. (**G**) In line with the results of the TUNEL staining assay, western blotting was performed for each group of engineered cells to measure the expression of apoptotic markers. (**H**) The parental

LNCaP cells and engineered enzalutamide-resistant LNCaP cells (control or HNRNPF-depleted) were treated with increasing doses of enzalutamide. A CCK-8 assay was performed for each group of cells to evaluate the cell survival rate. (**I**) Engineered LNCaP cells (control, circRNF19A-Flag-transfected or circRNF19A-IRES-mut-transfected) were treated with increasing doses of enzalutamide. ns indicates not significant, * indicates $p < 0.05$, ** indicates $p < 0.01$, *** indicates $p < 0.001$.

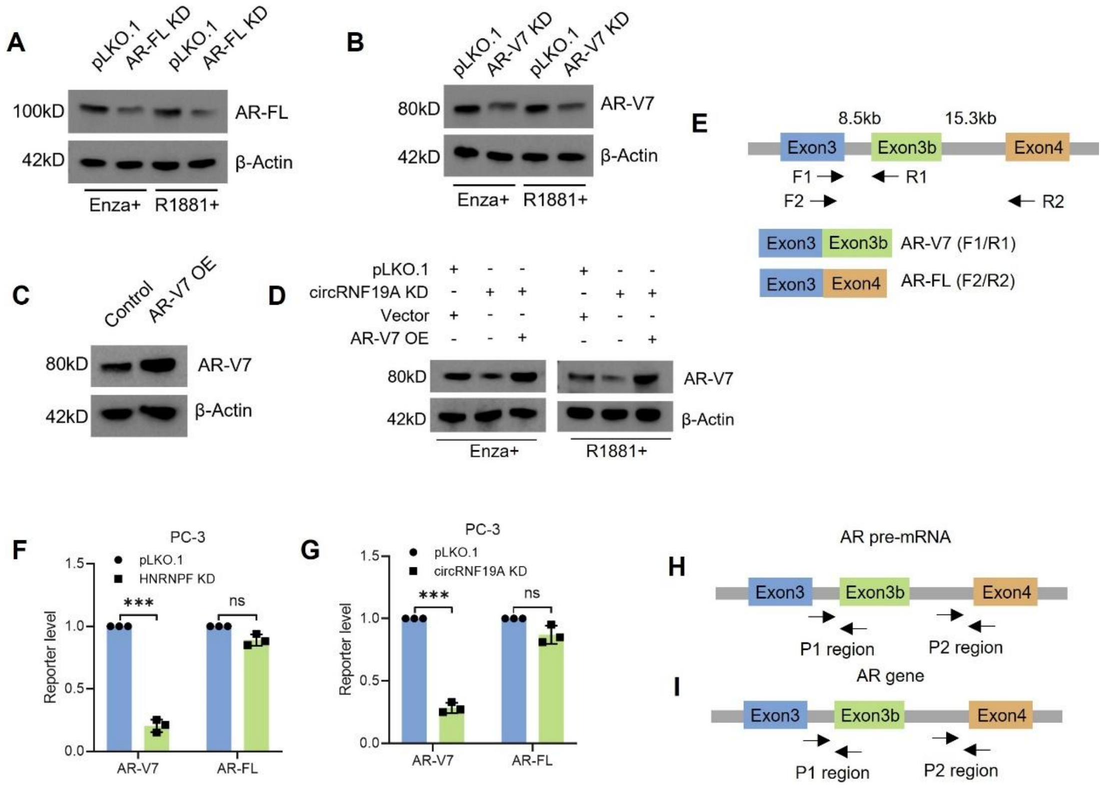

Figure S8.

(**A**) Western blotting was performed in control or AR-FL-depleted 22Rv1 cells under androgen-deprived and normal androgen conditions to measure the expression of AR-FL. (**B**) Western blotting was performed in control or AR-V7-depleted 22Rv1 cells under androgen-deprived and normal androgen conditions to measure the expression of AR-V7. (**C**) Western blotting was performed in control or AR-V7-overexpressing 22Rv1 cells to measure the expression of AR-V7. (**D**) Western blotting was performed in control, circRNF19A-depleted or circRNF19A-depleted and AR-V7-

overexpressing 22Rv1 cells under androgen-deprived and normal androgen conditions to measure the expression of AR-V7. (**E**) Diagram of the AR minigene reporter. The exons, introns, and PCR primers used to detect exon splicing are indicated in the diagram. Splicing between exons 3 and 3b reflects the formation of AR-V7 mRNA, which can be detected by the F1/R1 primer pair. Splicing between exons 3 and 4 reflects the formation of AR-FL mRNA, which can be detected by the F2/R2 primer pair. (**F, G**) AR-negative PC3 cells (pLKO.1/HNRNPF KD or pLKO.1/circRNF19A KD) were transfected with the AR minigene reporter. Seventy-two hours after transfection, total RNA was extracted and analyzed via qRT–PCR using the F1/R1 or F2/R2 primer pair, which reflects the levels of AR-V7 or AR-FL, respectively. (**H**) Diagram showing the primers used to amplify the P1 or P2 region of AR pre-mRNAs in the RIP assays. (**I**) Diagram showing the primers used to amplify the P1 or P2 region of the AR gene in the ChIP assays. ns indicates not significant, * indicates $p < 0.05$, ** indicates $p < 0.01$, *** indicates $p < 0.001$.

Supplemental Methods

RNA extraction, strand-specific library construction and sequencing

For mRNA and circRNA sequencing analyses, total RNA from each sample was isolated with TRIzol reagent (Invitrogen) according to the manufacturer's instructions. A strand-specific library was constructed using the NEBNext® Poly(A) mRNA Magnetic Isolation Module and sequenced using an Illumina NovaSeq 6000 by Gene Denovo Biotechnology (Guangzhou). For miRNA sequencing analysis, isolated total RNAs (with a size range of 18–30 nt) were enriched via PAGE. 3′ and 5′ adapters were ligated to RNAs, and ligation products were reverse-transcribed by PCR amplification, enriched to generate a cDNA library, and sequenced as described above. High-quality clean reads were further filtered by fastp (version 0.18.0). Using Ensembl_release 110 as the reference genome, an index of the reference genome was established, and paired-end clean reads were mapped to the reference genome using HISAT (version 2.1.0) with "-rna-strandness RF" and other parameters set as defaults. The mapped reads of each sample were assembled by using StringTie (version 1.3.4)

via a reference-based approach. For each transcription region, a TPM (transcripts per kilobase of exon model per million mapped reads) value was calculated to quantify the expression abundance and variation of the region using RSEM software.

Quantification of Transcripts Abundance

The mapped reads of each sample were assembled by using StringTie v1.3.1 via a reference-based approach. For each transcription region, an FPKM (fragment per kilobase of transcript per million mapped reads) value was calculated to quantify the expression abundance and variation of the region using RSEM[1] software. The FPKM formula is shown as follows:

$$FPKM = \frac{10^6 C}{NL/10^3}$$

Given that FPKM(A) is the expression of transcripts A and C is the number of fragments mapped to transcripts A, N is the total number of fragments mapped to reference genes, and L is the number of bases on transcripts A. The FPKM method is able to eliminate the influence of different transcript lengths and sequencing data amounts on the calculation of transcript expression. Therefore, the calculated transcript expression can be directly used for comparing the differences in transcript expression among samples.

Plasmids, cloning, mutagenesis and transfections

The Flag- and HA-tagged plasmids were constructed by RiboBio (Guangzhou, China). The Flag-tagged plasmids were constructed using the pCDNA3.1-FLAG-C vector, and the HA-tagged plasmids were constructed using the pCDNA3.1-HA-N vector. The sequences of circRNF19A-Flag, circRNF19A-IRES-mut-Flag, circRNF19A-ATG-mut-Flag and linear RNF19A-490aa-Flag are listed in Table S8. The sequences of HA-HSP90AA1-ful length, HA-HSP90AA1-truncated 1 (1-200 aa), HA-HSP90AA1-truncated 2 (201-400 aa), HA-HSP90AA1-truncated 3 (401-600 aa), HA-HSP90AA1-truncated 4 (601-732 aa), HA-HNRNPF-full length, HA-HNRNPF-truncated 1 (1-300), HA-HNRNPF-truncated 2 (1-200) and HA-HNRNPF-truncated 3

(1-100) are listed in Table S6. All transfection experiments were conducted with Lipofectamine™ 3000 Transfection Reagent (Invitrogen, USA) following the manufacturer's instructions. G418 (geneticin) was added to 22Rv1 and LNCaP cells transfected with the circRNF19A-Flag, circRNF19A-IRES-mut-Flag, or linear RNA19A-490 aa overexpression plasmid to select resistant colonies (stably transfected cells). G418 at a concentration of 400 mg/L was used for selection, and G418 at a concentration of 200 mg/L was used for maintenance. shRNAs that specifically target RNF19A, circRNF19A, HNRNPF, HSP90AA1, AR-FL and AR-V7 were synthesized along with the negative control pLKO.1 at RiboBio (Guangzhou, China). The lentiviral vectors were packaged in 293T cells by calcium phosphate transfection. Prostate cancer cells were transduced with the supernatant of lentiviral particles. The cells were cultured in a 6-well plate with 2 ml of culture medium, and the concentration used for lentivirus transduction was 5×10^6 transducing units of lentivirus. The stable cell lines were constructed using puromycin (200 μg/ml).

Reverse transcriptase PCR (RT–PCR) and quantitative real-time PCR (qRT–PCR)

Total RNA was extracted from 22RV1 and LNCaP cells with TRIzol (Yeasen, 19211ES60), and 1.5 μg of RNA per sample was used for cDNA synthesis with the PrimeScript™ RT Kit (TAKARA, Japan). The reaction conditions involved an initial step at 37 °C for 15 minutes, followed by 85 °C for 5 seconds. The resulting cDNA product served as an immediate template for PCR. For quantitative PCR (qPCR) analysis, the TB Green Premix Ex Taq™ Kit was utilized following the manufacturer's guidelines. The qPCR conditions were set at 95°C for 30 seconds, followed by 50 cycles of 95°C for 5 seconds and 60°C for 30 seconds. β-Actin was used as a reference for circRNF19A. Relative expression levels were computed using the $2^{(-\Delta\Delta CT)}$ method. The primers used were synthesized by BGI Technology (Beijing, China).

The sequences of primers used are shown in Supplementary Table 12 (Table S12).

AR minigene reporter assay

The AR minigene reporter assay was performed as described previously[2]. Briefly, using Lipofectamine 2000 (Invitrogen), 200 ng of the AR minigene reporter was transfected in triplicate into AR-negative PC3 cells cultured in 12-well plates. After 72 hours, RNA was extracted for qRT–PCR analysis using the following primers to evaluate AR-V7 or AR-FL splicing:

AR-V7 splicing-F: CAGGGATGACTCTGGGAGAA

AR-V7 splicing-R: GCCCTCTAGAGCCCTCATTT

AR-FL splicing-F: TCTTGTCGTCTTCGGAAATGT

AR-FL splicing-R: AAGCCTCTCCTTCCTCCTGTA

Proliferation assay

The capacity for cellular proliferation was measured using a Cell Counting Kit-8 (CCK-8) (Dojindo, Tokyo, Japan) and a cell colony formation assay according to the manufacturer's protocol. The absorbance at 450 nm was measured to determine cell viability using a 96-well plate reader. For the cell colony formation assay, the cells were plated in 24-well plates (300 cells per well) and incubated for 14 days in complete medium. Colonies were fixed with 10% formaldehyde for 10 min and stained with 1.0% crystal violet for 5 min.

The number of colonies, defined as those with > 50 cells/colony, was counted. 5-Ethynyl-2'-deoxyuridine (EdU) staining was performed using a BeyoClick™ EdU Cell Proliferation Kit with Alexa Fluor 488 (Beyotime). The cells were cultured in a 6-well plate and subjected to the indicated treatments. Then, 2× EdU buffer was added to the medium to a final concentration of 10 μM. After 24 hours, the cells were fixed and washed three times, and 0.5 ml of Click Additive Solution was added to each well. The plate was then gently shaken and incubated for 30 minutes in the dark. The red fluorescence of the proliferating cells was visualized using an inverted fluorescence microscope (Olympus, Tokyo, Japan) and captured under an original magnification of 100×. The proliferating cells were counted using ImageJ.

Immunohistochemistry (IHC)

The expression of the indicated genes in tissue specimens was detected using an UltraSensitiveTM SP (Mouse/Rabbit) IHC Kit (Maxin-Bio, Fuzhou, Fujian, China) according to the manufacturer's instructions. Briefly, sections were first dewaxed in xylene and ethanol, and antigen retrieval was performed using a microwave for 10 min at 100°C. The sections were then incubated with antibodies for 1 h, followed by incubation with biotinylated anti-IgG antibody and the streptavidin-biotinylated complex horseradish peroxidase. DAB and hematoxylin were used for nuclear staining. The images were then captured by an upright metallurgical microscope (Olympus, Tokyo, Japan) at an original magnification of 200×.

Protein–protein docking

Protein–protein docking is based on the three-dimensional structure of two known proteins, and the near-natural structure of the complex is predicted by AlphaFold2 (https://alphafold.com/). Protein docking calculations were performed by ZDOCK (https://zdock.umassmed.edu/), which is based on the rigid protein docking technology of fast Fourier transform. The docking conformation is clustered according to the ligand position, and the docking conformation is ranked by the ZRANK score. The docking posture with the highest score was visualized by PyMOL (https://pymol.org/).

RIP Assay

As previously reported[2], a RIP assay was carried out. Buffer A (10 mM HEPES (pH 7.4), 10 mM KCl, 1.5 mM MgCl2, 10% glycerol, 1 mM DTT, protease and phosphatase inhibitor mixtures, 50 U/mL RNase OUT) was used to extract the nuclei from the cells. Nuclear extracts were solubilized in RIP lysis buffer [100 mM KCl, 5 mM MgCl2, 10 mM HEPES (pH 7.4), 0.5% Nonidet P-40, 1 mM DTT, protease and phosphatase inhibitor mixtures, 100 U/mL RNase OUT] and precleared with 5 μg of control IgG and Protein A/G agarose beads (Santa Cruz Biotechnology). The samples were then subjected to immunoprecipitation with 5 μg of antibodies (control IgG or

related antibodies) overnight at 4°C, followed by incubation with Protein A/G agarose beads for 4 hours at 4°C. The beads were washed with buffer I (100 mM KCl, 5 mM MgCl2, 10 mM HEPES [pH 7.4], 1 mM DTT) three times and then with buffer II (100 mM KCl, 5 mM MgCl2, 10 mM HEPES [pH 7.4], 1 mM DTT, 1 M urea]) three times. The beads were incubated with DNase I (10 units) for 15 min at 37°C, followed by 30 μg of proteinase K for 30 min at 50°C. Precipitated RNA was extracted with TRIzol and used for cDNA synthesis and qRT–PCR analysis.

The PCR primers used to evaluate the P1 and P2 regions were as follows:

P1 region-F: ACCTCCCCAACTTTACATGCT

P1 region-R: CAGGGTCTGGTCATTTTGAGA

P2 region-F: CTGTGACCAGGGAGAATGGT

P2 region-R: CTGGAAGCCTCTCCTTCCTC

The RIP assay was performed in triplicate and repeated in three independent experiments. The results are presented as the means ± SDs (n = 3).

Chromatin immunoprecipitation (ChIP) assay

Chromatin immunoprecipitation (ChIP) was performed using a SimpleChiP™ Enzymatic Chromatin IP Kit (Cell Signaling Technology, Danvers, MA, USA) according to the manufacturer's protocol. Cells (4×10^7) in five 150-mm culture dishes were treated with 1% formaldehyde to crosslink proteins to DNA and collected. The chromatin was digested by micrococcal nuclease to a length of approximately 150-900 bp. The cross-linked chromatin was separately incubated with 10 μL of anti-AR antibody (Abcam), 3 μL of anti-IgG antibody (negative control; Cell Signaling Technology), or 3 μL of anti-histone H3 antibody (positive control; Cell Signaling Technology) overnight at 4°C with rotation. Protein G agarose beads were used to harvest the immunoprecipitants. After reverse crosslinking of the protein/DNA complexes to free the DNA, qRT–PCR was performed to assess the enrichment of target sequences. RPL30 (provided by the kit) was used as an internal reference. Precipitated DNA was also amplified for 25 cycles and resolved on a 1% agarose gel to evaluate the amplification of the target DNA.

The sequences of primers used were as follows:

ARE #1: F CAAGCCTCGAGTGCAGTG

ARE #1: R TGTTTGCCTGTAATCCCAGC

Site: -635~-649. Production size: 102 bp.

ARE #2: FGACCAAGCCAGTTTTCCCTG

ARE #2: R TGGTGGTCTACATAATCAACAGT

Site: -1226~-1240. Production size: 206 bp.

P1 region-F: ACCTCCCCAACTTTACATGCT

P1 region-R: CAGGGTCTGGTCATTTTGAGA

P2 region-F: CTGTGACCAGGGAGAATGGT

P2 region-R: CTGGAAGCCTCTCCTTCCTC

Fluorescence in situ hybridization (FISH) assay

Fluorescently labeled oligonucleotide probes complementary to circRNF19A were synthesized by GenePharma (Shanghai, China). Pretreated 22Rv1 cells (1×10^4 cells) were fixed with 4% paraformaldehyde (Beyotime), permeabilized with 0.3% Triton X-100 for 15 min, and treated with an anti-AR antibody or fluorescently labeled circRNF19A probes at 4°C overnight. After incubation with the secondary antibody and counterstaining with DAPI, the stained cells were viewed using a confocal laser scanning microscope (OLYMPUS-IX71) and processed using cellSens standard 1.6 imaging software.

TUNEL assay

A TUNEL assay was performed to assess the degree of cell apoptosis by using a TUNEL assay kit from Beyotime (Shanghai, China) according to the manufacturer's instructions. Briefly, the pretreated cells in 24-well plates were washed with PBS and fixed in 4% paraformaldehyde for 30 min. Then, the cells were washed with PBS and incubated in PBS containing 0.3% Triton X-100 for 5 min at room temperature. The cells were added to 50 μl of TUNEL assay solution and incubated for 60 min at 37°C. Next, the cells were washed with PBS three times, after which the anti-fluorescence

solution was added. The localized green fluorescence of the TUNEL-positive cells was visualized using an inverted fluorescence microscope (Olympus, Tokyo, Japan) and captured under an original magnification of 200×. The TUNEL-positive cells were counted using ImageJ.

Protein isolation and western blotting

22RV1 and LNCaP protein samples were collected using cold RIPA lysis buffer (Solarbio Life Sciences, Beijing, China). Protein concentrations were measured using the BCA technique (Solarbio Life Sciences, Beijing, China). After being boiled at 100°C for 5 min, the protein samples were separated on 10% SDS–polyacrylamide gels (140 V) and transferred to PVDF membranes (350 mA). The membranes were blocked with 5% skim milk in TBST for 1 h and then treated with antibodies against the proteins overnight at 4°C, followed by incubation with secondary antibodies and dilution with secondary antibodies at 37°C for 1 h. The immunobands were visualized using enhanced chemiluminescence (ECL) reagents (TransGen Biotechnology, Beijing, China) on a MicroChemi Chemiluminescent Imaging System (DNR Bio-Imaging Systems, Mahale HaHamisha, Jerusalem, Israel). The densitometric values were calculated with ImageJ 1.46r software (Wayne Rasband, National Institutes of Health, Bethesda, MA, USA), and the ratios of the target protein to GAPDH were calculated for statistical analysis.

Nuclear/cytoplasmic fractionation

Nuclear/cytoplasmic fractionation was conducted using a Nuclear and Cytoplasmic Protein Extraction Kit (Beyotime, Shenzhen, Guangdong, China), following the manufacturer's instructions. The cells were washed with cold PBS and resuspended in buffer containing 1 mM DTT and 1 mM PMSF, before being incubated on ice for 15 minutes. The detergent was then added, and the cells were vortexed for 30 seconds at the highest speed. The nuclei and the supernatant (cytoplasm) were separated by centrifugation at 4°C. The nuclei were resuspended in buffer containing 1 mM DTT

and 1 mM PMSF, incubated on ice for 30 minutes, and vortexed with intermittent agitation. The nuclear extracts were collected by centrifugation at 14,000 × g for 10 minutes at 4°C.

Migration and invasion assays

Invasion and migration assays were conducted using a Transwell system (Corning). 22Rv-1 and LNCaP cells (1×10^5) were seeded in small chambers (Corning) with serum-free medium for the 48-hour migration assay. The bottom wells were then filled with 700 mL of medium containing 20% FBS. In a similar manner, the invasion assay was performed by seeding 1×10^5 cells in serum-free medium into Matrigel (BD Biosciences)-coated chambers and culturing for 48 hours, with L15 supplemented with 20% FBS in the bottom wells. The number of cells that invaded or migrated through the chambers (with or without Matrigel) was counted in ten randomly chosen visual fields, and images were taken using a Leica microscope.

Coimmunoprecipitation (Co-IP)

A Pierce Classic Magnetic IP/Co-IP Kit (Thermo Fisher Scientific, USA) was used for immunoprecipitation in accordance with the manufacturer's instructions. The cells were lysed in cold PierceTM IP lysis buffer after being rinsed with ice-cold PBS. Subsequently, the supernatant was collected and subjected to overnight incubation at 4°C with the corresponding primary antibody or negative control IgG. The immunoprecipitants were then separated and subjected to SDS–PAGE. To determine the sequence of circRNF19A-490aa, a gel (with the indicated weight; band at 50 kDa) was cut and subjected to LC–MS analysis.

Dual-luciferase reporter assay

The luciferase plasmids were constructed using the pGL4.75 (hRluc-CMV) vector. The sequences of Luc-empty-vector, Luc-circRNF19A-IRES-WT, Luc-circRNF19A-IRES-MUT, Luc-circRNF19A-IRES-DEL1 and Luc-circRNF19A-IRES-DEL2 are

listed in Table S6. Cells were transfected with empty pGL4.75-vector or the indicated pGL4.75-constructs. After 72 h of transfection, the luciferase activity of each group of engineered cells was measured using a dual-luciferase reporter kit (TransGene, China) and a Synergy HTX multimode microplate reader (BioTek) according to the manufacturer's instructions.

References

[1] Li B, Dewey CN. RSEM: accurate transcript quantification from RNA-Seq data with or without a reference genome. BMC Bioinformatics. 2011. 12: 323.

[2] Fan L, Zhang F, Xu S, et al. Histone demethylase JMJD1A promotes alternative splicing of AR variant 7 (AR-V7) in prostate cancer cells. Proc Natl Acad Sci U S A. 2018. 115(20): E4584-E4593.
