# Supplementary material for: Androgen-targeted hsa_circ_0085121 encodes a novel protein and improves the development of prostate cancer through facilitating the activity of PI3K/Akt/mTOR pathway and enhancing AR-V7 alternative splicing
Source: Cell Death Dis. 2024 Nov 20;15(11):848. doi: 10.1038/s41419-024-07246-9 (PMC11579034; doi:10.1038/s41419-024-07246-9)

# Original images of western blot gel for “Androgen-targeted hsa_circ_0085121 encodes a novel protein and improves the development of prostate cancer through facilitating the activity of PI3K/Akt/mTOR pathway and enhancing AR-V7 alternative splicing.”

# Figure 2G

PARP

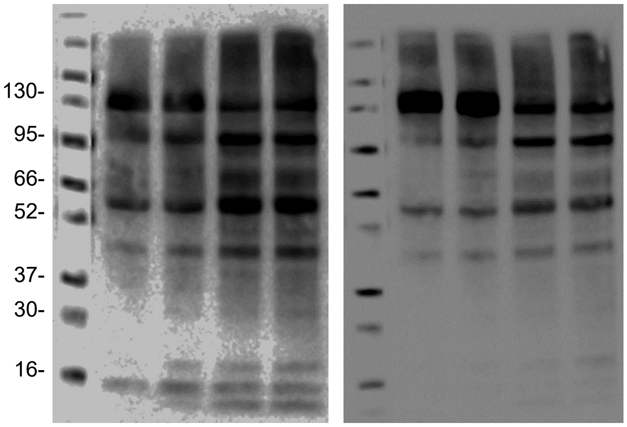


Caspase-3

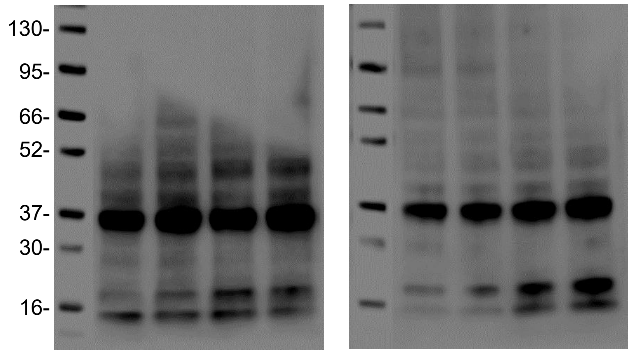


Bax

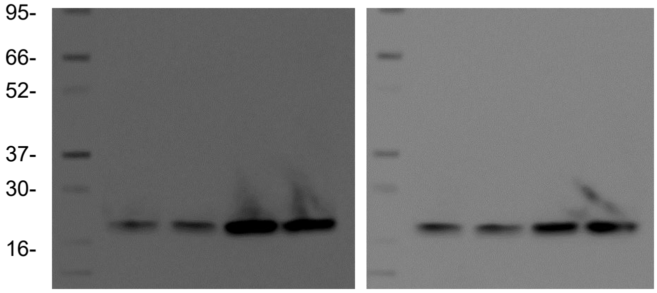


Bcl-2

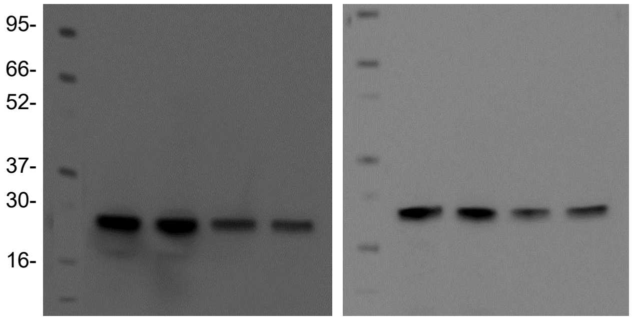


B-actin

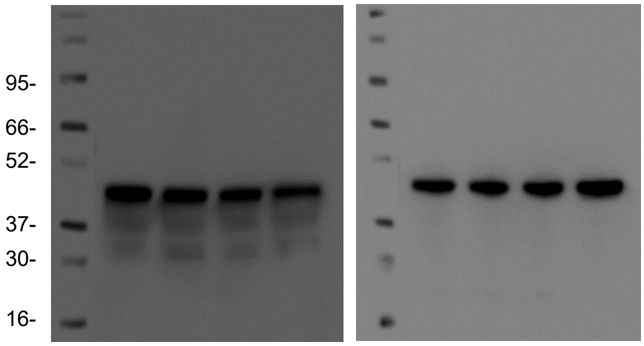

# Figure 3H

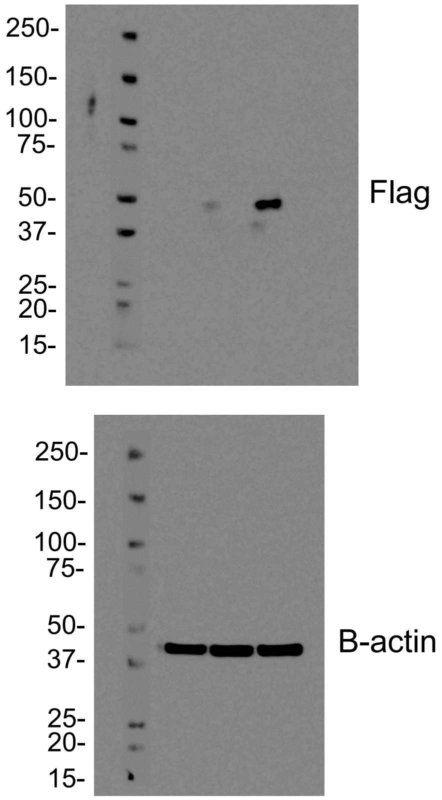


# Figure 3I

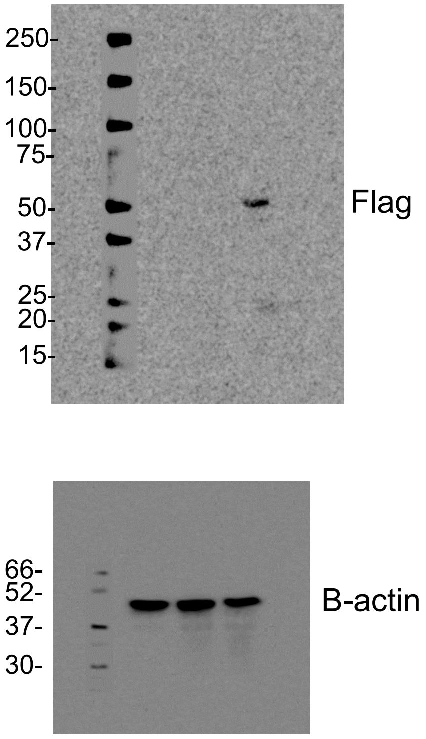


# Figure 3N

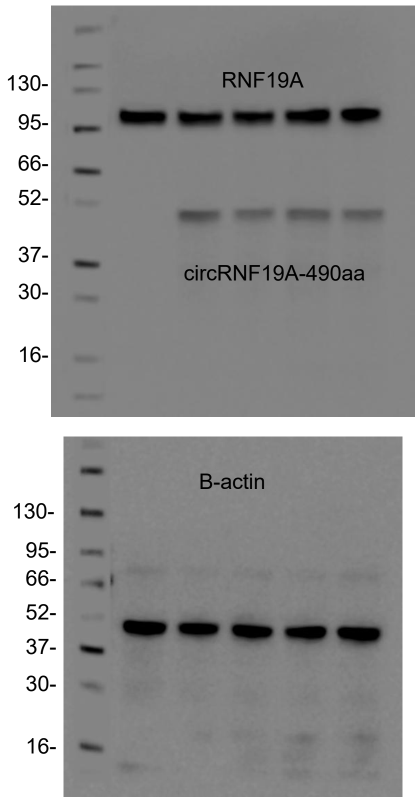


# Figure 3M

Upper panel

Lower panel

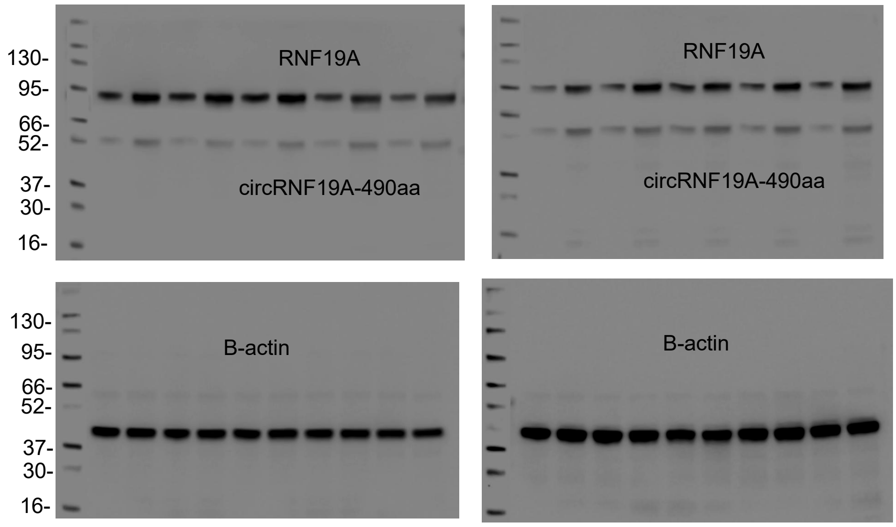


# Figure 3P

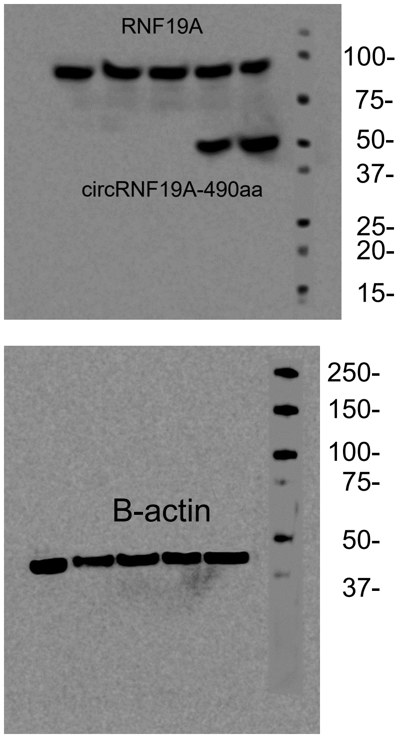

# Figure 3Q

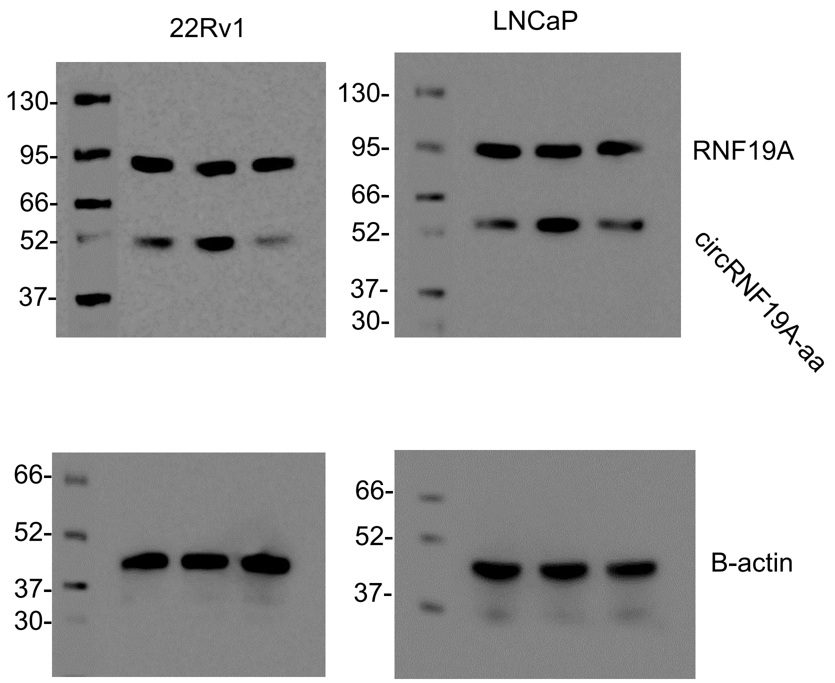


# Figure 3R

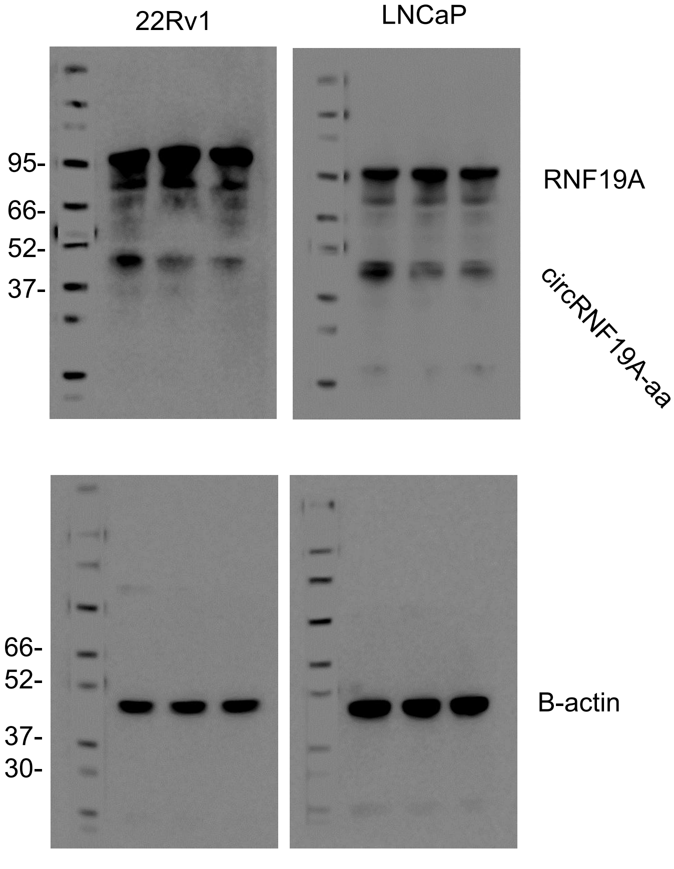

# Figure 4G

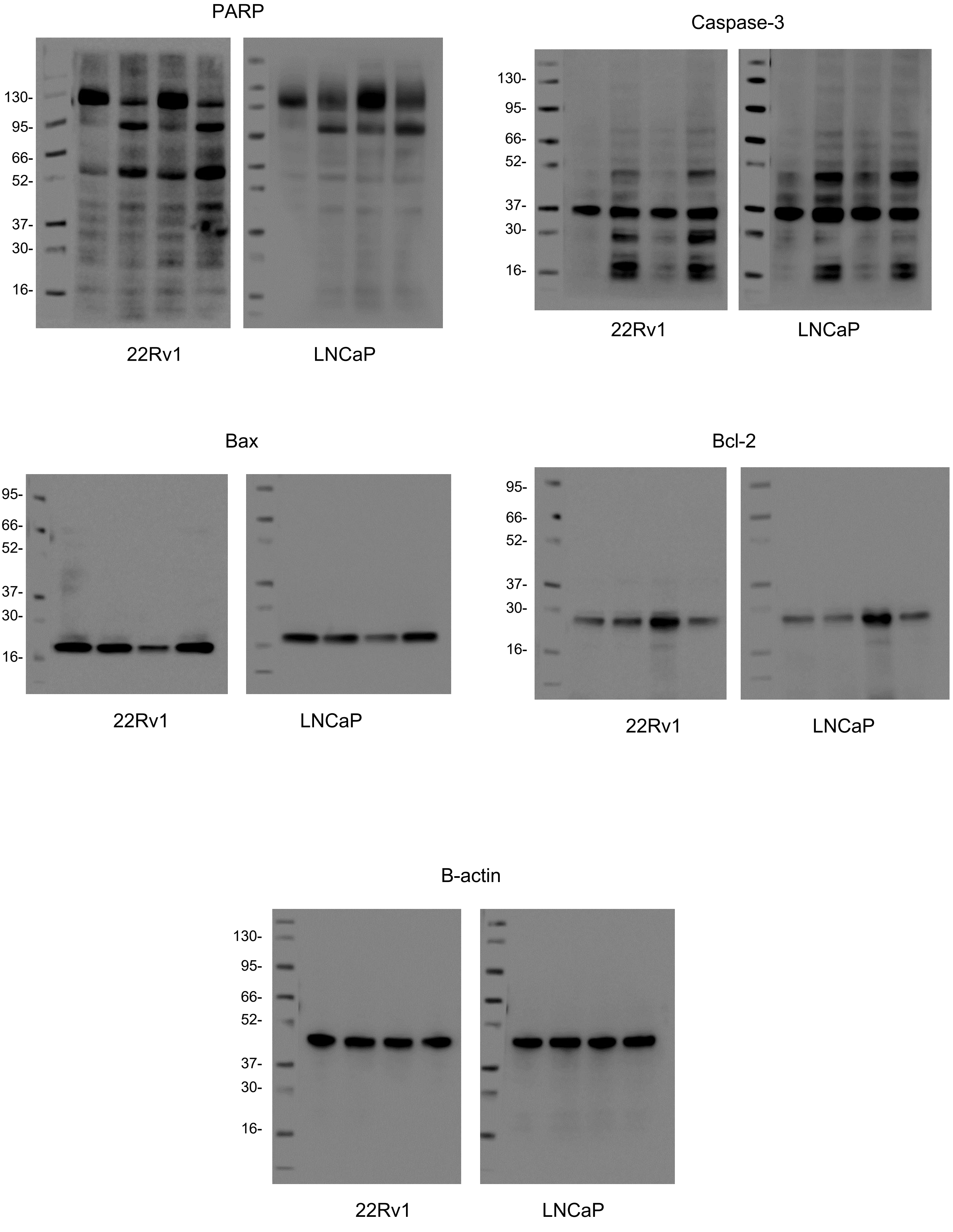

PARP
130-
95-
66-
52-
37-
30-
16-
22Rv1
LNCaP
Caspase-3
130-
95-
66-
52-
37-
30-
16-
22Rv1
LNCaP
Bax
95-
66-
52-
37-
30-
16-
22Rv1
LNCaP
Bcl-2
95-
66-
52-
37-
30-
16-
22Rv1
LNCaP
B-actin
130-
95-
66-
52-
37-
30-
16-
22Rv1
LNCaP

# Figure 4O

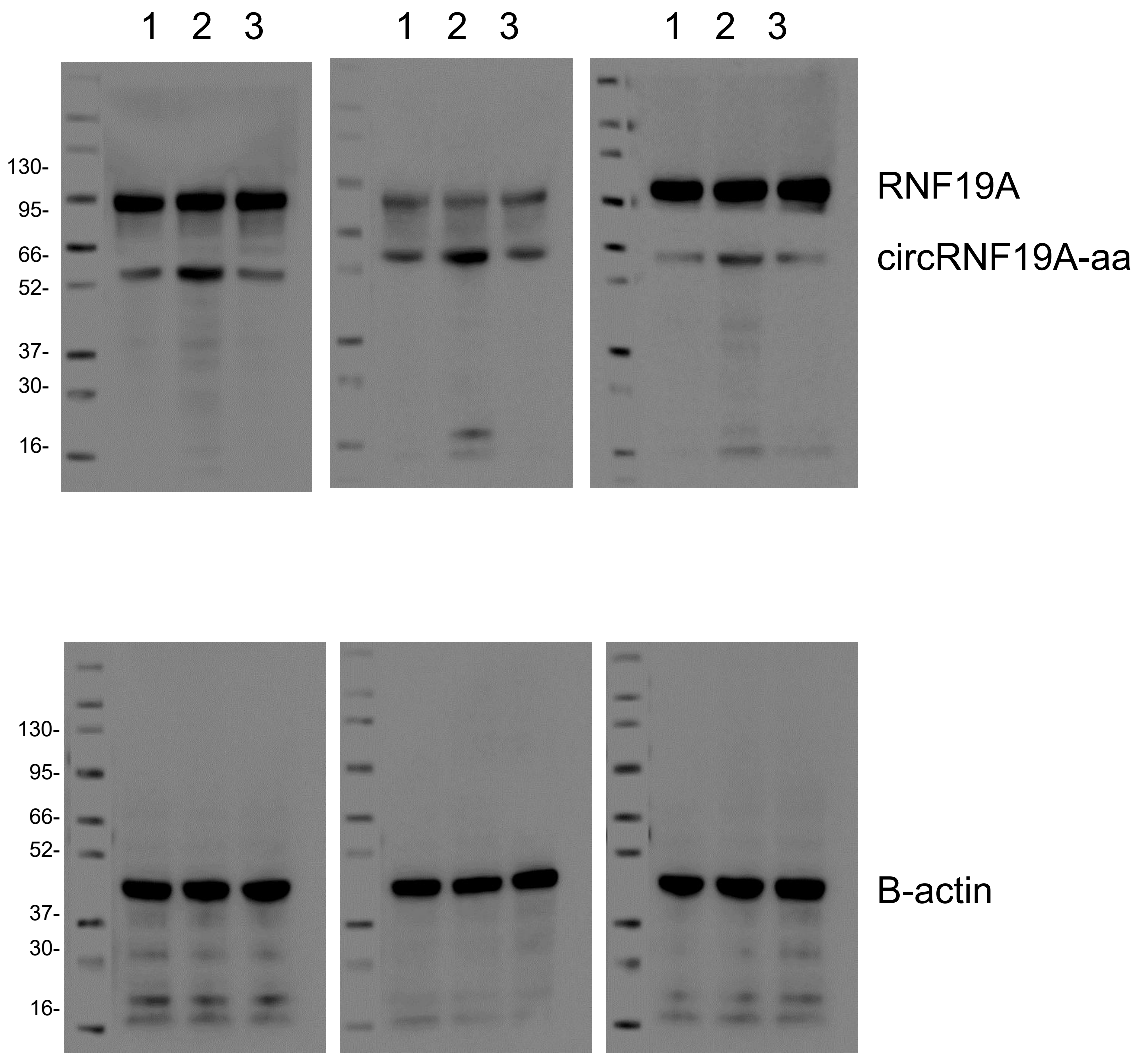

1 2 3
1 2 3
1 2 3
130-
95-
66-
52-
37-
30-
16-
RNF19A
circRNF19A-aa
130-
95-
66-
52-
37-
30-
16-
B-actin

# Figure 5A

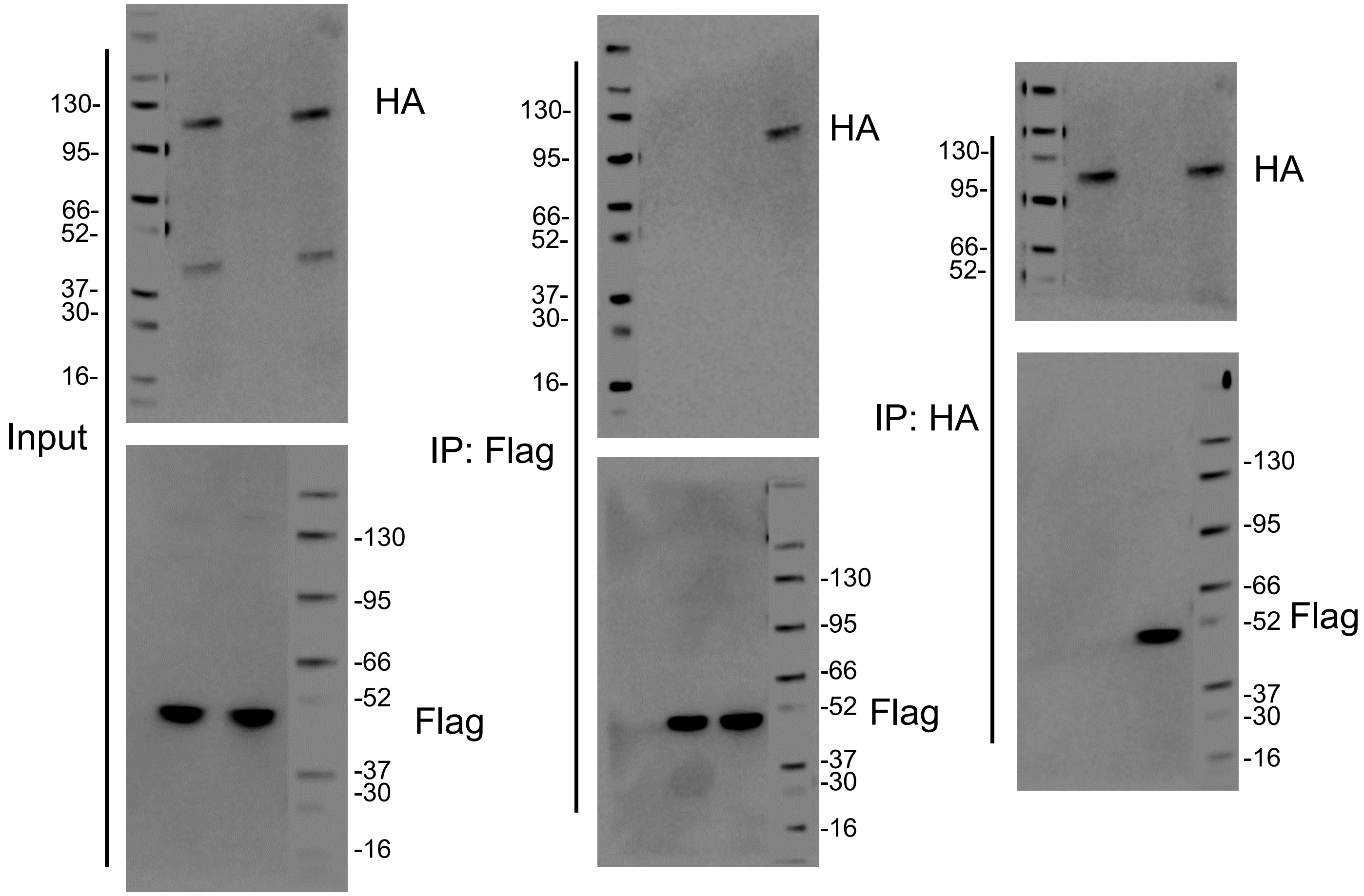


# Figure 5B

22Rv1

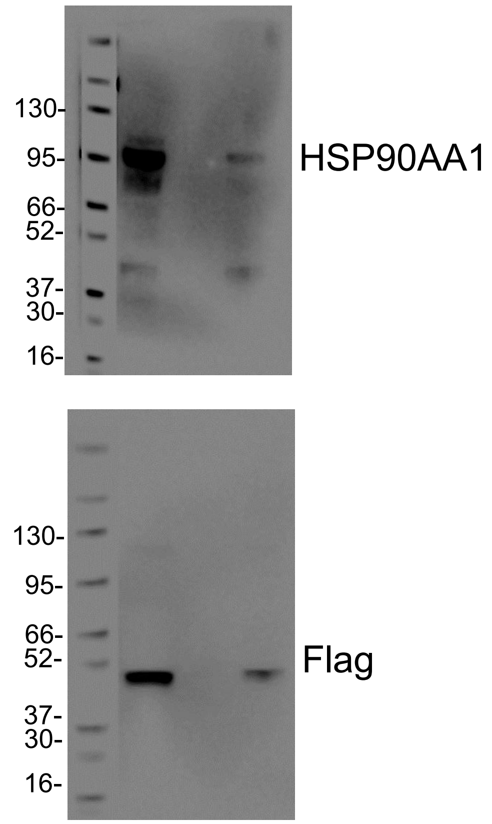


# Figure 5C

LNCaP

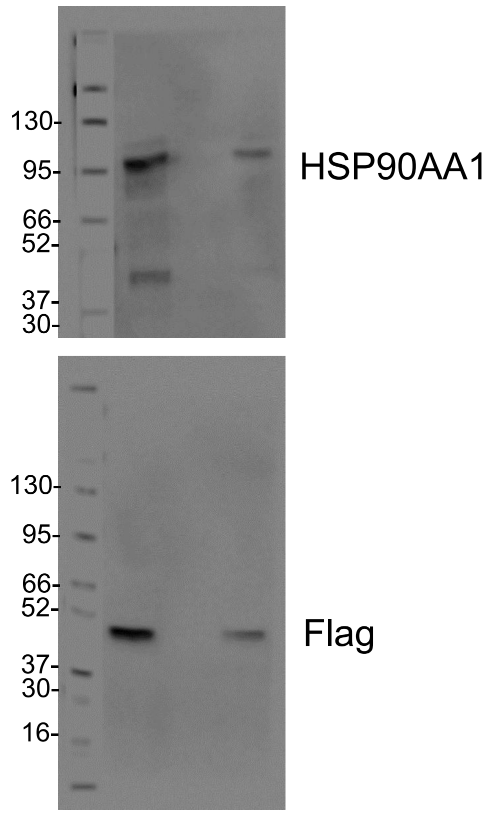

Figure 5D

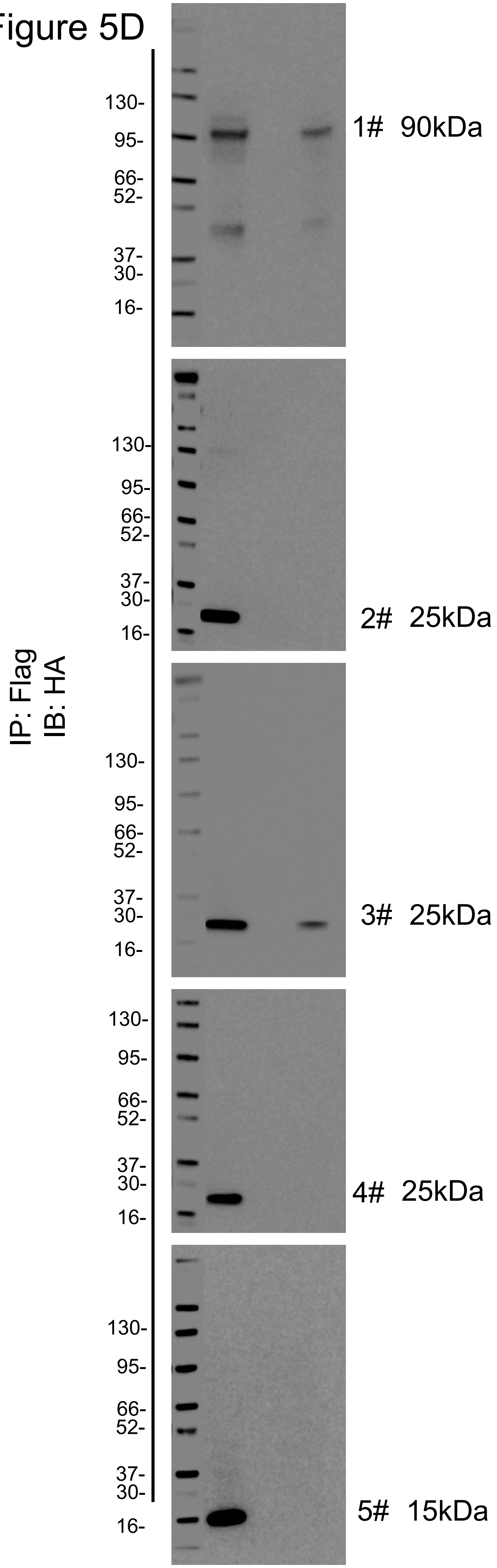


Figure 5E

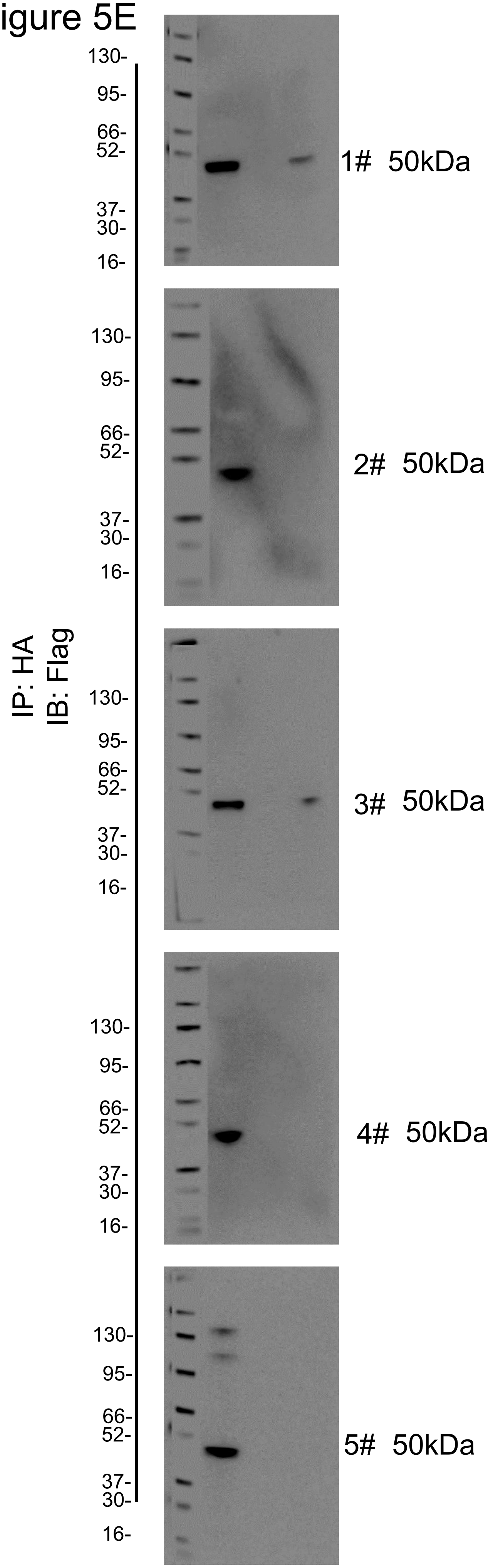

Figure 5G

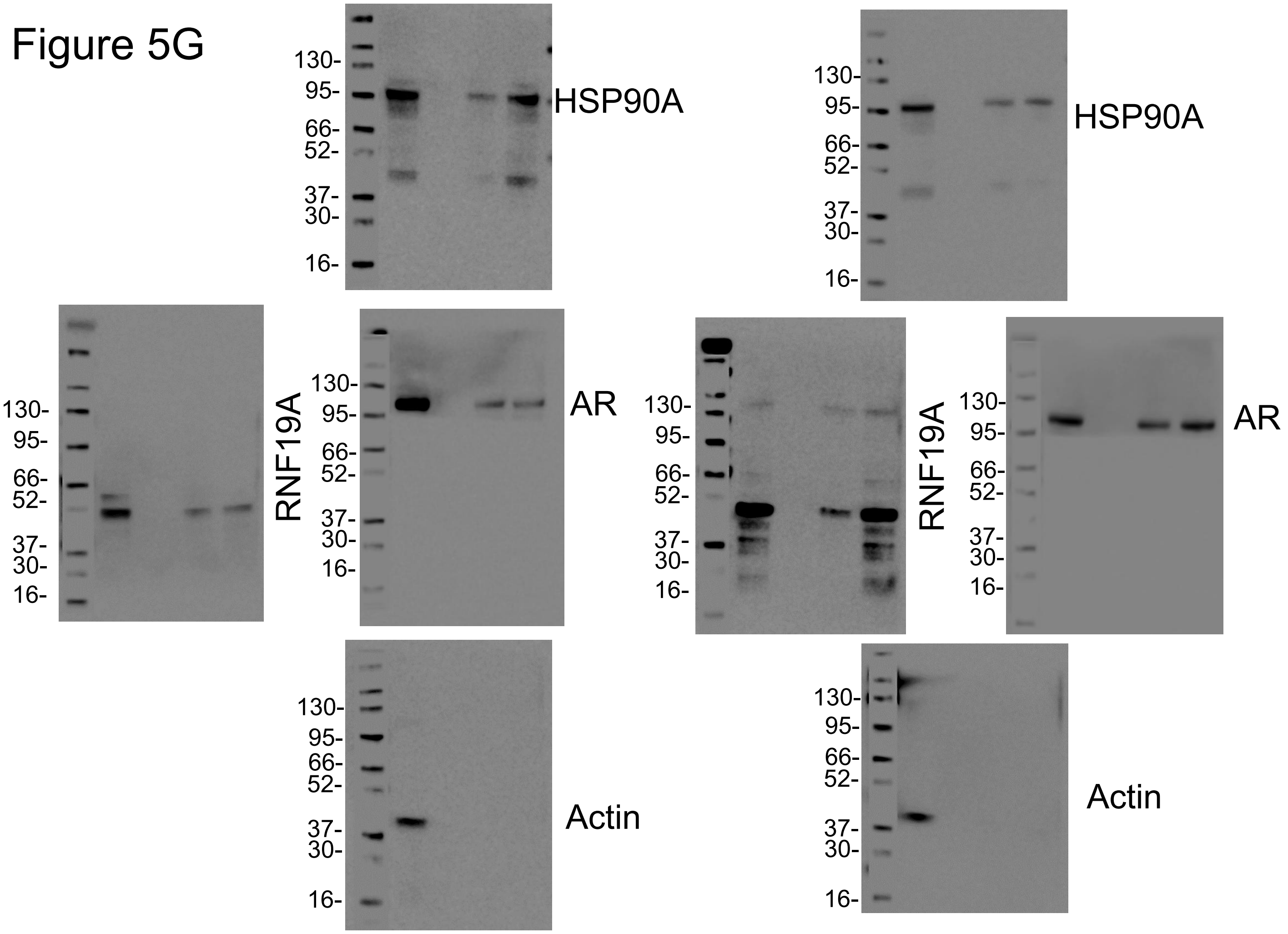


Figure 5H

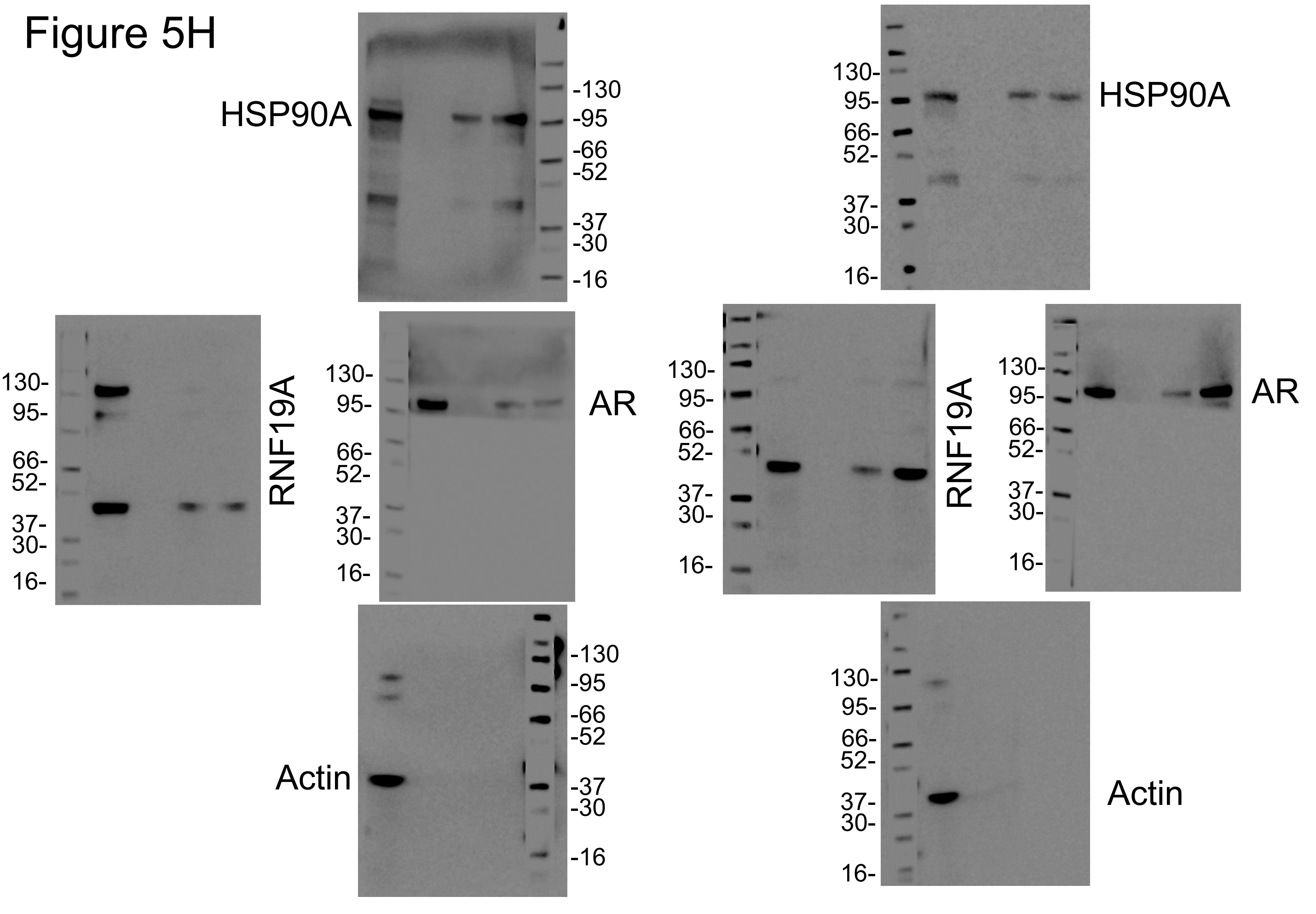

# Figure 5J

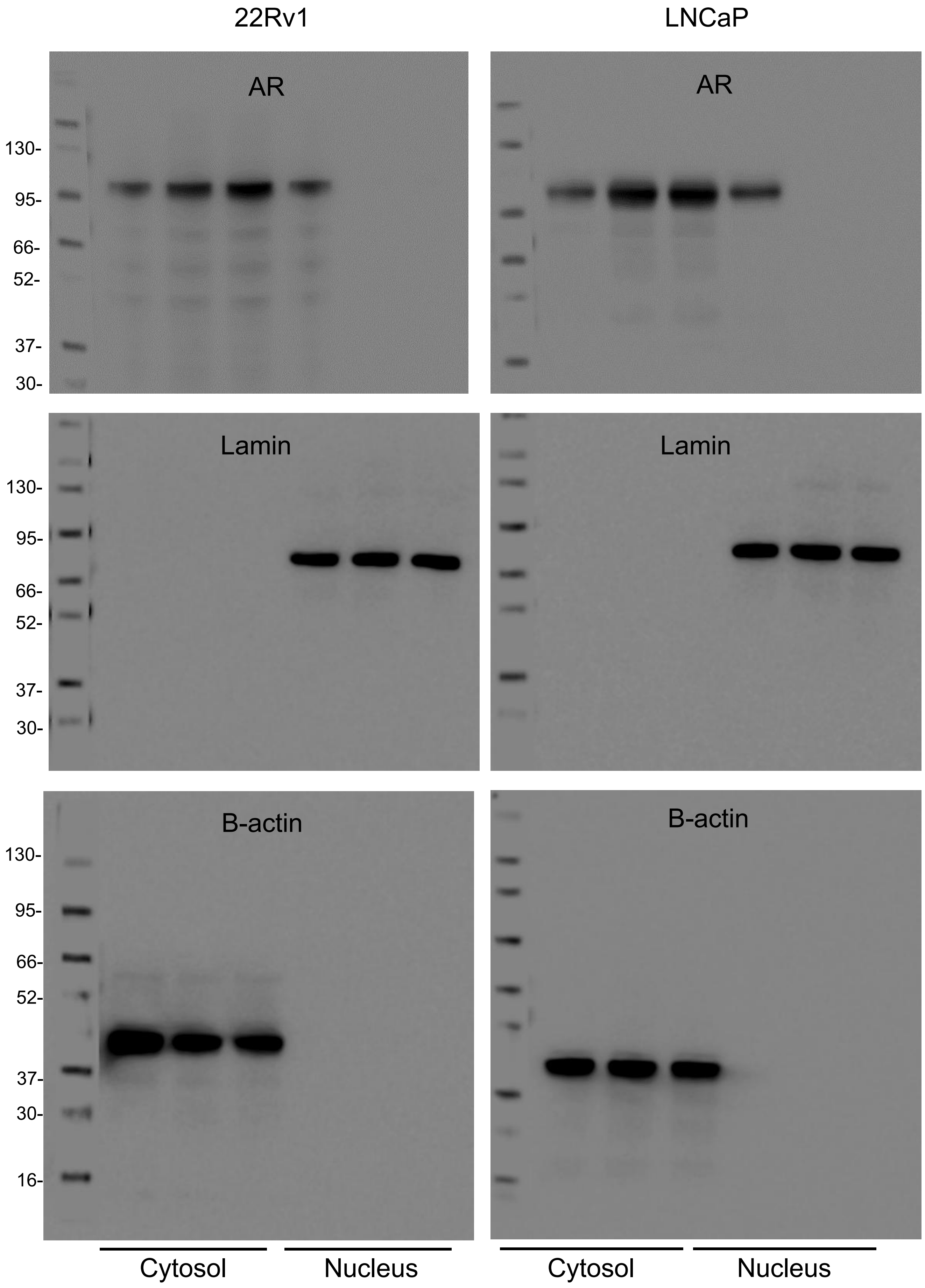
22Rv1
LNCaP
AR
Lamin
B-actin
130-
95-
66-
52-
37-
30-
16-
Cytosol
Nucleus

# Figure 5N

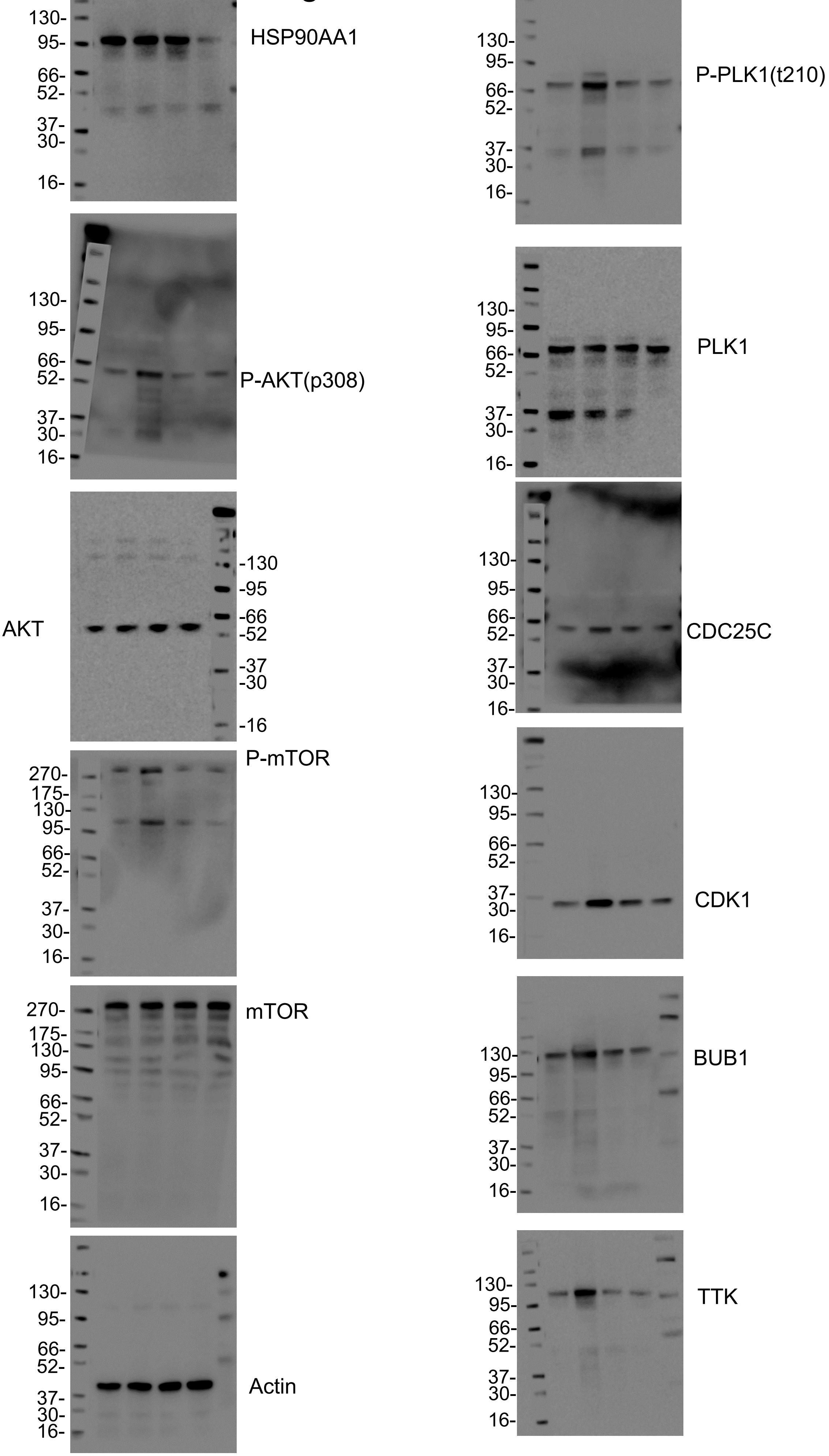
HSP90AA1
P-AKT(p308)
AKT
P-mTOR
mTOR
Actin
P-PLK1(t210)
PLK1
CDC25C
CDK1
BUB1
TTK
270-
175-
130-
95-
66-
52-
37-
30-
16-

# Figure 5O

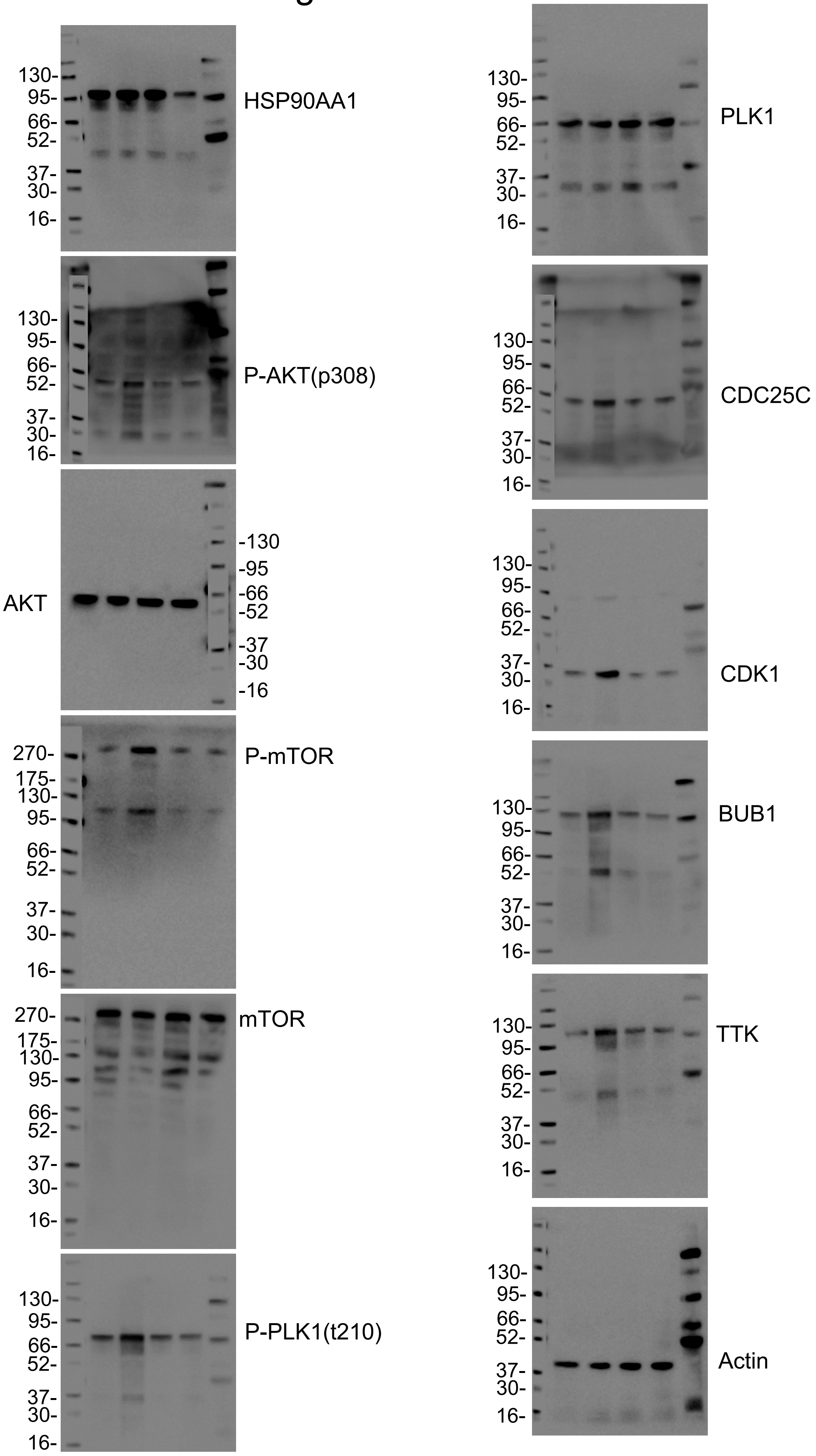

HSP90AA1
130-
95-
66-
52-
37-
30-
16-
P-AKT(p308)
AKT
-130
-95
-66
-52
-37
-30
-16
P-mTOR
270-
175-
mTOR
P-PLK1(t210)
PLK1
CDC25C
CDK1
BUB1
TTK
Actin

# Figure 6G

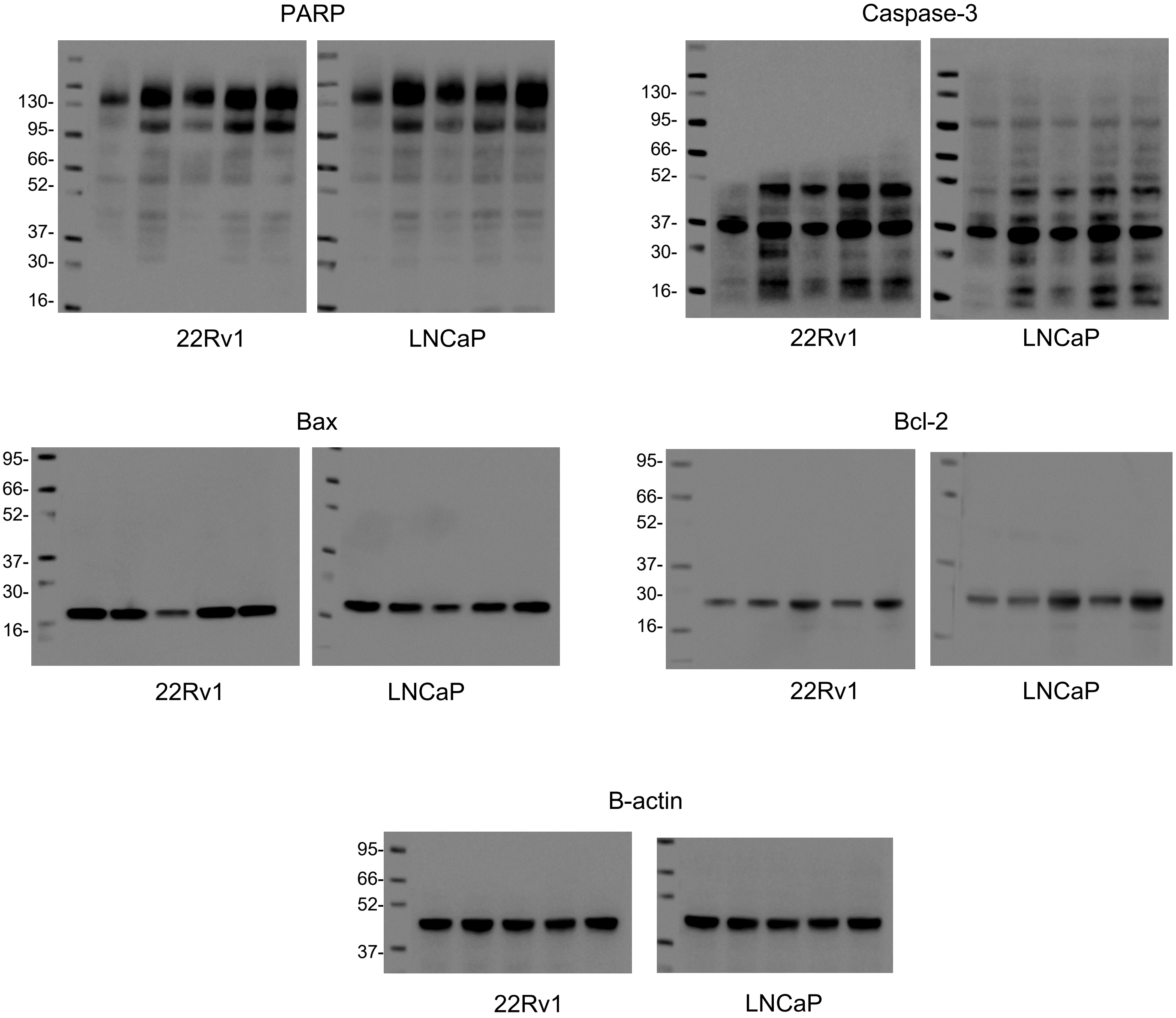


# Figure 7A

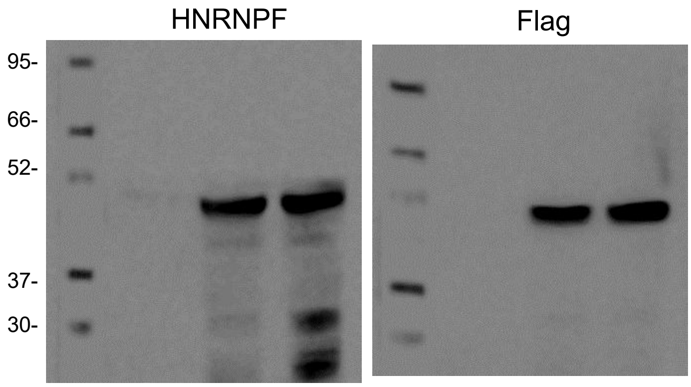


# Figure 7B

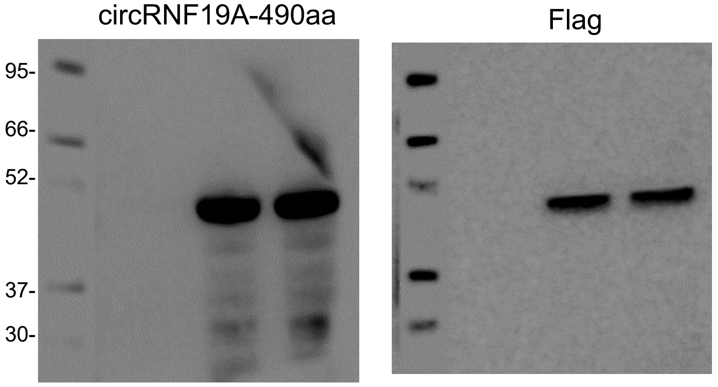

Figure 7C

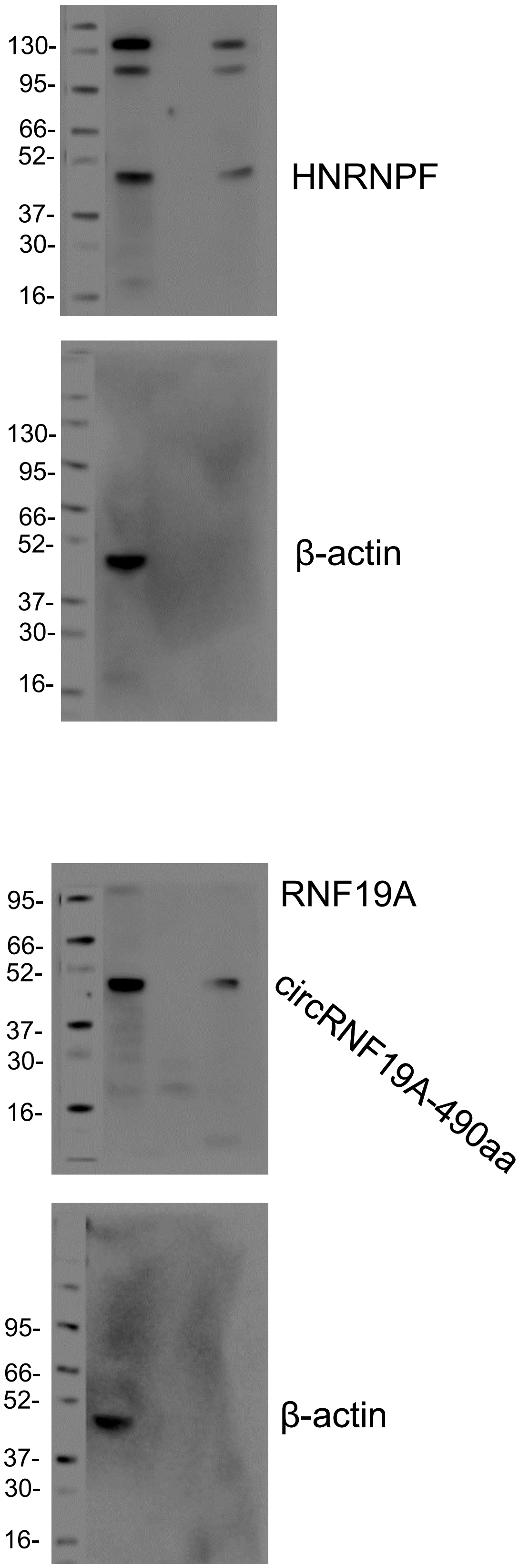


Figure 7D

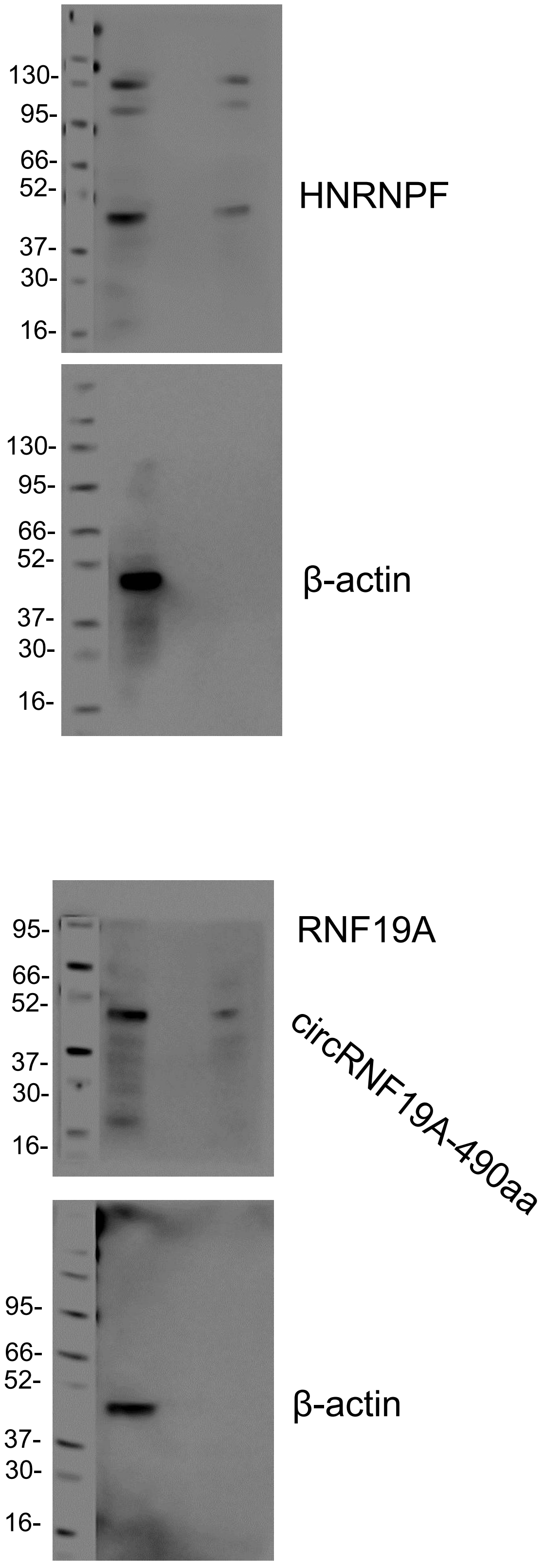

# Figure 7E

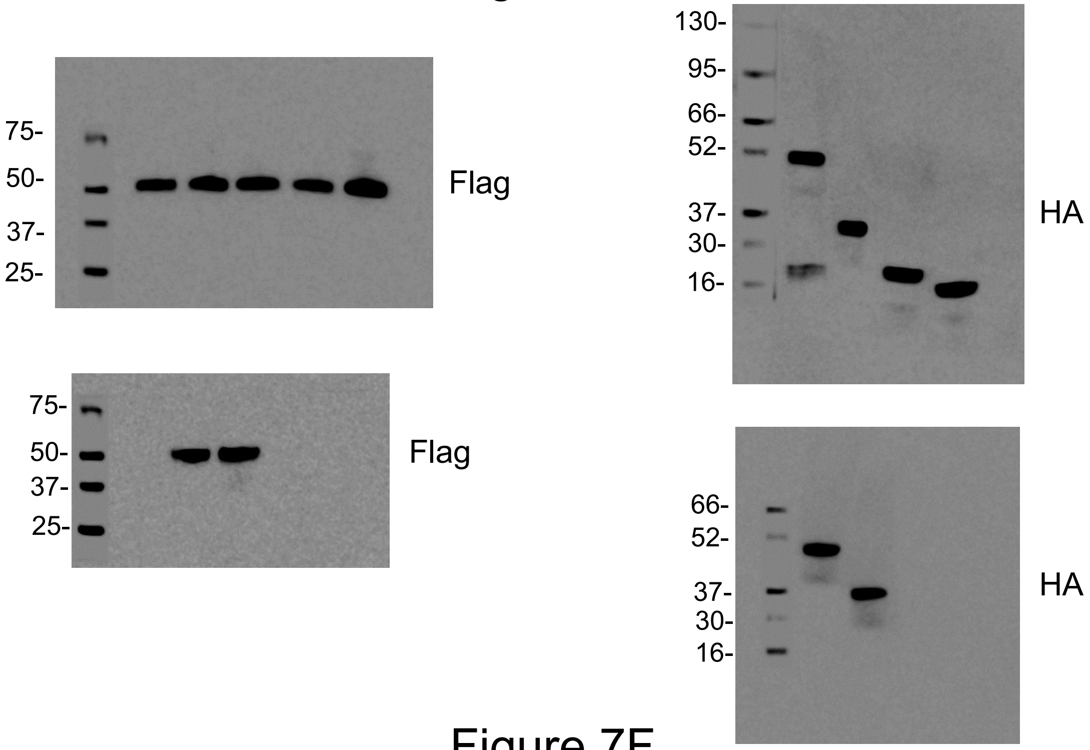


# Figure 7F

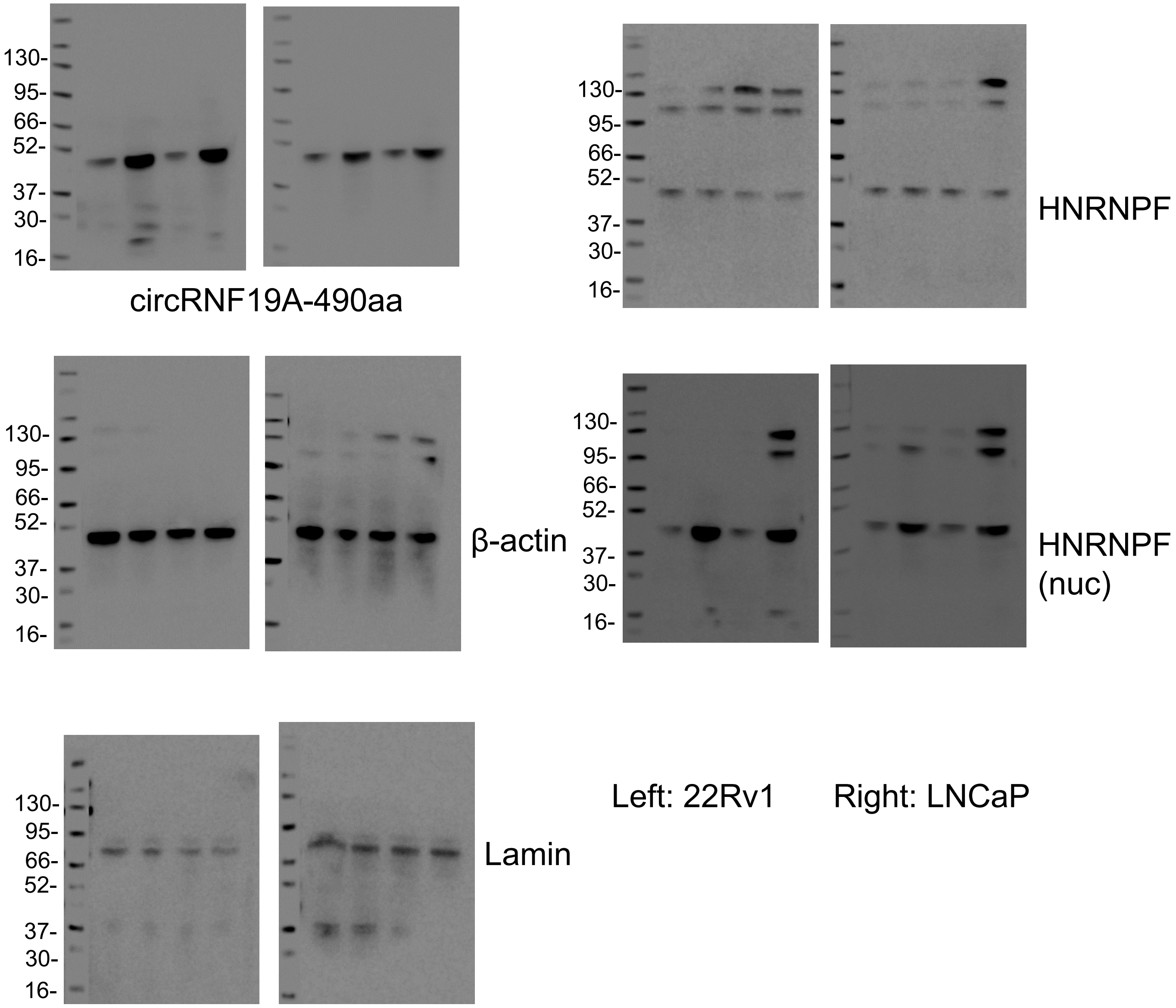

# Figure 7H (upper)

# Figure 7H (lower)

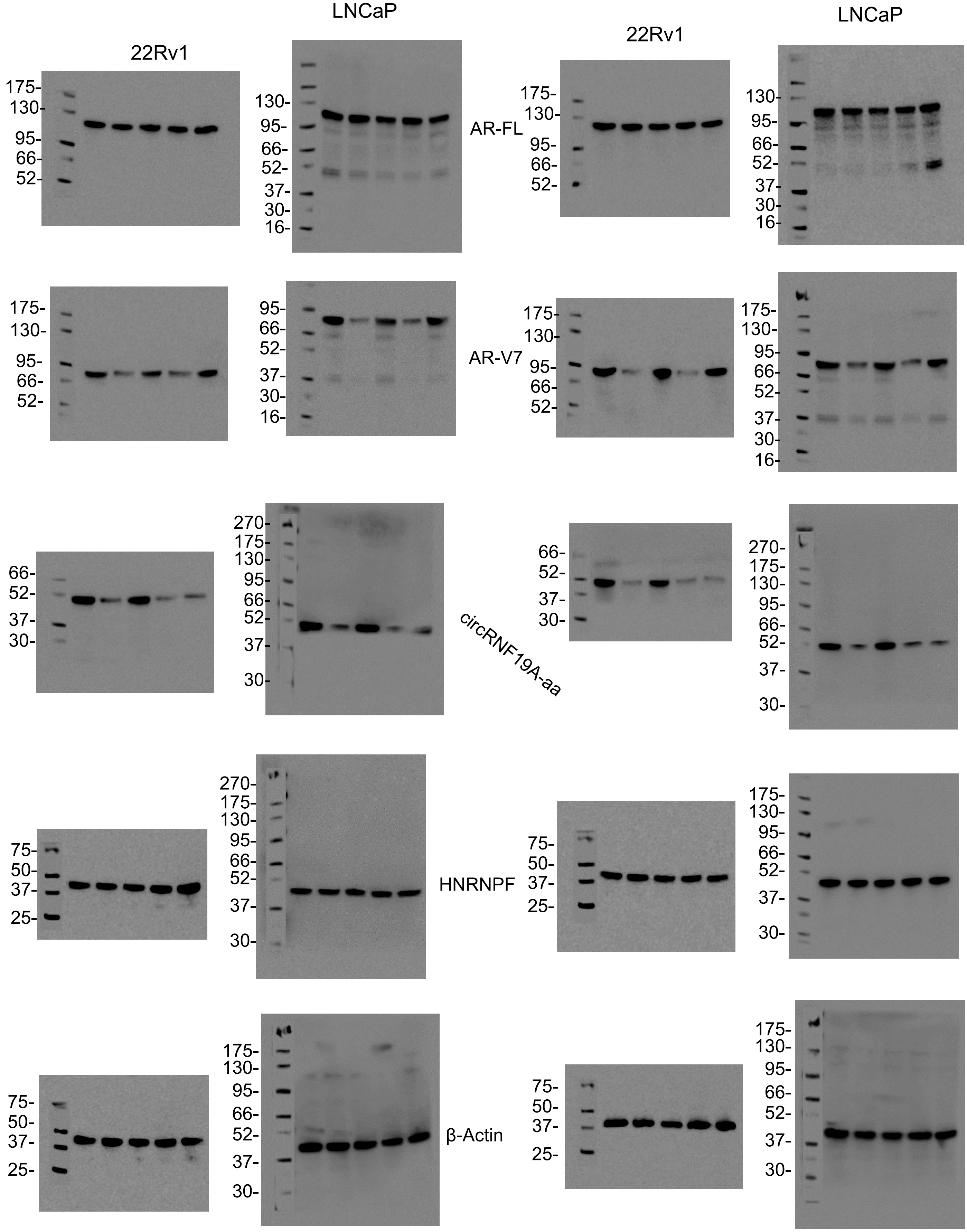

# Figure 8B

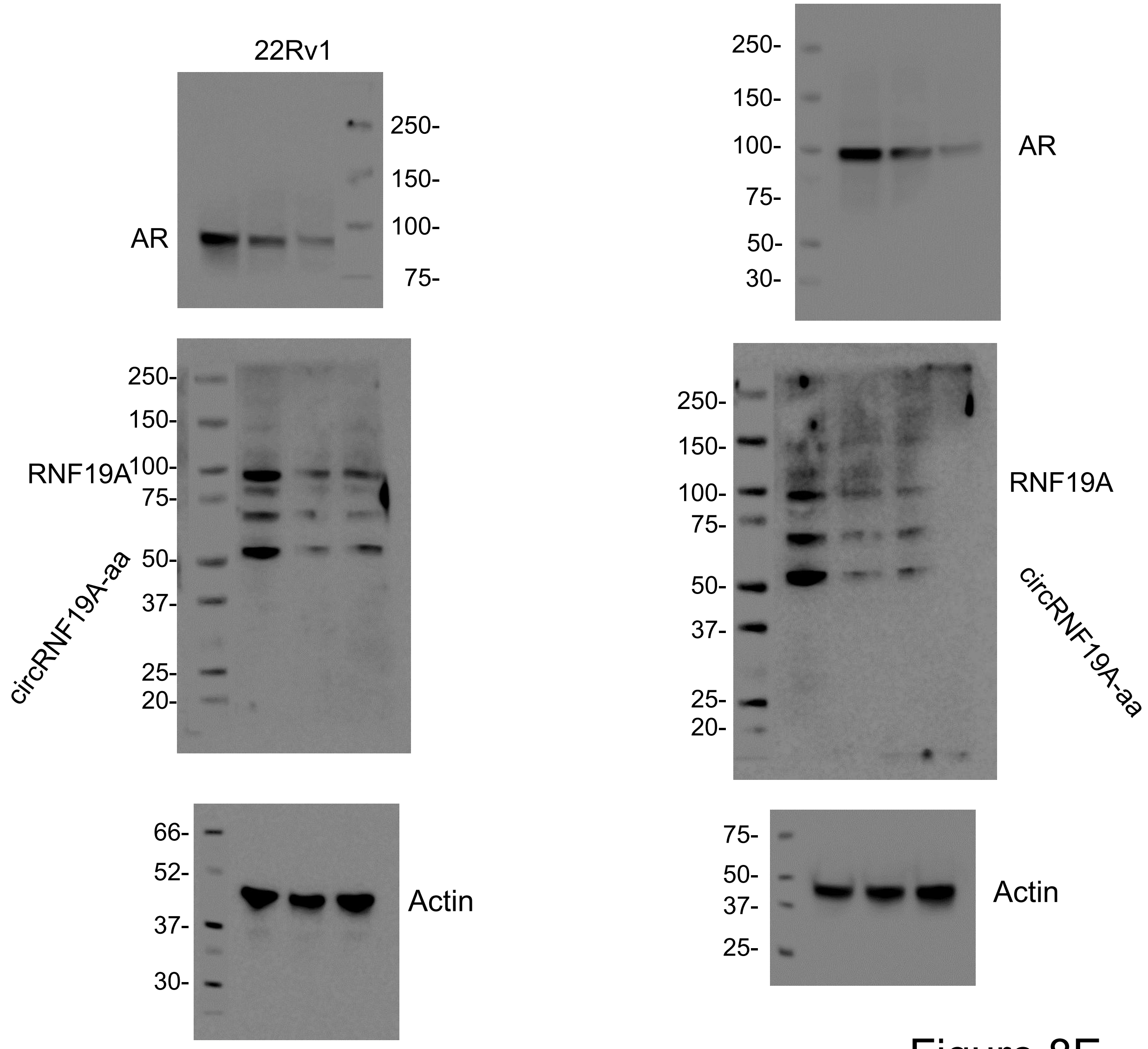


# Figure 8D

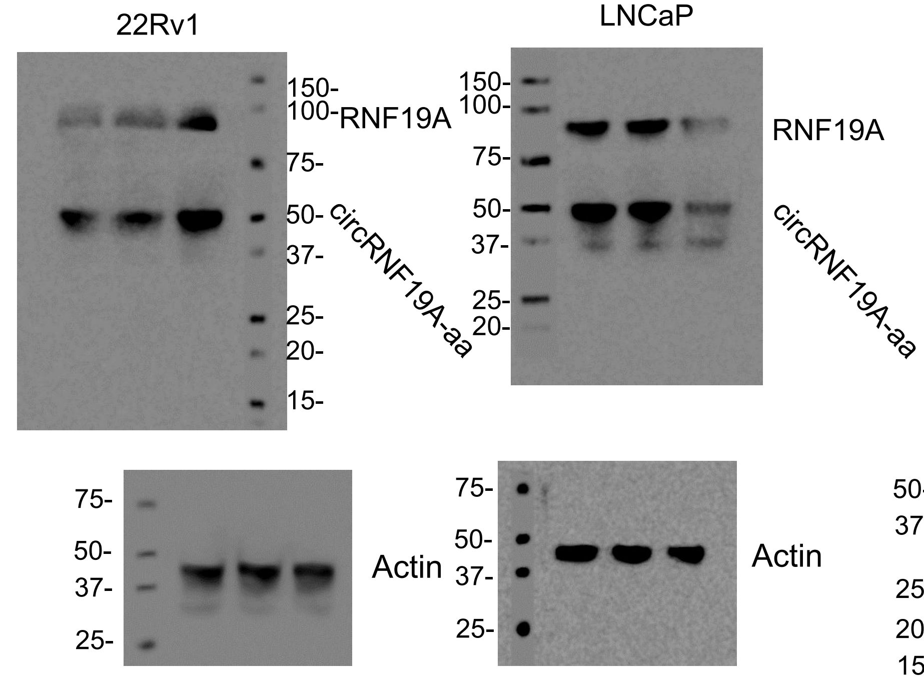


# Figure 8E

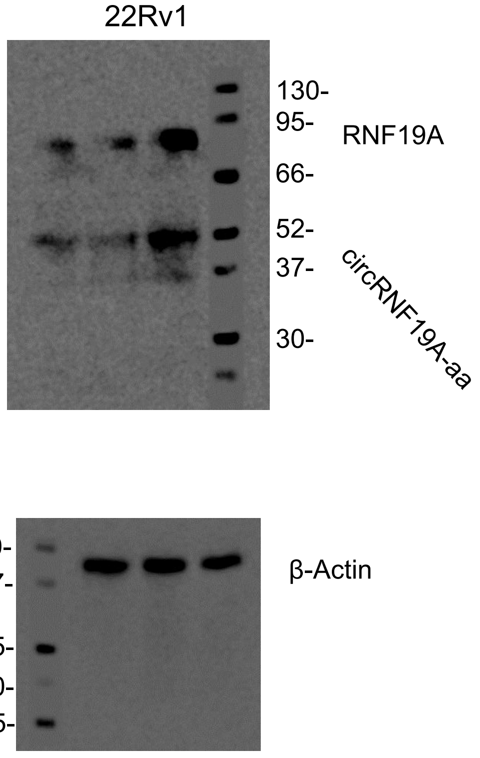

# Figure 8E

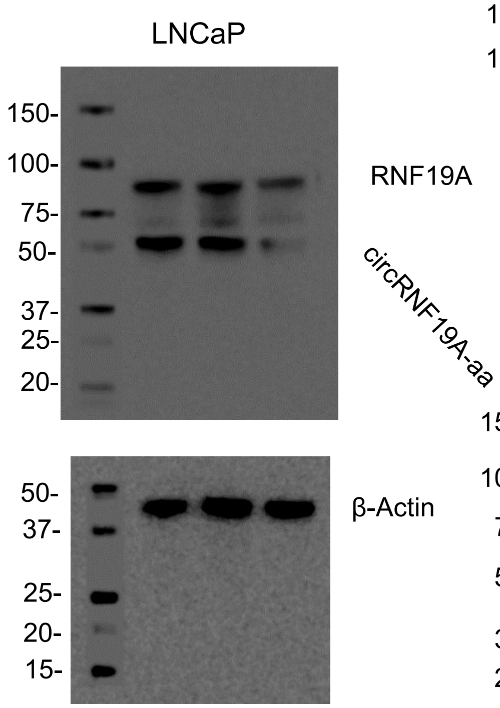


# Figure 8K

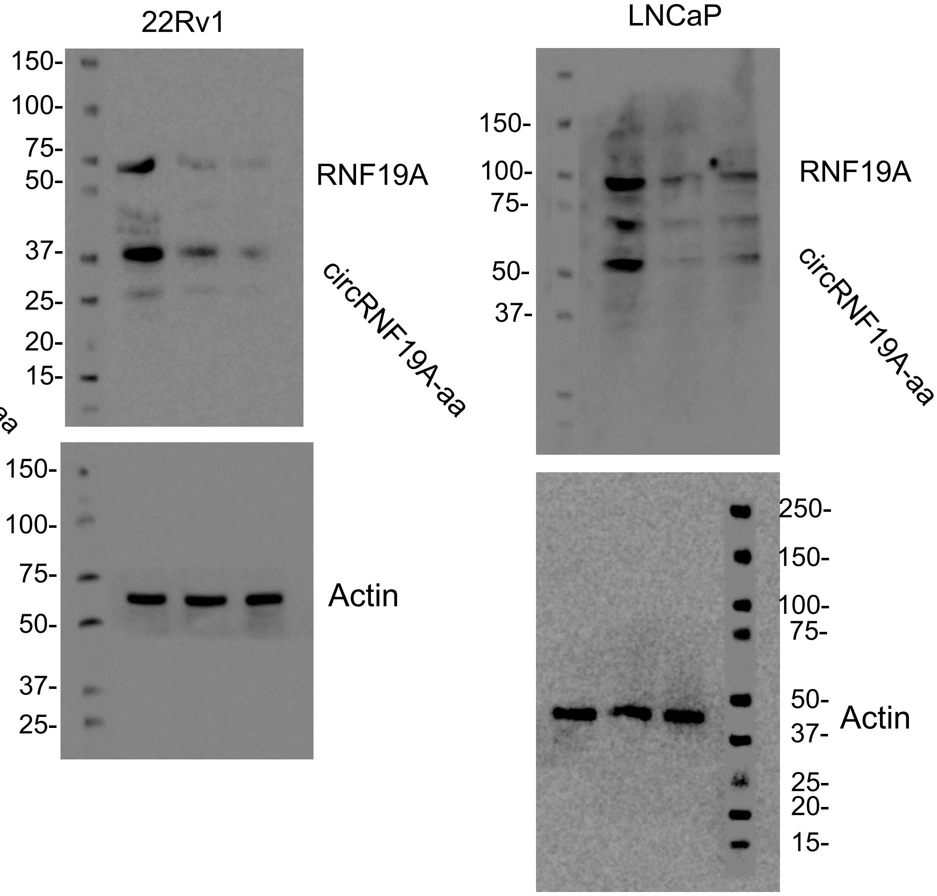


# Figure 8N

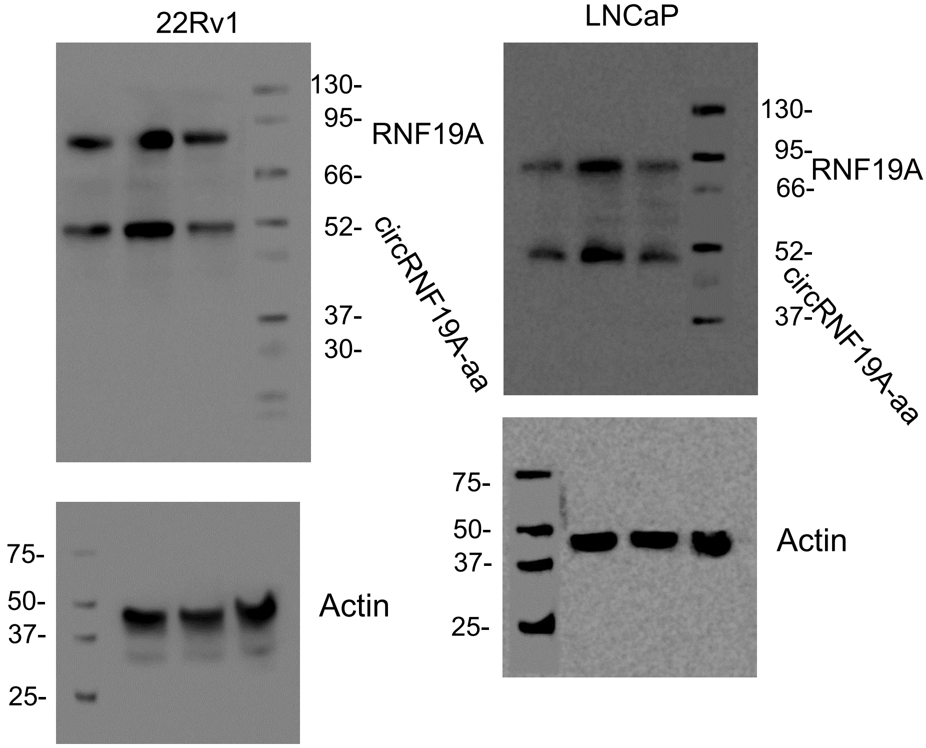

# Figure S5J

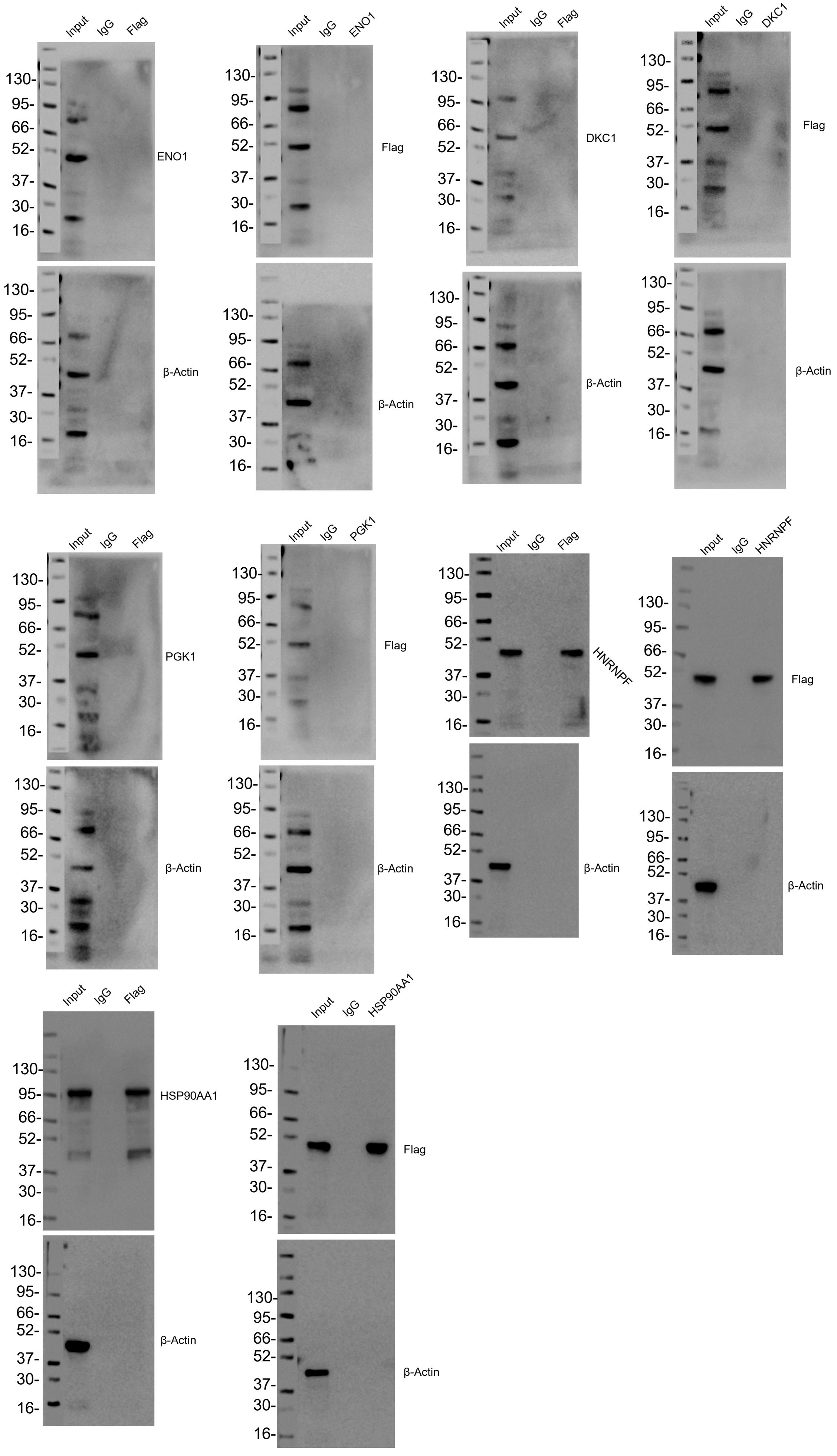
Input IgG Flag
ENO1
β-Actin
Input IgG ENO1
Flag
β-Actin
Input IgG Flag
DKC1
β-Actin
Input IgG DKC1
Flag
β-Actin
Input IgG Flag
PGK1
β-Actin
Input IgG PGK1
Flag
β-Actin
Input IgG Flag
HNRNPF
β-Actin
Input IgG HNRNPF
Flag
β-Actin
Input IgG Flag
HSP90AA1
β-Actin
Input IgG HSP90AA1
Flag
β-Actin
130- 95- 66- 52- 37- 30- 16-

## Figure S6D (22Rv1)

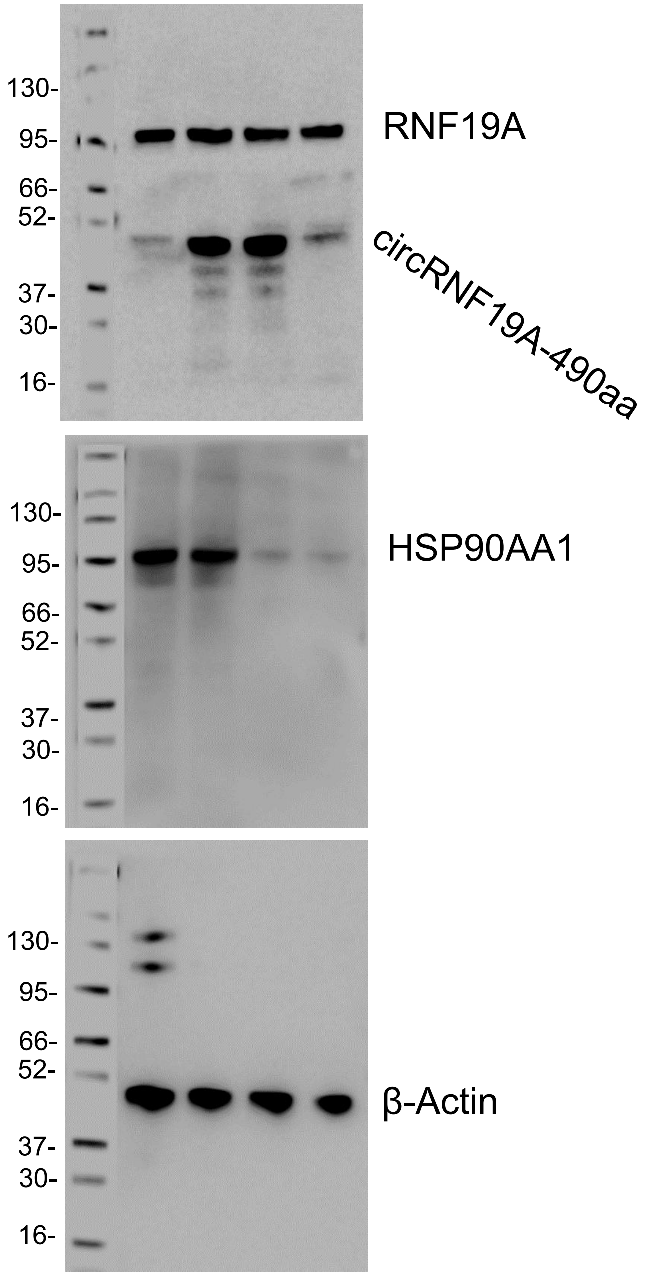


## Figure S6D (LNCaP)

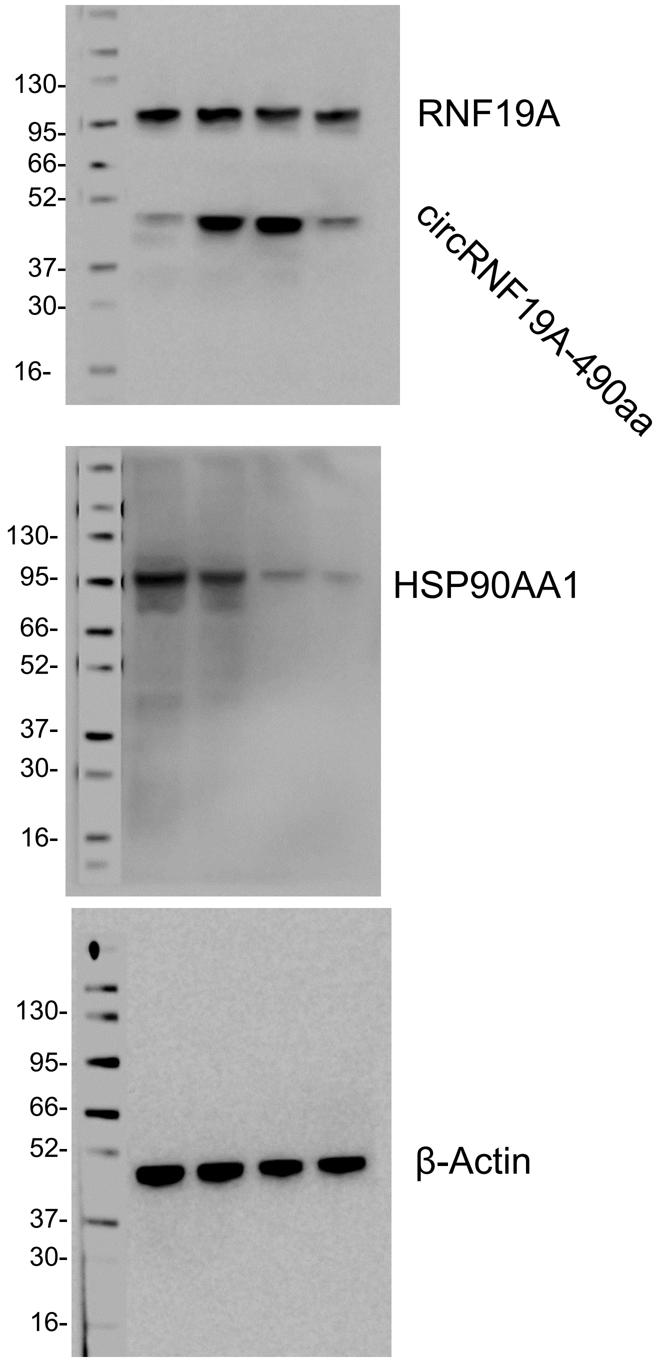

# Figure S6F

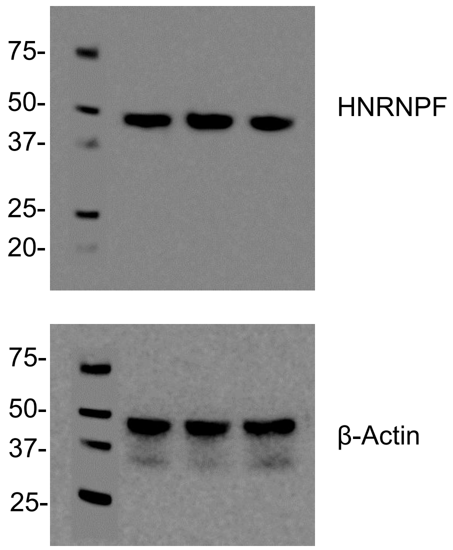


# Figure S6P (Upper)

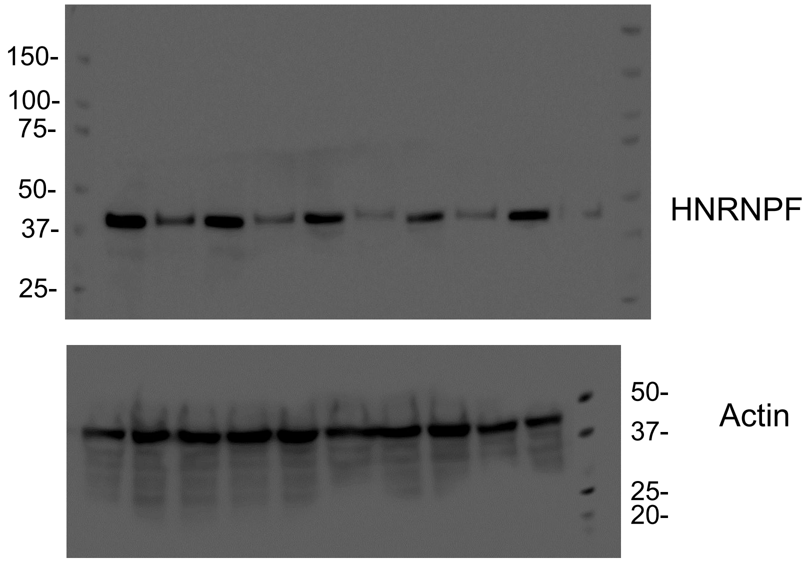


# Figure S6P (Lower)

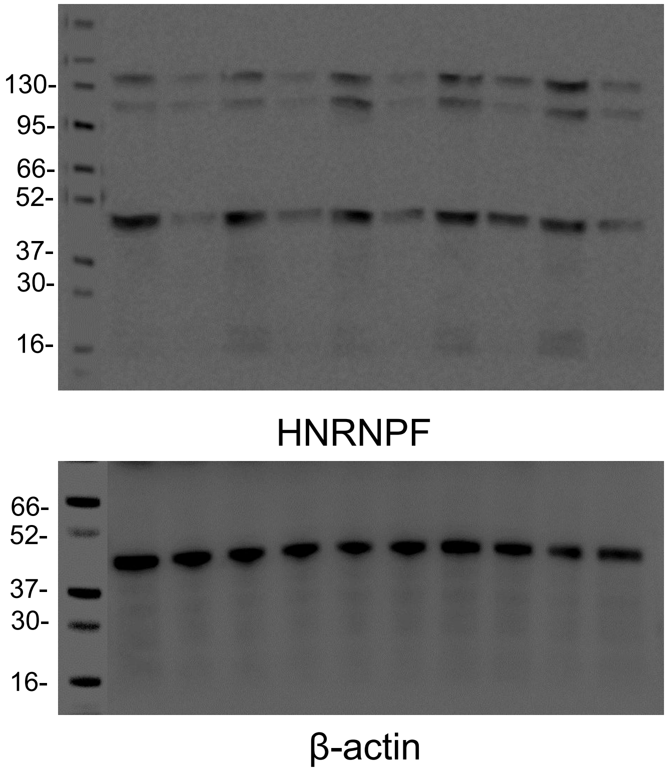


# Figure S6R

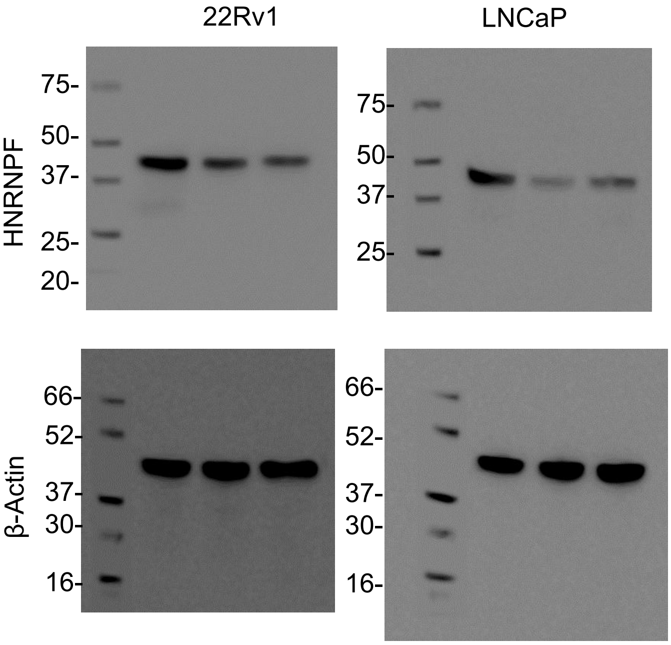

# Figure S7G

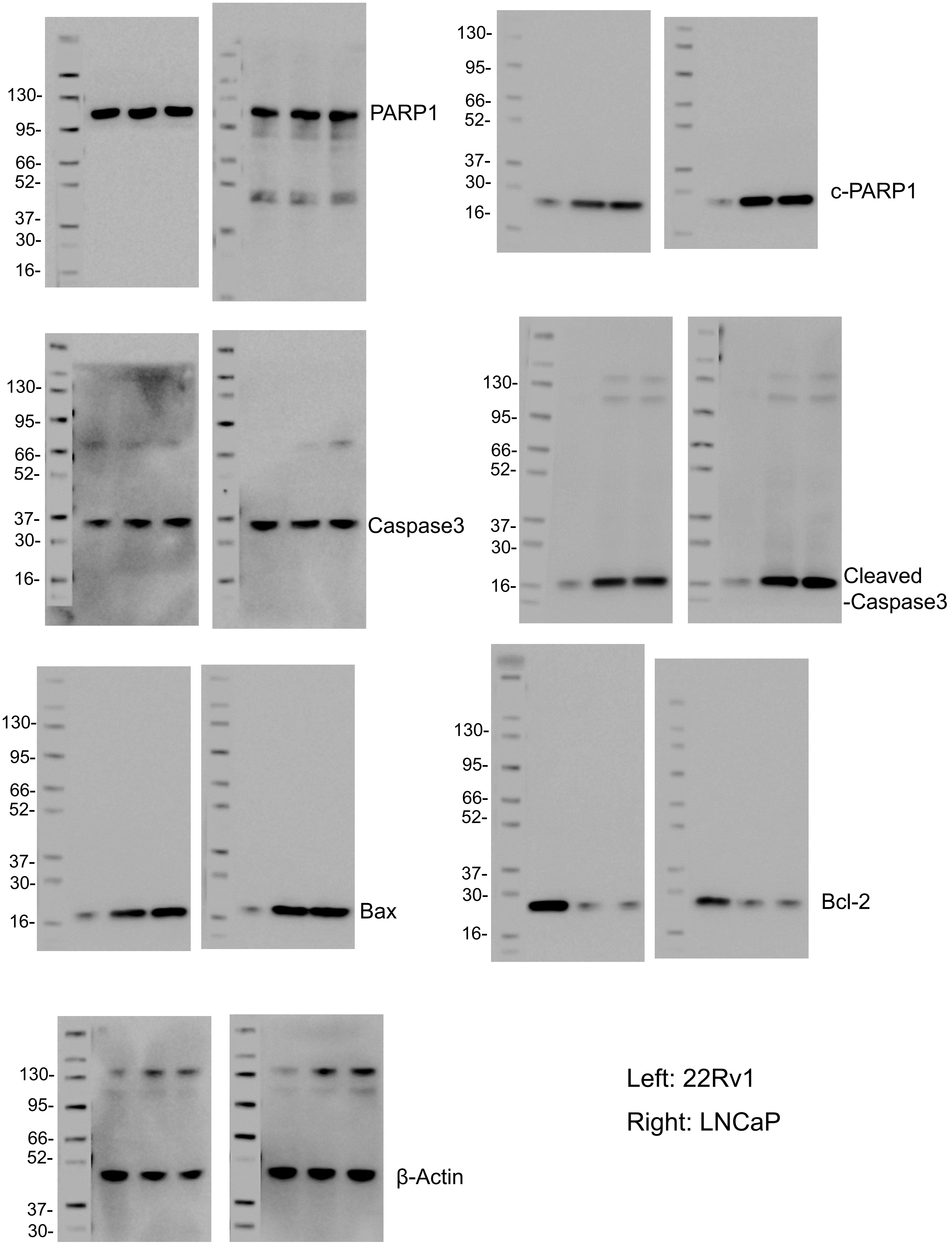
PARP1
c-PARP1
Caspase3
Cleaved
-Caspase3
Bax
Bcl-2
β-Actin
130-
95-
66-
52-
37-
30-
16-
Left: 22Rv1
Right: LNCaP

# Figure S7G

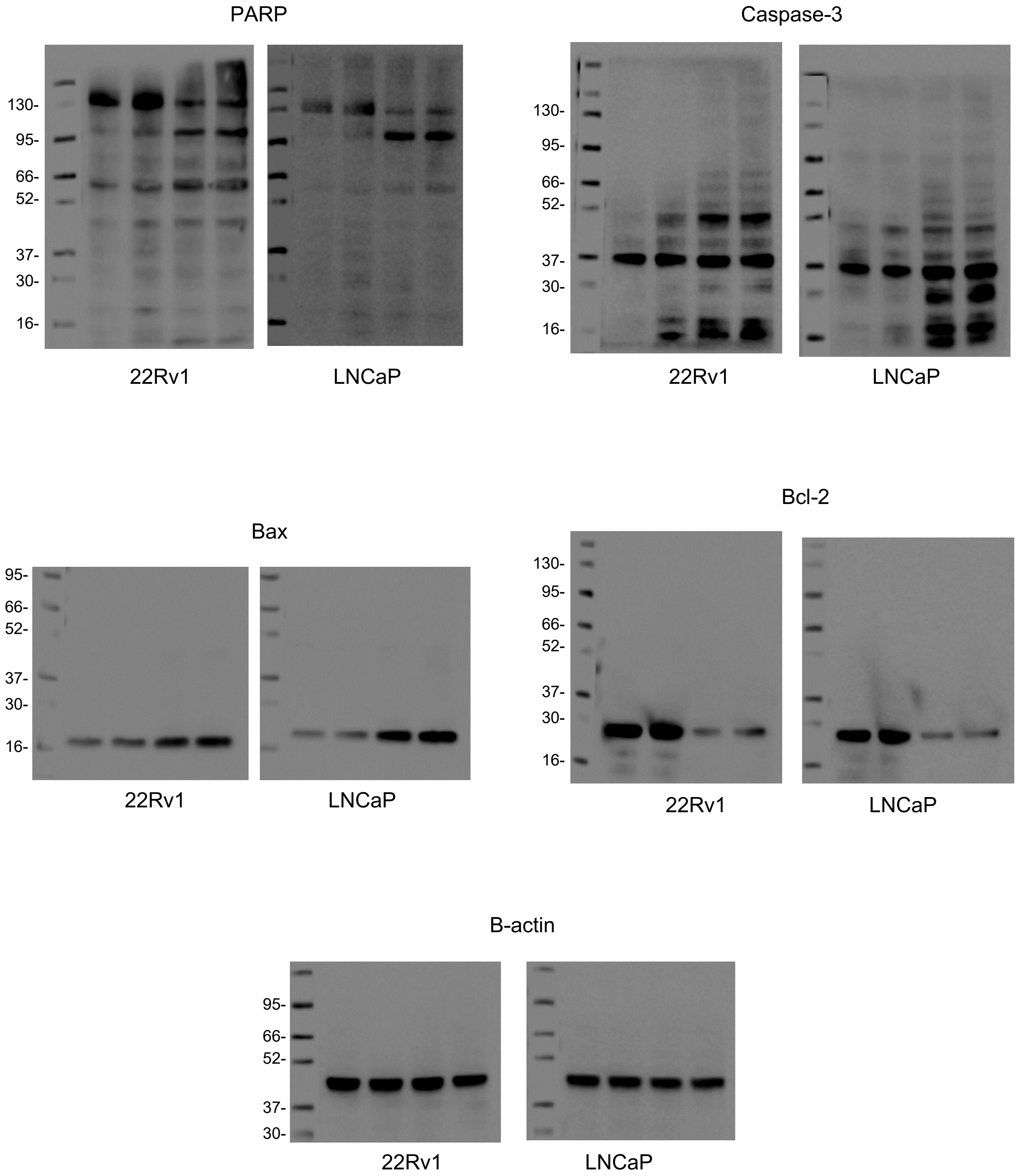
PARP
130-
95-
66-
52-
37-
30-
16-
22Rv1
LNCaP
Caspase-3
130-
95-
66-
52-
37-
30-
16-
22Rv1
LNCaP
Bax
95-
66-
52-
37-
30-
16-
22Rv1
LNCaP
Bcl-2
130-
95-
66-
52-
37-
30-
16-
22Rv1
LNCaP
B-actin
95-
66-
52-
37-
30-
22Rv1
LNCaP

## Figure S8A

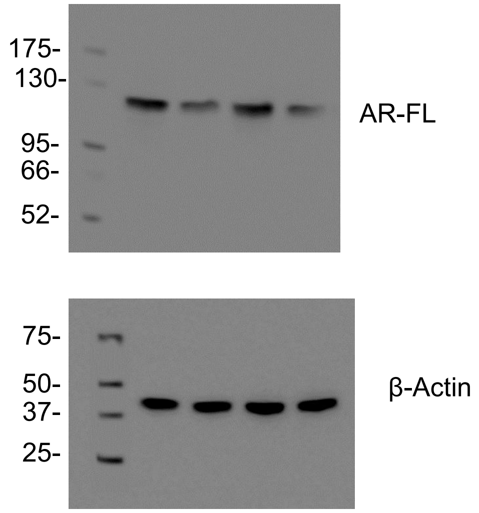


## Figure S8B

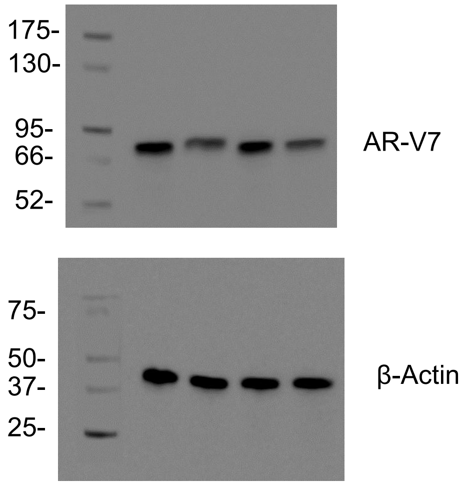


## Figure S8C

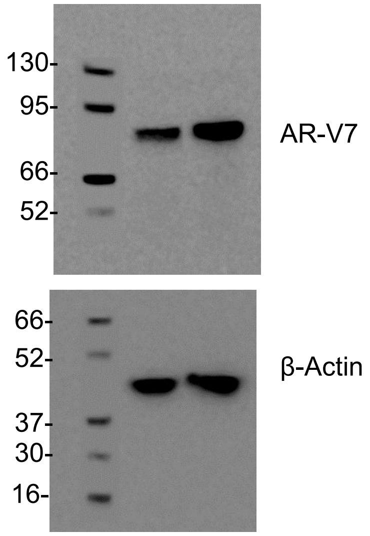


## Figure S8D

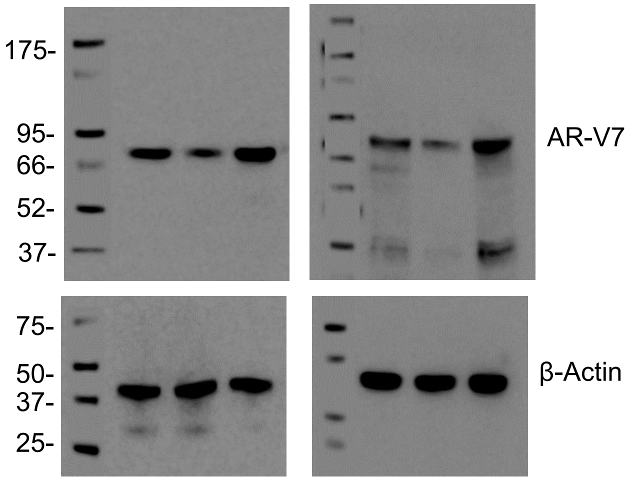

Supplement: Supplementary file 3 — Original western blots [file 41419_2024_7246_MOESM3_ESM.pdf]
